# Quantitative Histomorphometric Analysis of Collagen Bundles in Masson's Trichrome Stained Rat (*Rattus norvegicus*) Skin: A Methodological Study

**DOI:** 10.1002/hsr2.71998

**Published:** 2026-03-08

**Authors:** Okky Husain, Teresa Liliana Wargasetia, Julia Windi Gunadi, Angkasa Ramatuan Hamdan

**Affiliations:** ^1^ Department of Anatomical Pathology Universitas Padjadjaran Bandung Indonesia; ^2^ Unpad Hospital Universitas Padjadjaran Bandung Indonesia; ^3^ Master Program in Skin Ageing and Aesthetic Maranatha Christian University Bandung Indonesia; ^4^ Department of Physiology Maranatha Christian University Bandung Indonesia; ^5^ Maranatha Biomedical Research Laboratory Maranatha Christian University Bandung Indonesia; ^6^ Department of Internal Medicine Universitas Padjadjaran Bandung Indonesia

**Keywords:** collagen analysis, digital image analysis, histology, histomorphometry, histopathology, Masson's trichrome, quantitative pathology

## Abstract

**Background and Aims:**

While Masson's Trichrome is widely used for collagen visualization, its qualitative nature limits reproducibility and statistical comparison. A methodological gap persists in the quantitative assessment of collagen in *Rattus norvegicus* skin, an important model for dermatological research. This study's objective was to develop a novel, quantitative protocol to precisely measure collagen features, enhancing the study of tissue remodeling and fibrosis.

**Methods:**

Formalin‐fixed, paraffin‐embedded skin tissue from Wistar rats was stained with Masson's Trichrome. Digital images were analyzed using ImageJ. The protocol involved creating custom stain vectors for precise color deconvolution, separating the image into blue–green (collagen) and red–pink (non‐collagenous) channels. Quantitative features, including area, intensity, skewness, and kurtosis, were extracted from the thresholded regions of each stain.

**Results:**

Analysis of 168 images showed that the blue–green (collagen) stained area was significantly larger than the red–pink area (*p* < 0.001). The median collagen area was 1.273 mm² compared to 0.313 mm² for the red–pink area. Collagen stain intensity was also significantly higher (*p* < 0.001). Distribution shape analysis confirmed distinct signatures: the blue–green stain was highly symmetrical (skewness about 0.07), while the red–pink stain was platykurtic and positively skewed, reflecting its binding to more heterogeneous components.

**Conclusion:**

This work provides a validated framework for the objective and reproducible quantification of collagen in rat skin. By bridging traditional histology with computational analysis, this protocol offers a robust tool for future studies on collagen dynamics in skin pathologies and wound healing, with potential applications in evaluating therapeutic strategies.

**Abbreviations**
FFPE: formalin‐fixed paraffin embedded
ROI: region of Interest

## 1 Introduction

Collagen fibers serve as the main extracellular matrix component in connective tissue. It acts as a structural scaffold that can direct cell adhesion, migration, and regulate cellular growth and metabolism. Collagen forms the main tension‐resistant element in the tissue. About 28 types of collagen have been found, but over 90% of the collagen in the human body is Type I [1]. Collagen fibers are often examined in histological research concerning wound healing [2], the impact of electromagnetic radiation [3], and fibrosis [4]. During wound healing, collagen is deposited by fibroblasts. Simultaneously, fragmented and degraded collagen promotes fibroblast proliferation and growth factor synthesis that lead to angiogenesis and re‐epithelialization. At the final step, the balance of new matrix synthesis and matrix degradation by matrix metalloproteinases determines the tensile strength of the healed tissue [5].

Masson's trichrome (MT) staining is a pivotal histological technique that differentiates collagen from other tissue elements [6]. MT enables an analysis of the alterations in collagen morphology and the extent of collagen fiber deposition, providing insights into tissue remodeling and repair mechanisms [4, 6, 7]. Routine hematoxylin and eosin (H&E) staining is less selective for collagen. With H&E, collagen bundles, cytoplasm, and muscle are all stained pink by eosin. In addition, eosin also stains keratin, which is present in hair follicles within the dermis and the epidermis, which is adjacent to the dermis. This issue is handled with MT staining. Some studies have utilized picrosirius red; these studies show comparable performance between MT and picrosirius Red [8, 9, 10]. Furthermore, MT does not require polarized light microscopy compared to the latter.

Common rat (*Rattus norvegicus)* skin serves as an invaluable model for studying mechanisms that are relevant to human skin [11]. However, it is important to note that, microscopically, human skin and rat skin exhibit distinct differences. Consequently, unique features such as prominent hair follicles necessitate careful selection of regions of interest (ROI) during image analysis.

Digital microscopic imaging has revolutionized histopathology by enabling the automated analysis of high‐resolution images. Integrating digital image analysis with traditional histological methods enhances the evaluation of intricate tissue structures and their alterations in response to various stimuli. This histomorphometric analysis provides a quantitative histopathological technique to assess changes in tissue and cellular structure and function [12, 13, 14, 15]. This quantitative approach offers advantages over traditional qualitative methods, particularly in terms of the repeatability and reliability of research outcomes. By facilitating more comprehensive analyses through the extraction of quantitative data, this method enhances our understanding of tissue characteristics [16, 17].

MT stain was selected for histomorphometrical analysis, which has been used in various collagenous deposition lesions and processes, such as in the lung [18], kidney [19], liver [20], and colon [21]. Despite these advancements and the proven utility of MT in qualitative assessment, a methodological gap persists in quantitatively evaluating collagen features from MT in rat skin dermis. This study aims to leverage digital image analysis and feature extraction to quantify MT‐stained rat skin connective tissue. This study bridges traditional histochemistry with computational tools to quantify collagen characteristics in rat skin, offering insights into pathological and reparative processes.

The primary objective of this study is to establish a standardized, reproducible protocol for quantifying collagen features in MT‐stained rat skin connective tissue using digital image analysis.

## 2 Materials and Methods

### 2.1 Specimen Handling and Staining

The Research Ethics Committee of the Faculty of Medicine at Universitas Kristen Maranatha has issued Decision Letter No. 178/KEP/XI/2024, which confirms that the parent research concerning the Wistar strain rat specimen has been approved and authorized for this study.

The tissue samples were obtained from the skin of anesthetized common rats (*Rattus norvegicus*), specifically the Wistar strain. The rats were anesthetized with a combination of xylazine and ketamine. The tissues were fixed in 10% neutral buffered formalin, dehydrated through increasing concentrations of ethanol, cleared with xylene, and infiltrated with paraffin wax. In the formalin‐fixed paraffin‐embedded (FFPE) tissue block, the tissue is oriented perpendicular to the block surface, allowing for visualization of the entire thickness of the skin under a microscope.

MT staining was conducted on FFPE tissue as follows: The staining procedure commenced with preheating a bead bath within a fume hood to 60°C. Bouin's solution was also heated in a glass container within the bead bath to 60°C. The slides were deparaffinized using an automated Gemini AS stainer and subsequently immersed in Bouin's solution for 1 h at 60°C. After discarding the Bouin's solution into labeled waste, the slides were rinsed under running tap water for 5 min to remove any residual solution.

Tissue sections were stained with Weigert's iron hematoxylin for 10 min, rinsed, and counterstained with Biebrich Scarlet‐Acid Fuchsin for 5 min. The slides were then treated with phosphotungstic/phosphomolybdic acid for 10 min to enhance collagen differentiation, followed by Aniline Blue staining for 5 min to stain collagen fibers. Excess dye was removed by rinsing three times with distilled water. Tissue differentiation was achieved by incubating the slides in 1% acetic acid for 1 min, followed by a final rinse with distilled water. Dehydration was performed using graded ethanol (95% and 100%, 2 min each), followed by xylene clearing for 2 min. Finally, the slides were coverslipped with mounting medium for microscopic analysis.

### 2.2 Digital Image Processing and Analysis

The slides were photographed to create digital images using a camera‐equipped microscope. We utilized the Olympus CX43 microscope paired with a Scopepad‐LX105 camera mounted on an Olympus 0.5X C‐Mount adapter. The highest magnification that visualized the entire layers of skin was then used for acquiring microphotographs to ensure consistency and eliminate potential bias from local histological variation, such as hair follicles or sebaceous glands. This comprehensive measurement provides a more representative assessment of the global collagen distribution within the tissue.

Further quantitative histomorphometric analysis was conducted using ImageJ (version 1.54j). Initially, each digital photomicrograph underwent color deconvolution using the Colour Deconvolution2 version 2.1 plugin to digitally separate the stains. To enhance accuracy, we generated custom stain vectors directly from the images rather than using the software's default presets. The blue–green vector, representing collagen, was defined by selecting a region of interest (ROI) within the dermis. The red–pink vector, representing muscle and cytoplasm, was defined by selecting an ROI over the panniculus carnosus muscle. This process resulted in two separate 8‐bit images, one for each color channel, isolating the blue–green collagenous and non‐collagenous components for analysis.

A binary mask of the connective tissue was created to define the total area for analysis. This process began with the separated blue–green (collagen) channel, to which a Gaussian blur with a sigma of 2.00 pixels was applied. The resulting image was then converted into a binary image using the “Default” auto‐thresholding method in ImageJ (version 1.54j), with the “ignore black” option enabled. Finally, a binary closing operation was performed on this mask to consolidate the tissue area by filling the natural spaces between collagen bundles.

In parallel, both the blue–green and red–pink deconvoluted images were segmented based on staining intensity. This also used the “Default” auto‐thresholding method, with the “setThreshold” and “ignore black” options activated. The final step involved using the Image Calculator to perform an “AND” operation, which combined the preprocessed binary mask with each of the two thresholded stain images. This ensured that subsequent analysis was restricted to the stained tissue within the designated mask boundaries.

From these final combined images, a comprehensive set of quantitative features was measured. The parameters, limited to the thresholded regions, included the area, mean gray value, median, standard deviation, minimum and maximum gray value, skewness, and kurtosis. The ratio between each stained area and the total mask area was also calculated. These extracted data were then used for further descriptive and analytical statistical analyses to correlate with the qualitative features of rat dermal morphology. A detailed guide for this procedure can be found in Supporting Information S1: File S1.

All statistical analyses were conducted using Python with the scipy.stats (version 1.16.0) and the seaborn libraries. A two‐step approach was used to compare the quantitative features between the blue–green and red–pink stain groups. Initially, the distribution of each variable was assessed for normality using the Shapiro–Wilk test. A feature was considered normally distributed if the test yielded a *p* value greater than 0.05. The choice of the comparison test was based on the normality results. If both groups followed a normal distribution, an independent samples *t*‐test was performed. If at least one of the groups did not follow a normal distribution, the non‐parametric Mann–Whitney *U* test was used. Furthermore, linear regression analysis was used to assess the strength and direction of the relationship between paired blue–green and red–pink features, with the coefficient of determination (R‐squared) and p‐value being reported. For all tests, a *p* value of less than 0.05 was considered statistically significant.

## 3 Results

Following the image acquisition process, a total of 168 digital images capturing the complete thickness of the skin of 28 rats were obtained. Each image possesses a red–green–blue color depth of 24 bits, with dimensions of 1832 pixels in width and 1321 pixels in height. The objective lens is magnified at 4×, enabling the visualization of the entire skin thickness in a single image.

**Figure 1:**
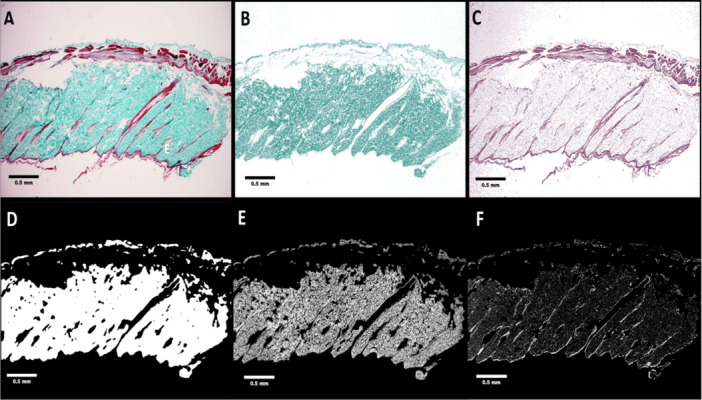
Step‐by‐step digital image processing. (A) The original acquired skin thickness. The result of deconvoluted (B) blue–green stain and (C) red–pink stain of original image. (D) Preprocessed binary image of Mask. Result of image calculator AND operation of mask with (E) blue–green stain and (F) red–pink stain.

Measurements indicate that 1 mm in the image corresponds to 473 pixels. This implies that each image represents an area of approximately 3.86 mm in width and 2.60 mm in height, with each pixel length equating to 0.20 micrometers. All images were captured with the objective lens magnification set at 4×, ensuring that the entire thickness of the skin—from the epidermis to the connective tissue beneath the muscularis—is visible, as exemplified in Figure 1A.

Color deconvolution was performed on all images. Initially, the vector values for color deconvolution utilized the MT vector provided by ImageJ Color Deconvolution plugin. However, improved staining differentiation was achieved by selecting a specific ROI. The blue–green stain vector was extracted by selecting the collagenous area of the dermis, while the red–pink stain vector was extracted by selecting the muscular region of interest (ROI). The vectors are presented in Table 1.

**Table 1:** Color deconvolution vectors for each color.

	Colors	*R* value	*G* value	*B* value
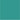	Blue–green	0.8001	0.4070	0.4407
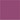	Red–pink	0.4311	0.7154	0.5498

After performing color deconvolution based on the vectors, two different images were generated. Figure 1B shows the result of selecting the green–blue color, which represents the fibrous tissue, while Figure 1C illustrates the red–pink color representing the plasma stain.

A mask representing the fibrous connective tissue was generated based on the fibrous stain in each image, with a median mask area of 2.261 mm^2^(range: 1.420–3.119 mm^2^). This area represents a mask‐to‐image ratio of 40.0 percent (range: 25.1%–55.1%). As observed, the majority of the mask area corresponds to the dermis, with a minority representing the deeper connective tissue beneath the muscular tissue. Due to the selective staining of fibrous tissue, hair follicles, sebaceous glands, epidermis, and muscle were excluded from the mask. The stain was filtered with a blur to include the “natural cracks” between bundles of collagen in the dermis, followed by a binary closing algorithm. The resulting mask is shown in Figure 1D. The area of the selected mask was then recorded. The results of the operation between the mask and the fibrous stain are displayed in Figure 1E, while the mask with the plasma stain is shown in Figure 1F.

**Figure 2:**
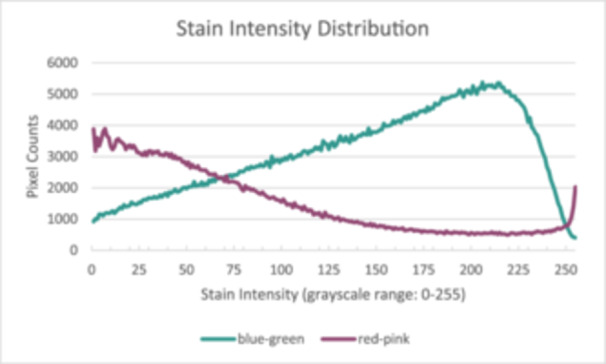
The stain intensity distribution.

The distribution of stain intensity features resulting from the AND operation can be extracted. Figure 2 illustrates an example of the stain intensity distribution presented in Figure 1E and Figure 1F. The characteristics of the masked area and the thresholded area are measured and extracted. In addition, stain intensity metrics—including minimum, maximum, median, mean intensity, and standard deviation—are calculated for both blue–green fibrous and red–pink plasma stains to represent the stain intensity distribution. The extracted descriptive quantitative data of the tissues’ features are shown in the Table 2. The normality test for each feature's distribution is available in Supporting Information S2: File S2, and the comparative histograms for each feature are available in Supporting Information S3: File S3.

**Table 2:** Extracted features of tissues’ features between stains.

Features^a^	Blue–green	Red–pink	*p*
Area features			
Thresholded area (mm^2^)	1.27 [1.02–1.54]	0.31 [0.26–0.37]	< 0.001
Thresholded to mask area ratio (%)	57.2 ± 6.9	13.9 ± 3.81	< 0.001
Intensity features (grayscale: 0–255)			
Mean intensity	184.8 ± 10.5	177.3 [171.1–184.4]	0.015
Median intensity	185 [176–195]	177 [169–186]	< 0.001
Minimum intensity	131 [122–138]	115 [108–122]	< 0.001
Maximum intensity	255 [255–255]	255 [255–255]	0.99
Distribution shape features			
Skewness	0.07 ± 0.31	0.18 ± 0.29	< 0.001
Kurtosis	−0.96 [−1.11 to (−0.81)]	−1.33 [−1.47 to (−1.18)]	< 0.001

^a^ Data are presented as mean ± SD for normally distributed data or median [interquartile range] for non‐normally distributed data. *p* values were derived from an independent *t*‐test or a Mann–Whitney *U* test based on data distribution.

Based on the extracted features presented in Table 2, the median area of the mask representing the dermis is 2.261 mm² (range: 1.42–3.12 mm²). This area constitutes between 25.1% and 55.1% of the total image area. The remaining excluded areas are attributed to brightfield. Further evaluation indicates that the area of the red–pink thresholded region had a median of 0.313 mm² (range: 0.182–0.533 mm²). The Mann–Whitney *U* test was applied, which confirmed that the area of the blue–green stain was significantly larger than that of the red–pink stain (*p* < 0.001) with an average difference of 0.97 mm² (SD: 0.21 mm²). Upon measuring the ratio between the red–pink and blue–green thresholded areas, the median was found to be 24.5% (range: 13.5%–40.0%). These ratios correspond to the dermal features that predominantly exhibit blue–green staining of collagen bundles.

For stain intensity, the median, minimum, and maximum intensity of both stain thresholded images are recorded and presented in Table 2. The medians of the blue–green stain are significantly (*p* < 0.05) higher by approximately 9 scores than those of the red–pink stain. Meanwhile, no difference in the maximum value was observed, as all stained regions scored at 255, which is the maximum integer value in an 8‐bit depth image. The Mann–Whitney *U* test revealed that the blue–green stain had a significantly higher median intensity (*p* < 0.001) and mean intensity (*p* = 0.015).

The background and excluded tissues that are not predominantly stained with blue–green color include muscular tissue, the epidermis, and the pilosebaceous unit of the skin's adnexa. The mask‐to‐image ratio should be evaluated, as this feature indicates how well the microphotograph represents the entire thickness of the skin. If the ratio is too low, the dermis may be underrepresented in the image; conversely, if the ratio is too high, it is possible that only a section of the entire skin tissue is depicted in the image.

After performing thresholding, parts of the mask area were excluded from the images. Upon further evaluation, after adding the red–pink thresholded area to the blue–green thresholded area, the sum was less than the mask area, with the ratio ranging from 56.3% to 89.1% and a median of 70.8%. These gaps are attributed to unstained areas between collagen bundles.

As evaluated in Figure 2, our analysis showed that both the blue–green and red–pink stains exhibited negative kurtosis, indicating platykurtic, or “flat,” distributions. The median kurtosis for the blue–green stain was −0.96, while the red–pink stain was significantly more platykurtic with a median of −1.33 (*p* < 0.001). A platykurtic distribution signifies a wide range of staining intensities rather than a single, sharply defined peak. This is expected in a complex biological sample. The significantly more negative kurtosis of the red–pink stain suggests a very broad and heterogeneous distribution of stain intensities.

This finding quantitatively supports the observation that the red–pink channel captures a diverse array of components: the weakly stained cytoplasm of dermal cells as well as the strongly stained keratin in hair follicles and muscle tissue at the dermal boundary. This mix of very weak and very strong signals flattens the overall distribution, resulting in a more negative kurtosis compared to the more uniform collagen staining. These distributions explain the significant (*p* < 0.05) difference in the red–pink stain distribution, which has a lower negative value with a median of −1.331 (range: −1.466 to −0.424) compared to the blue–green stain distribution, which has a median of −0.962 (range: −1.110 to −0.202), with an average difference of −0.320.

The skewness analysis revealed a key difference in the symmetry of the two stain distributions. The blue–green stain that corresponds with the collagen had a mean skewness of 0.07, a value very close to zero, indicating a highly symmetrical distribution. This suggests that the collagen stain intensities are evenly balanced around a central mean, reflecting the relatively uniform and homogenous nature of the collagen bundles throughout the dermis. In contrast, the red–pink stain showed a small but statistically significant positive skew, with a mean of 0.18 (*p* < 0.001). A right‐skewed distribution means that while the bulk of the data points have lower intensity, there is a “tail” of fewer data points with very high intensity. This perfectly aligns with the kurtosis finding; the majority of the masked dermal area contains weakly stained red–pink cytoplasm, while a smaller portion contains intensely stained elements like muscle fibers, which pull the distribution's tail to the right.

In summary, the distribution shape features quantitatively confirm that the staining protocol successfully captures two distinct biological signatures. The blue–green stain is characterized by a stable, symmetrical distribution reflective of homogenous collagen, whereas the red–pink stain's platykurtic and positively skewed distribution reflects its binding to a more varied and complex set of non‐collagenous tissues.

**Figure 3:**
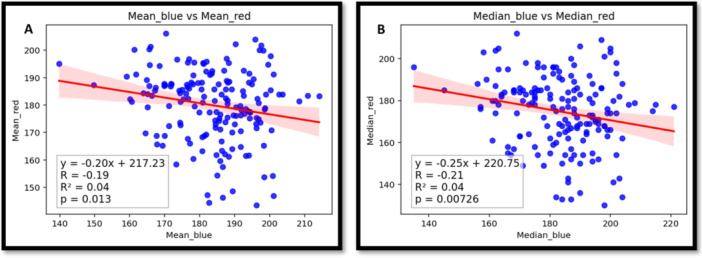
The linear regression of mean intensity (A) and median intensity (B) between two colors.

By comparing the central tendencies of the two stain groups, performing linear regression analysis provided critical insight into the relationship between the collagenous (blue–green) and non‐collagenous (red–pink) components within the dermal matrix. Our analysis revealed that while some correlations were statistically significant (*p* < 0.05), the coefficients of determination (*R* ^2^) were consistently low across all measured features (see Supporting Information S4: File S4). For example, the relationship between the blue–green area and the red–pink area, though statistically significant (*p* < 0.001), yielded an *R* ^2^ value of only 0.24. Similarly, weak correlations were observed for other features, such as mean and median intensity, as in Figure 3.

This finding is particularly valuable for two reasons. First, it provides a quantitative validation of the MT staining and color deconvolution methodology. A low *R* ^2^ value demonstrates that the two‐color channels are independent of one another. This confirms that the protocol is successfully differentiating and measuring two distinct biological components rather than capturing redundant information.

Second, this observed independence reinforces the utility of this protocol for studying complex tissue remodeling processes like wound healing or fibrosis. It suggests that a change measured in the blue–green channel (collagen) can be interpreted as a specific alteration of the collagenous matrix, rather than a non‐specific change that affects all tissue components equally. Therefore, the linear regression results give us confidence that this quantitative approach can be used to independently track the dynamics of both the collagen matrix and the surrounding cellular or muscular elements within the dermis.

## 4 Discussion

### 4.1 Digital Image Analysis of MT

MT is preferred over routine H&E for collagen evaluation due to its superior selectivity. MT is repeatedly selected to evaluate collagen fibers in various settings, such as lung fibrosis [4], vessel walls [22], and intestine [23].

ImageJ is a powerful, open‐source image processing software that supports a wide range of plugins that extend its capabilities [24]. In our study, the color deconvolution plugin is used to separate different stains in histological images [25] and quantify the ROI [26].

The blue–green color in Masson's selective trichrome highlights the collagen fibers, which are predominantly located in the connective tissue of the dermis to provide mechanical strength. By performing color deconvolution, non‐collagenous regions were excluded from the analysis [25]. Research by Van de Vlekkert et al. indicates that immature collagen fibers appear as a paler blue or green compared to the darker‐stained mature fibers, therefore necessitating the extraction of color intensity features [6].

The red–pink coloration in MT stains the cytoplasm, keratin, fibrin, and erythrocytes [6]. However, as illustrated in Figure 1C, the red–pink stain remains evident in the dermal cutaneous region and may represent the cytoplasm of dermal cells.

In the context of color deconvolution, custom stain vectors demonstrated superior performance over software presets, likely due to batch effects, resulting from variations in reagent concentrations or lighting during image capture [27, 28]. These findings emphasize the importance of maintaining procedural homogeneity to ensure the validity of the vector across the entire dataset. The limitations of qualitative histological assessment include interobserver variability [29, 30, 31, 32]. By providing objective, reproducible measurements, this approach enhances the reliability of histological assessments and mitigates the subjective bias and observer variability inherent in qualitative methods.

### 4.2 MT Stain Compared to Other Collagen Stains

There are several alternative tissue stains that also highlight collagen fibers, such as picrosirius red, van Gieson stain, Azan stain, and Mallory's trichrome stain. While Picrosirius red is able to differentiate Type I collagen from Type III collagen via birefringence under polarized microscopy, this microscope is often not available in most clinical facilities [10]. Therefore, MT offers better accessibility for most laboratories. Although further study is needed to confirm whether MT quantitative features can differentiate between collagen Type I and Type III as effectively as picrosirius red, MT provides superior contrast by distinctly staining muscle/cytoplasm red/pink and collagen blue/green [9].

The Van Gieson method, while effective, is more limited in its color range, primarily showing red and yellow, thereby providing less contrast compared to MT. Azan stain and Mallory's stain are more complex with longer protocols and more steps involved, and also require fixation in specific mordants, such as Zenker's or Bouin's, which are not standard for most laboratories.

### 4.3 Rat Skin Dermis Features

Skin provides a physical barrier from the outer world, consisting of epidermis, dermis, and subcutaneous fat [33]. The dermis is a connective tissue layer providing skin with durability, strength, and flexibility. It comprises two parts: the thin superficial papillary dermis for epidermal interdigitation, and the thicker, deeper reticular dermis, providing strength to the skin [33]. The thickness of the dermis varies, with the thickest skin found on the back and the thinnest on the eyelids [28]. Collagen fibers account for three‐fourths of the total fibers in dermal connective tissue. They appear as bundles, with fibroblasts in between. Collagen fibers significantly contribute to the skin's tensile strength. Other fibers, such as elastin fibers, enclose the collagen bundles, while reticulin fibers are composed of collagen fibrils [33, 34]. Elastin fibers are typically visualized using the Verhoeff stain method, while reticulin fibers are visualized using Gomori's silver impregnation stain [6]. Elastin and reticulin may be responsible for the ‘natural cracks’ observed in the unstained regions between collagen bundles.

Unlike human skin, rat skin possesses an additional layer known as the panniculus carnosus muscle that lies beneath the dermis and serves as a reference ROI for generating the red–pink stain color vector [35, 36].

### 4.4 Limitations, Applicability, and Future Directions of the Study

A primary limitation of this study is the potential “batch effect,” where staining variations may occur across different tissue sample sets [37]. Discrepancies in reagent concentrations or lighting conditions can affect the results, necessitating single batch processing to ensure consistency. While this study minimized this issue using a uniform batch and color vector, the results may not be universally applicable to other datasets. Future research must ensure strict procedural homogeneity across different batches and implement inter‐batch normalization [28]. Another limitation of the present study is the absence of documented data regarding the sex of the rat subjects. The primary focus of this methodological analysis remained on the general comparative stain intensity and the validation of the quantification protocol across experimental groups.

MT cannot visualize all extracellular matrix components; unstained areas between collagen bundles could potentially be elastin or reticulin fibers, requiring different staining methods. MT does not differentiate between types of collagens. Future research should investigate whether quantitative features extracted from MT, such as staining intensity, can correlate with different collagen types, providing an alternative to methods like Picrosirius Red [10].

The robust framework established in this study facilitates reproducibility in future histological studies focusing on collagen dynamics. By providing an objective and quantifiable methodology, this protocol overcomes the limitations of traditional qualitative analysis, such as inter‐observer variability and subjective bias [38], and allows for the comparison of features previously unseen by traditional qualitative analysis [39]. The ability to precisely measure collagen dynamics and distribution is vital for developing and evaluating targeted therapeutic strategies aimed at modulating tissue remodeling and repair processes, such as in wound healing and fibrosis.

## 5 Conclusions

The results of this study affirm the utility of MT staining combined with advanced image analysis in elucidating the structural characteristics of collagen in rat skin. The ability to quantitatively assess collagen distribution enhances our understanding of its role in various physiological and pathological processes. This research lays the groundwork for future explorations into the dynamics of collagen in skin health and disease, ultimately contributing to improved therapeutic approaches.

## Author Contributions

**Okky Husain:** software, formal analysis, writing – original draft, investigation, visualization **Teresa Liliana Wargasetia:** conceptualization, resources, supervision, writing – review and editing. **Julia Windi Gunadi:** conceptualization, resources, supervision, writing – review and editing. **Angkasa Ramatuan Hamdan:** writing – review and editing.

## Conflicts of Interest

The authors declare no conflicts of interest.

## Transparency Statement

The lead author, Okky Husain, affirms that this manuscript is an honest, accurate, and transparent account of the study being reported; that no important aspects of the study have been omitted; and that any discrepancies from the study as planned (and, if relevant, registered) have been explained.

## Supporting information

results combined final 2.

S1 step by step procedure.

S2 individual histogram of stain features and normality test.

S3 comparative histogram of features between blue green and red pink stain.

S4 linear regression of features between blue green and red pink.

statistical test results table.

## Data Availability

The data is available from the corresponding author upon reasonable request.
