# Supplementary material for: Quantitative Histomorphometric Analysis of Collagen Bundles in Masson's Trichrome Stained Rat (Rattus norvegicus) Skin: A Methodological Study
Source: Health Sci Rep. 2026 Mar 8;9(3):e71998. doi: 10.1002/hsr2.71998 (PMC12967519; doi:10.1002/hsr2.71998)
Supplement: Supplementary file 2 — S1 step by step procedure. [file HSR2-9-e71998-s006.pdf]

Supplementary Material: A Tutorial for Quantitative Histomorphometric Analysis using ImageJ

Okky Husain, Teresa Liliana Wargasetia, Julia Windi Gunadi, Angkasa Ramatuan Hamdan

This document provides a step-by-step guide for performing quantitative histomorphometric analysis on Masson's Trichrome stained rat skin tissue using the ImageJ software. This protocol is designed to replicate the methods described in the main article and aims to ensure the reproducibility of the results.

Explanation of Quantification Steps with ImageJ

The following is a visual and textual guide to perform collagen quantification based on the workflow described in the study.

Step 1: Color Deconvolution

The objective of this step is to digitally separate the different color components in the histological image. For Masson's Trichrome staining, we want to separate the **blue-green** color (representing collagen) from the **red-pink** color (representing cytoplasm, keratin, and muscle).

1. **Open Image:** Open the microscopic image file (*.png or .tiff*) in ImageJ.
2. **Access Deconvolution Plugin:**
   - Navigate to the menu **Image > Color > Colour Deconvolution 2**. (As seen in *Screenshot 1*).
3. **Select Staining Vectors:**
   - The "Colour Deconvolution 2 window will appear.
   - In the Vectors dropdown menu, you may select **Masson Trichrome**. (screenshot 2)
   - **Important Note:** As explained in the main article, default vectors may not be perfect

due to variations in the staining process (batch effect). For higher accuracy, it is recommended to create custom vectors by selecting a Region of Interest (ROI) on a pure collagen area (for the blue-green vector) and a muscle area (for the red-pink vector), as was done in this study. The result of deconvolution using custom vectors from an ROI is shown in *Screenshot 4*.

4. **Execute and Review Results:**
   - Click **OK**.
   - ImageJ will generate new images representing each separated color channel (As seen in *Screenshot Page 3 & 4*). We will focus on the **blue-green** deconvoluted image (referred to as Colour_1 or blue-green deconvoluted in the following steps) for collagen analysis.

Visualization of Step 1: From the original image (left) to separated images for blue-green (middle) and red-pink (right) components.

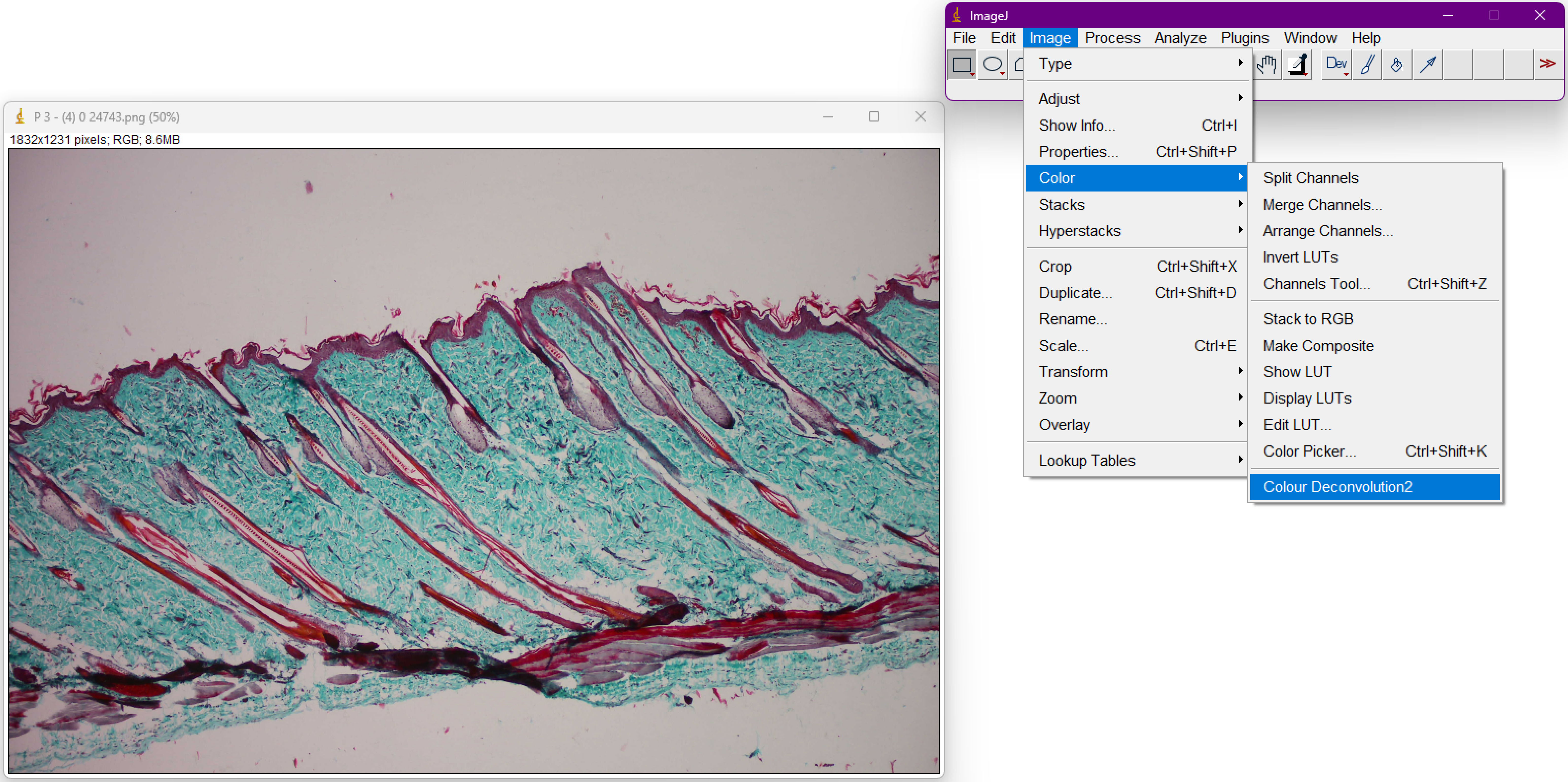
ImageJ
File Edit Image Process Analyze Plugins Window Help
P 3 - (4) 0 24743.png (50%)
1832x1231 pixels; RGB; 8.6MB
Type
Adjust
Show Info... Ctrl+I
Properties... Ctrl+Shift+P
Color
Stacks
Hyperstacks
Crop Ctrl+Shift+X
Duplicate... Ctrl+Shift+D
Rename...
Scale... Ctrl+E
Transform
Zoom
Overlay
Lookup Tables
Split Channels
Merge Channels...
Arrange Channels...
Invert LUTs
Channels Tool... Ctrl+Shift+Z
Stack to RGB
Make Composite
Show LUT
Display LUTs
Edit LUT...
Color Picker... Ctrl+Shift+K
Colour Deconvolution2

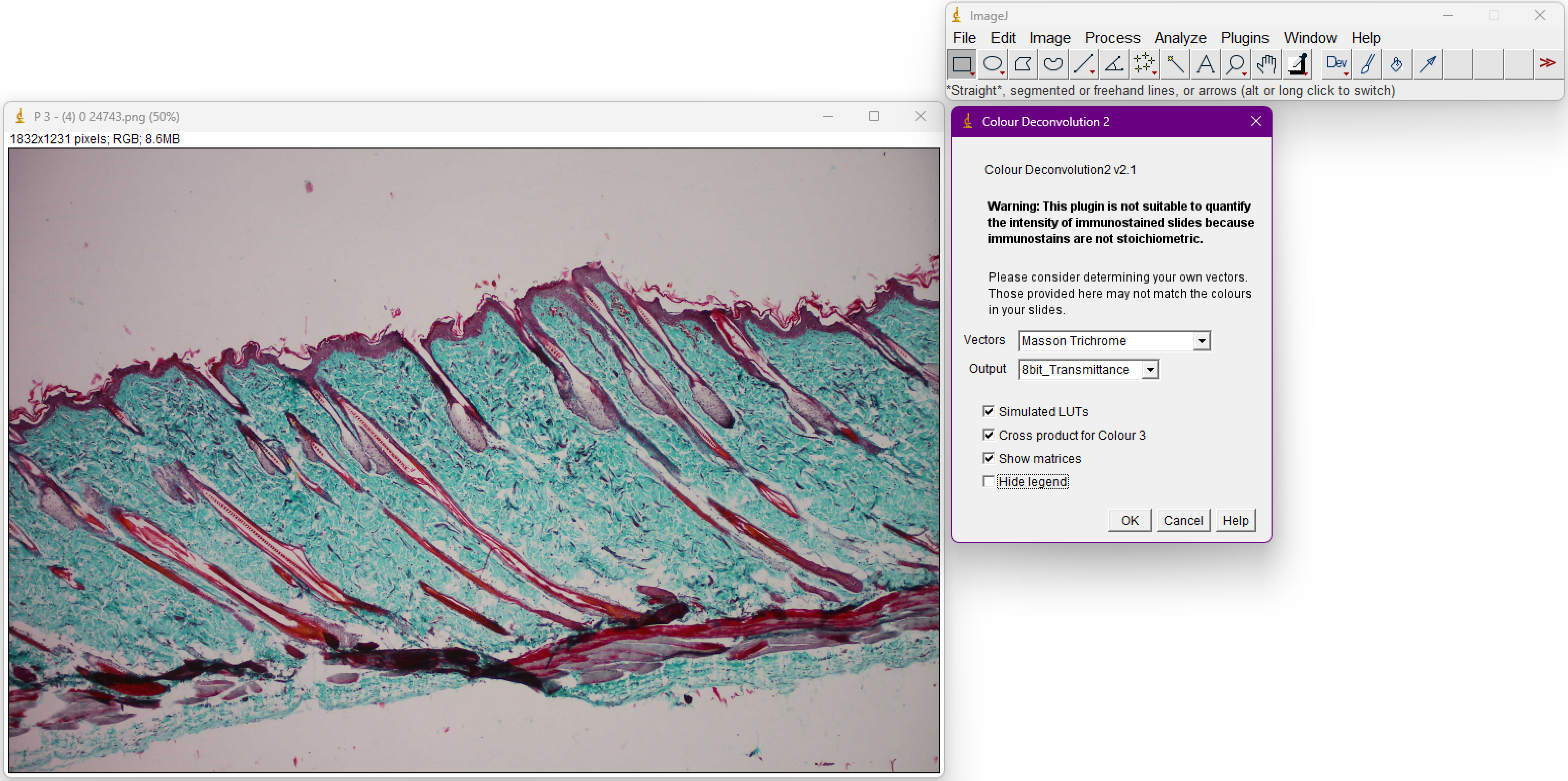
ImageJ
File Edit Image Process Analyze Plugins Window Help
Straight, segmented or freehand lines, or arrows (alt or long click to switch)
P 3 - (4) 0 24743.png (50%)
1832x1231 pixels; RGB; 8.6MB
Colour Deconvolution 2
Colour Deconvolution2 v2.1
Warning: This plugin is not suitable to quantify the intensity of immunostained slides because immunostains are not stoichiometric.
Please consider determining your own vectors. Those provided here may not match the colours in your slides.
Vectors Masson Trichrome
Output 8bit_Transmittance
Simulated LUTs
Cross product for Colour 3
Show matrices
Hide legend
OK Cancel Help

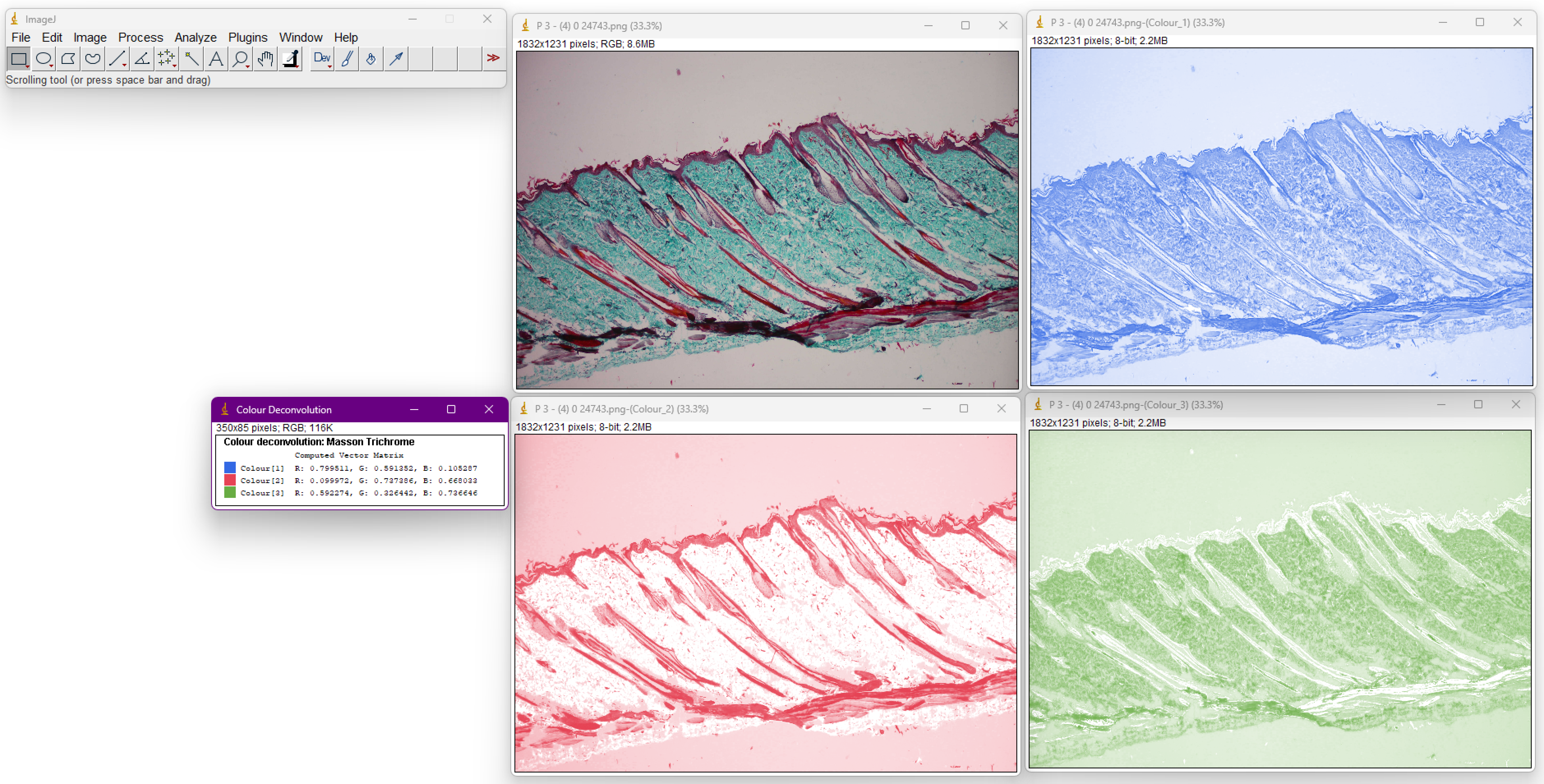
ImageJ
File Edit Image Process Analyze Plugins Window Help
Scrolling tool (or press space bar and drag)
P 3 - (4) 0 24743.png (33.3%)
1832x1231 pixels; RGB; 8.6MB
P 3 - (4) 0 24743.png-(Colour_1) (33.3%)
1832x1231 pixels; 8-bit; 2.2MB
Colour Deconvolution
350x85 pixels; RGB; 116K
Colour deconvolution: Masson Trichrome
Computed Vector Matrix
Colour[1] R: 0.799511, G: 0.591352, B: 0.105287
Colour[2] R: 0.099972, G: 0.737386, B: 0.668033
Colour[3] R: 0.592274, G: 0.326442, B: 0.736646
P 3 - (4) 0 24743.png-(Colour_2) (33.3%)
1832x1231 pixels; 8-bit; 2.2MB
P 3 - (4) 0 24743.png-(Colour_3) (33.3%)
1832x1231 pixels; 8-bit; 2.2MB

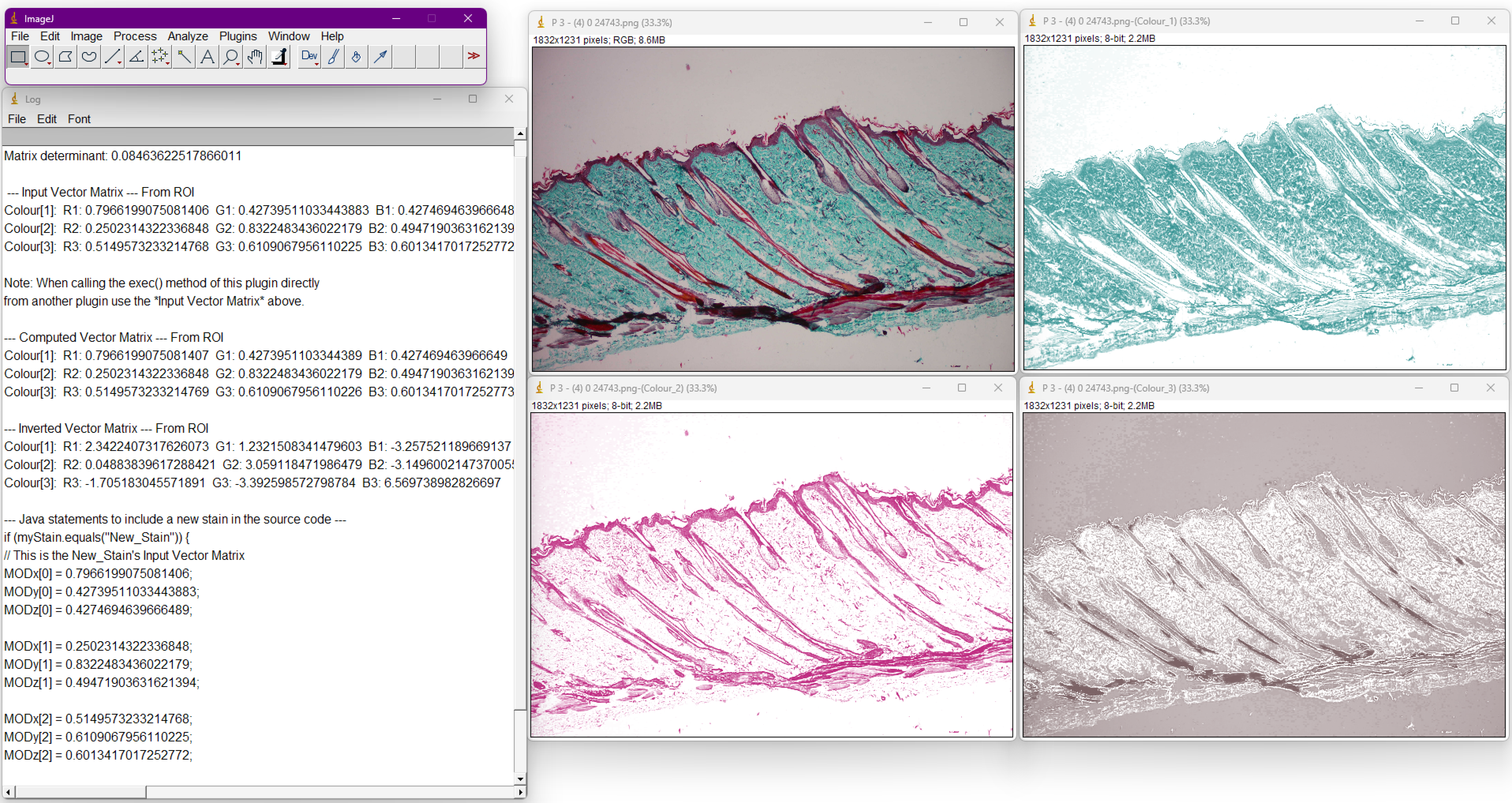
ImageJ
File Edit Image Process Analyze Plugins Window Help
Log
File Edit Font
Matrix determinant: 0.08463622517866011
--- Input Vector Matrix --- From ROI
Colour[1]: R1: 0.7966199075081406 G1: 0.42739511033443883 B1: 0.427469463966648
Colour[2]: R2: 0.2502314322336848 G2: 0.8322483436022179 B2: 0.4947190363162139
Colour[3]: R3: 0.5149573233214768 G3: 0.6109067956110225 B3: 0.6013417017252772
Note: When calling the exec() method of this plugin directly
from another plugin use the *Input Vector Matrix* above.
--- Computed Vector Matrix --- From ROI
Colour[1]: R1: 0.7966199075081407 G1: 0.4273951103344389 B1: 0.427469463966649
Colour[2]: R2: 0.2502314322336848 G2: 0.8322483436022179 B2: 0.4947190363162139
Colour[3]: R3: 0.5149573233214769 G3: 0.6109067956110226 B3: 0.6013417017252773
--- Inverted Vector Matrix --- From ROI
Colour[1]: R1: 2.3422407317626073 G1: 1.2321508341479603 B1: -3.257521189669137
Colour[2]: R2: 0.04883839617288421 G2: 3.059118471986479 B2: -3.14960021473700
Colour[3]: R3: -1.705183045571891 G3: -3.392598572798784 B3: 6.569738982826697
--- Java statements to include a new stain in the source code ---
if (myStain.equals("New_Stain")) {
// This is the New_Stain's Input Vector Matrix
MODx[0] = 0.7966199075081406;
MODy[0] = 0.42739511033443883;
MODz[0] = 0.4274694639666489;
MODx[1] = 0.2502314322336848;
MODy[1] = 0.8322483436022179;
MODz[1] = 0.49471903631621394;
MODx[2] = 0.5149573233214768;
MODy[2] = 0.6109067956110225;
MODz[2] = 0.6013417017252772;
P 3 - (4) 0 24743.png (33.3%)
1832x1231 pixels; RGB; 8.6MB
P 3 - (4) 0 24743.png-(Colour_1) (33.3%)
1832x1231 pixels; 8-bit; 2.2MB
P 3 - (4) 0 24743.png-(Colour_2) (33.3%)
1832x1231 pixels; 8-bit; 2.2MB
P 3 - (4) 0 24743.png-(Colour_3) (33.3%)
1832x1231 pixels; 8-bit; 2.2MB

Step 2: Creating the Dermal Mask

The purpose of this step is to create a binary (black and white) image that specifically defines the dermal area (fibrous connective tissue) as the region of interest (ROI) This mask will ensure that the analysis is performed only within the dermis.

1. **Use the Blue-Green Image:** Select the window containing the blue-green deconvoluted image.
2. **Convert to Binary Image:**
   - Navigate to the menu
     Process > Binary > Make Binary.

   - The image will be converted to black and white. Strongly stained areas will become white, and the background will become black. (screenshot 5)
3. **Apply Gaussian Blur:**
   - Navigate to the menu
     Process > Filters > Gaussian Blur....

   - This step helps to smooth the image and reduce noise, which facilitates the creation of a more cohesive binary mask (screenshot 8).

4. **Apply "Close-" Morphological Operation:**
   - Navigate to the menu **Process > Binary > Close (screenshot 9)**

   - This operation is crucial for filling small holes or gaps within the dermal area (referred to as 'natural cracks' in the article), resulting in a single, solid mask area (As seen in *Screenshot Page 9, 10 & 11*).
5. **Final Mask Result:**
   - You now have a clean binary mask where the white area represents the entire dermis to be analyzed. (As seen in the preprocessed mask image in *Screenshot Page 19*).

Visualization of Step 2: From the blue-green image (left), blur, binarization, and 'close-' are applied to generate a solid dermal mask (right).

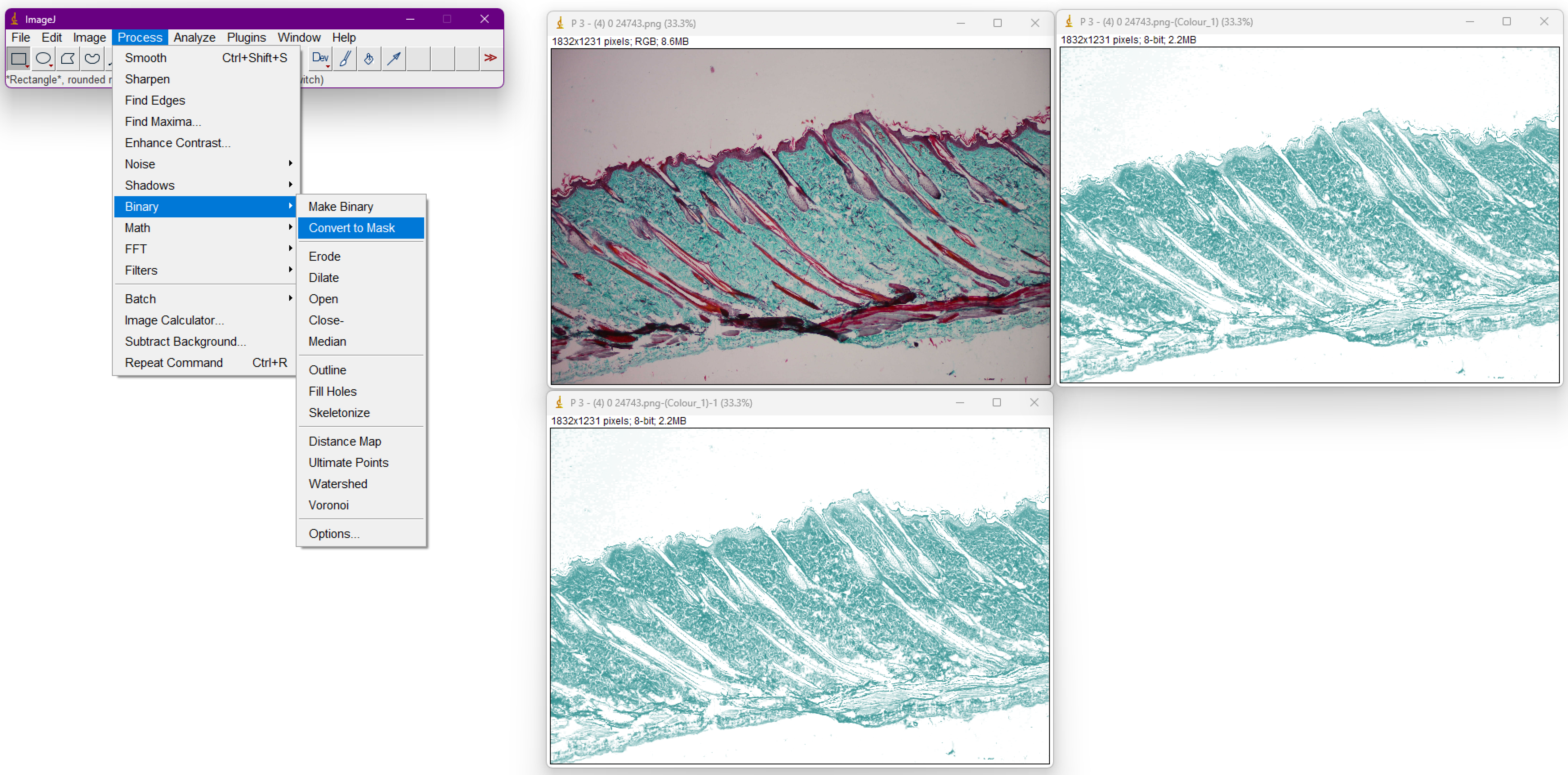
ImageJ
File Edit Image Process Analyze Plugins Window Help
Rectangle, rounded r
itch)
Smooth Ctrl+Shift+S
Sharpen
Find Edges
Find Maxima...
Enhance Contrast...
Noise
Shadows
Binary
Math
FFT
Filters
Batch
Image Calculator...
Subtract Background...
Repeat Command Ctrl+R
Make Binary
Convert to Mask
Erode
Dilate
Open
Close-
Median
Outline
Fill Holes
Skeletonize
Distance Map
Ultimate Points
Watershed
Voronoi
Options...
P 3 - (4) 0 24743.png (33.3%)
1832x1231 pixels; RGB; 8.6MB
P 3 - (4) 0 24743.png-(Colour_1) (33.3%)
1832x1231 pixels; 8-bit; 2.2MB
P 3 - (4) 0 24743.png-(Colour_1)-1 (33.3%)
1832x1231 pixels; 8-bit; 2.2MB

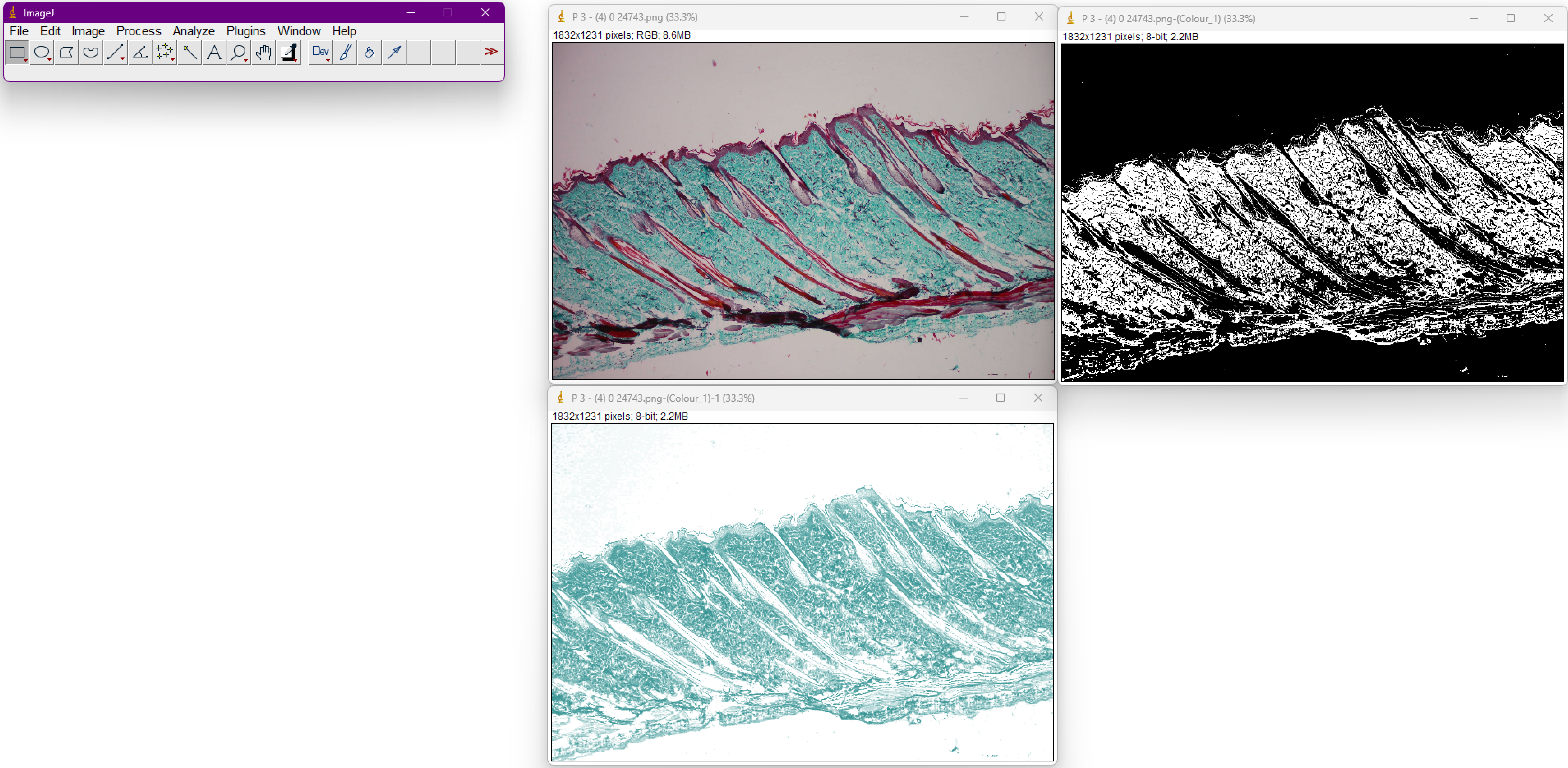
ImageJ
File Edit Image Process Analyze Plugins Window Help
P 3 - (4) 0 24743.png (33.3%)
1832x1231 pixels; RGB; 8.6MB
P 3 - (4) 0 24743.png-(Colour_1) (33.3%)
1832x1231 pixels; 8-bit; 2.2MB
P 3 - (4) 0 24743.png-(Colour_1)-1 (33.3%)
1832x1231 pixels; 8-bit; 2.2MB

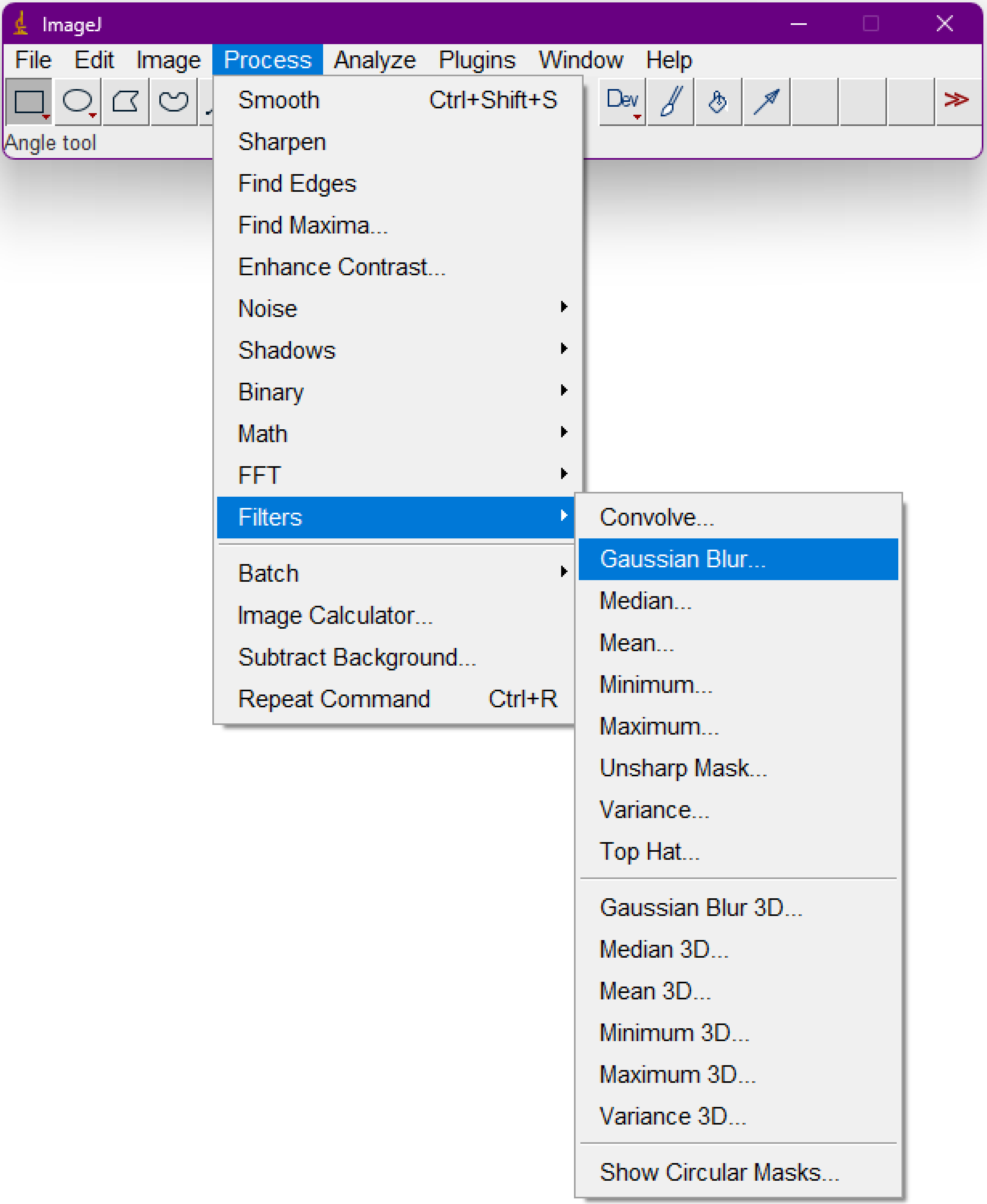
ImageJ
File
Edit
Image
Process
Analyze
Plugins
Window
Help
Dev
Angle tool
Smooth Ctrl+Shift+S
Sharpen
Find Edges
Find Maxima...
Enhance Contrast...
Noise
Shadows
Binary
Math
FFT
Filters
Batch
Image Calculator...
Subtract Background...
Repeat Command Ctrl+R
Convolve...
Gaussian Blur...
Median...
Mean...
Minimum...
Maximum...
Unsharp Mask...
Variance...
Top Hat...
Gaussian Blur 3D...
Median 3D...
Mean 3D...
Minimum 3D...
Maximum 3D...
Variance 3D...
Show Circular Masks...

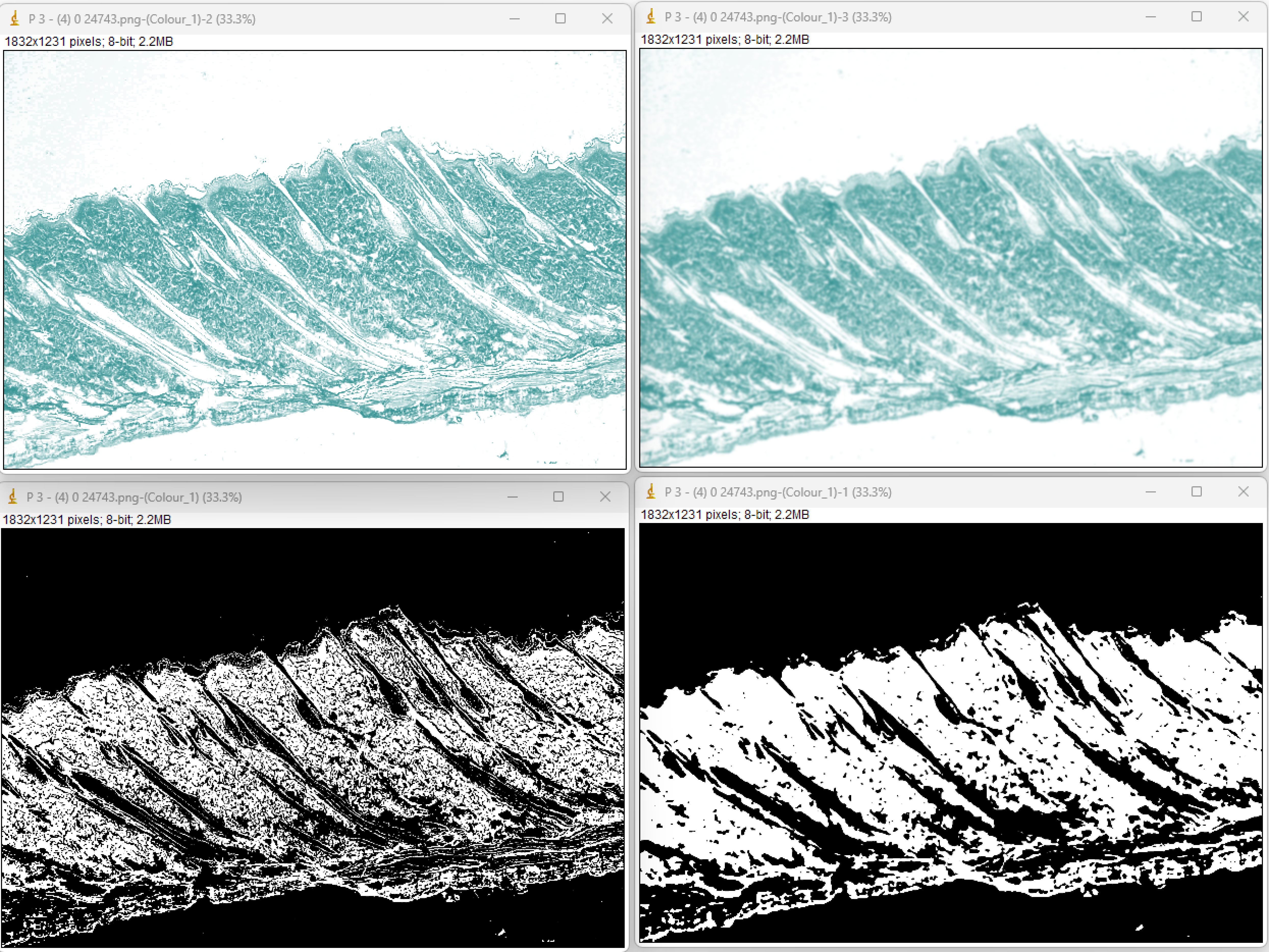
P 3 - (4) 0 24743.png-(Colour_1)-2 (33.3%)
1832x1231 pixels; 8-bit; 2.2MB
P 3 - (4) 0 24743.png-(Colour_1)-3 (33.3%)
1832x1231 pixels; 8-bit; 2.2MB
P 3 - (4) 0 24743.png-(Colour_1) (33.3%)
1832x1231 pixels; 8-bit; 2.2MB
P 3 - (4) 0 24743.png-(Colour_1)-1 (33.3%)
1832x1231 pixels; 8-bit; 2.2MB

Step 3: Isolating Stained Areas Within the Mask

After creating the dermal mask, the next step is to use it to isolate the areas that are actually stained blue-green (collagen) and red-pink (non-collagen) within the dermis.

1. **Use Image Calculator:**
    - Navigate to the menu
      Process > Image Calculator....

2. **"AND" Operation for Collagen (Blue-Green):**
    - In the Image Calculator window:
        - Image1: Select the **blue-green** deconvoluted image.
        - Operation: Select **AND**.
        - Image2: Select the **dermal mask** created in Step 2.
    - Click **OK**. The result is an image showing only the blue-green areas within the boundaries of the dermal mask. (As seen in *Screenshot Page 19*).
3. **"AND" Operation for Cytoplasm (Red-Pink):**
    - Repeat the Image Calculator process:
        - Image1: Select the **red-pink** deconvoluted image.
        - Operation: Select **AND**.
        - Image2: Select the **dermal mask**.
    - Click **OK**. The result is an image showing only the red-pink areas within the boundaries of the dermal mask. (As seen in *Screenshot Page 22*).

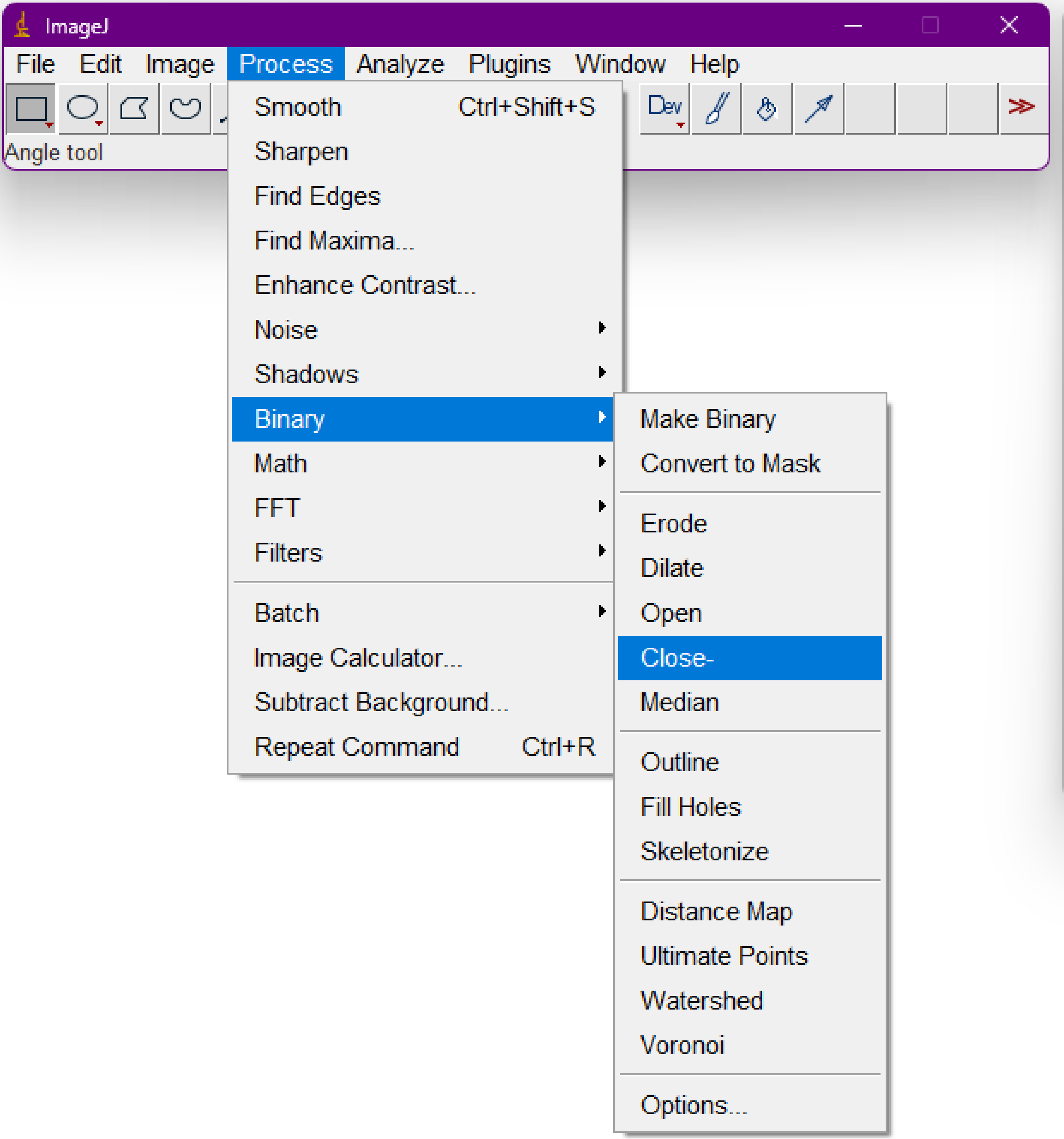
ImageJ
File
Edit
Image
Process
Analyze
Plugins
Window
Help
Angle tool
Dev
Smooth Ctrl+Shift+S
Sharpen
Find Edges
Find Maxima...
Enhance Contrast...
Noise
Shadows
Binary
Math
FFT
Filters
Batch
Image Calculator...
Subtract Background...
Repeat Command Ctrl+R
Make Binary
Convert to Mask
Erode
Dilate
Open
Close-
Median
Outline
Fill Holes
Skeletonize
Distance Map
Ultimate Points
Watershed
Voronoi
Options...

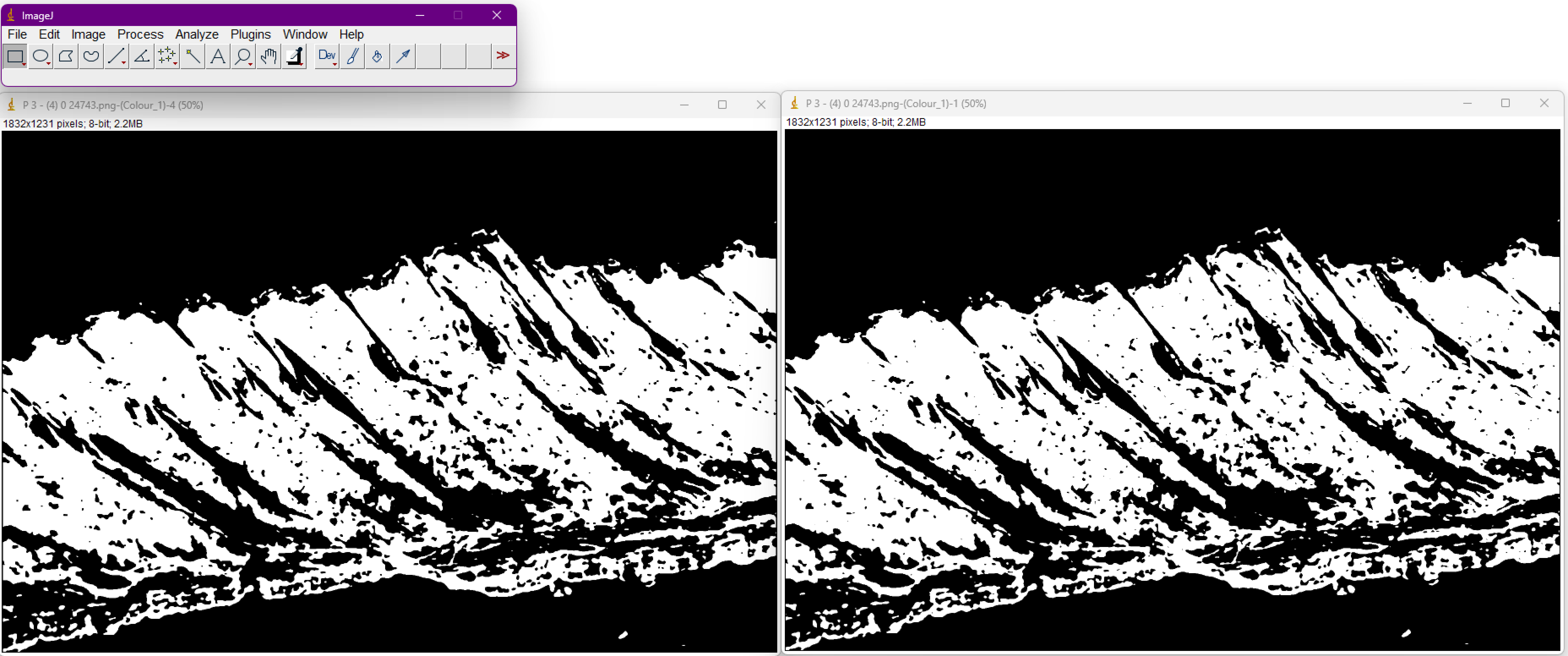
ImageJ
File Edit Image Process Analyze Plugins Window Help
Dev
P 3 - (4) 0 24743.png-(Colour_1)-4 (50%)
1832x1231 pixels; 8-bit; 2.2MB
P 3 - (4) 0 24743.png-(Colour_1)-1 (50%)
1832x1231 pixels; 8-bit; 2.2MB

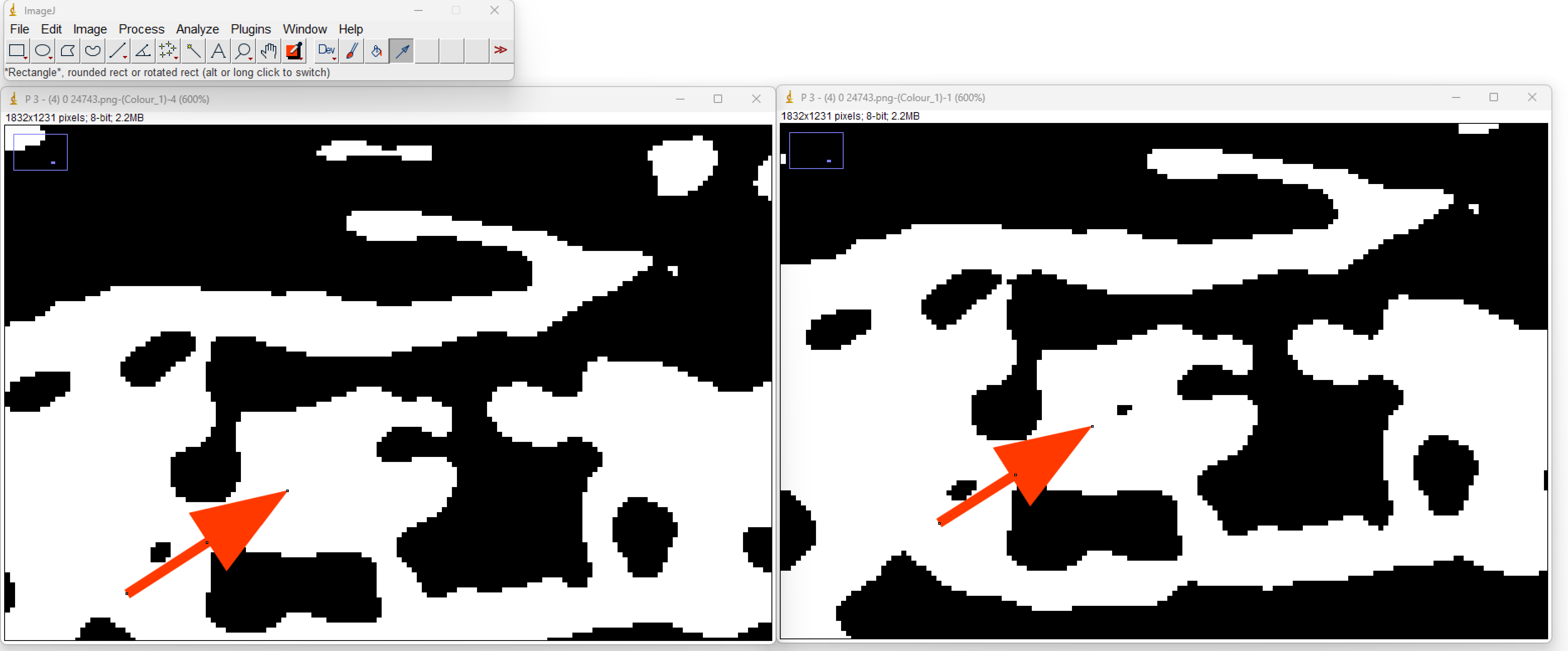
ImageJ
File Edit Image Process Analyze Plugins Window Help
Dev
Rectangle, rounded rect or rotated rect (alt or long click to switch)
P 3 - (4) 0 24743.png-(Colour_1)-4 (600%)
1832x1231 pixels; 8-bit; 2.2MB
P 3 - (4) 0 24743.png-(Colour_1)-1 (600%)
1832x1231 pixels; 8-bit; 2.2MB

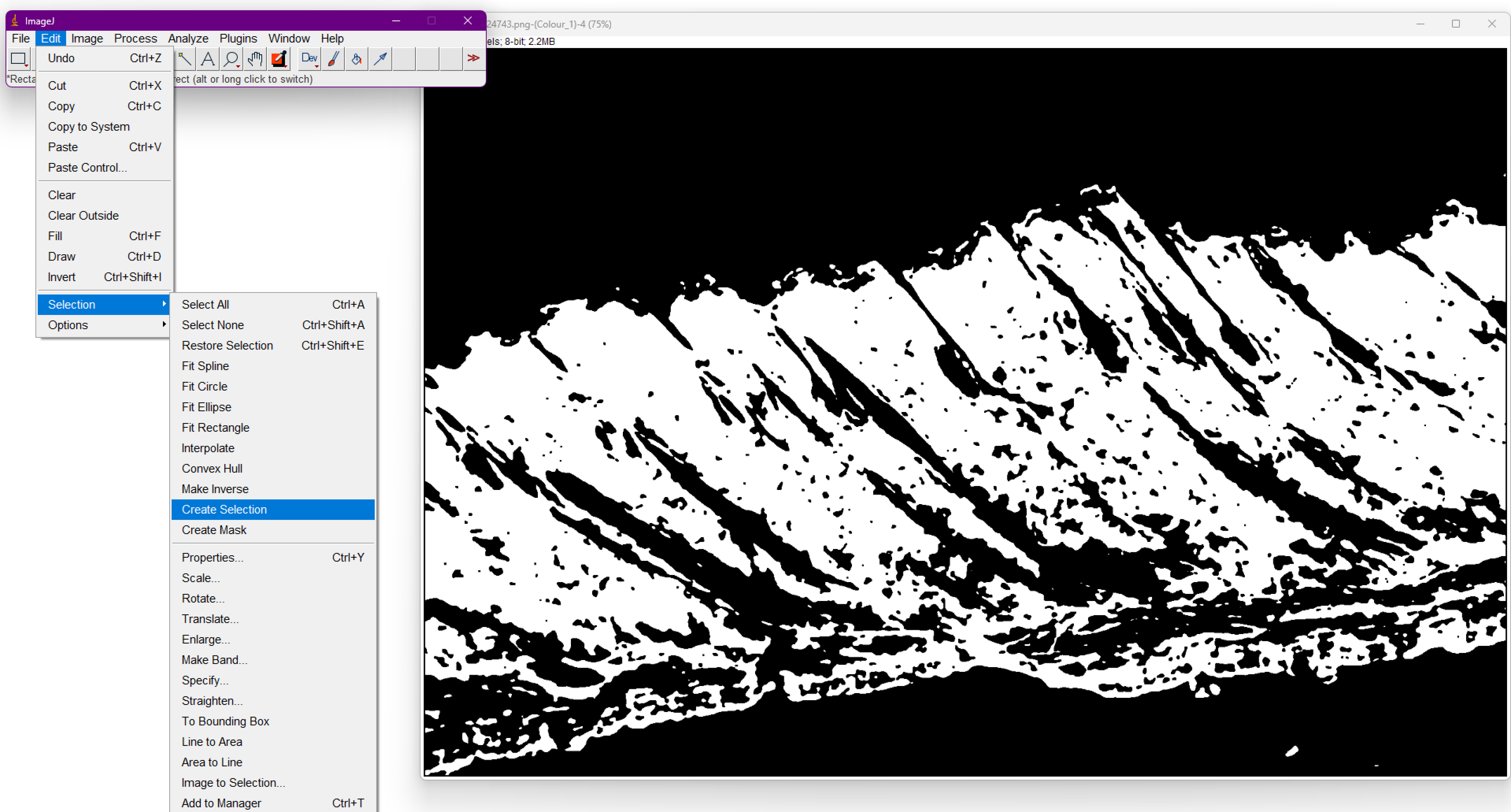
ImageJ
File Edit Image Process Analyze Plugins Window Help
*Recta
ect (alt or long click to switch)
Undo Ctrl+Z
Cut Ctrl+X
Copy Ctrl+C
Copy to System
Paste Ctrl+V
Paste Control...
Clear
Clear Outside
Fill Ctrl+F
Draw Ctrl+D
Invert Ctrl+Shift+I
Selection
Options
Select All Ctrl+A
Select None Ctrl+Shift+A
Restore Selection Ctrl+Shift+E
Fit Spline
Fit Circle
Fit Ellipse
Fit Rectangle
Interpolate
Convex Hull
Make Inverse
Create Selection
Create Mask
Properties... Ctrl+Y
Scale...
Rotate...
Translate...
Enlarge...
Make Band...
Specify...
Straighten...
To Bounding Box
Line to Area
Area to Line
Image to Selection...
Add to Manager Ctrl+T
24743.png-(Colour_1)-4 (75%)
els; 8-bit; 2.2MB

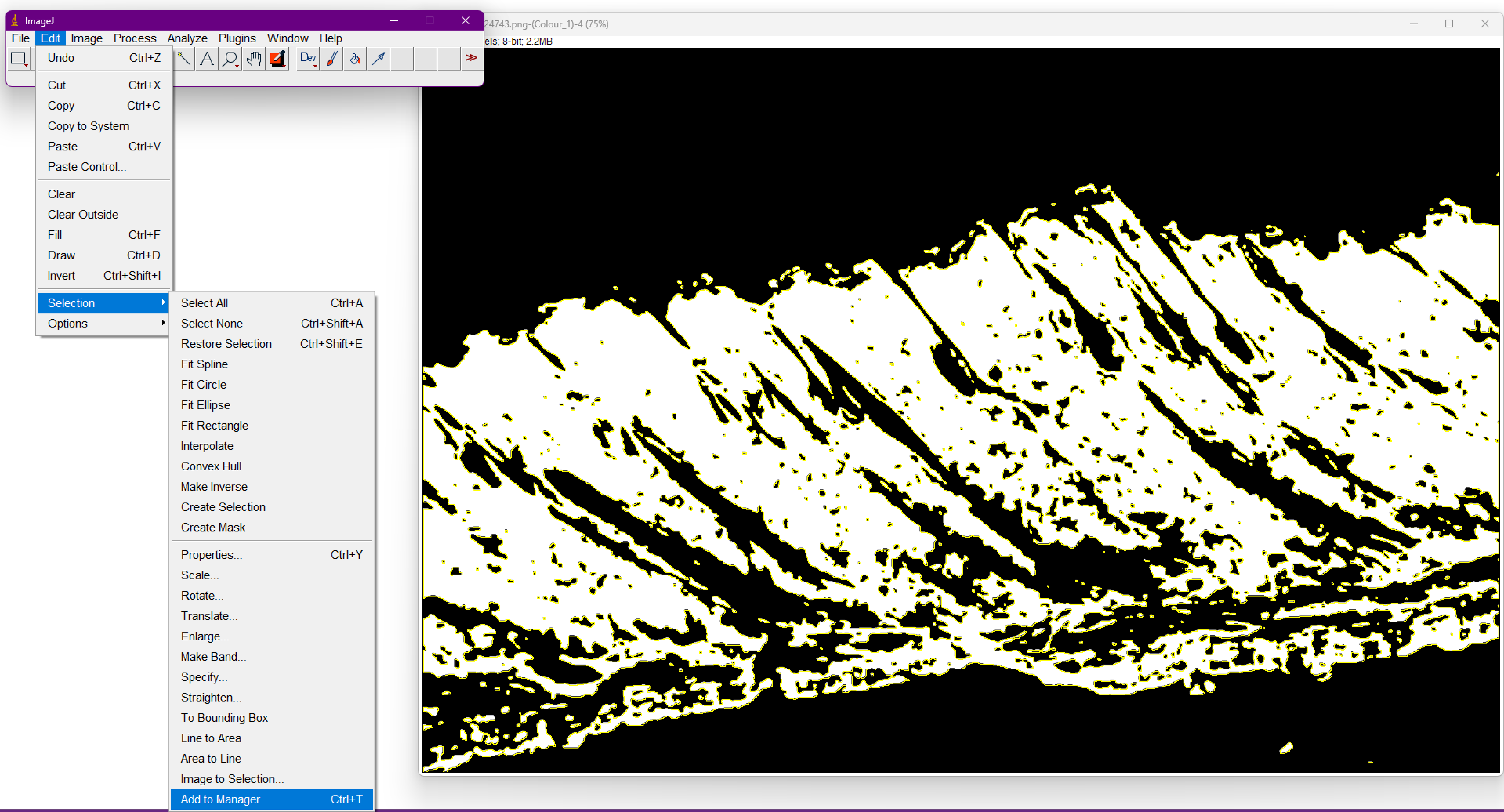
ImageJ
File
Edit
Image
Process
Analyze
Plugins
Window
Help
Dev
Undo Ctrl+Z
Cut Ctrl+X
Copy Ctrl+C
Copy to System
Paste Ctrl+V
Paste Control...
Clear
Clear Outside
Fill Ctrl+F
Draw Ctrl+D
Invert Ctrl+Shift+I
Selection
Options
Select All Ctrl+A
Select None Ctrl+Shift+A
Restore Selection Ctrl+Shift+E
Fit Spline
Fit Circle
Fit Ellipse
Fit Rectangle
Interpolate
Convex Hull
Make Inverse
Create Selection
Create Mask
Properties... Ctrl+Y
Scale...
Rotate...
Translate...
Enlarge...
Make Band...
Specify...
Straighten...
To Bounding Box
Line to Area
Area to Line
Image to Selection...
Add to Manager Ctrl+T
24743.png-(Colour_1)-4 (75%)
els; 8-bit; 2.2MB

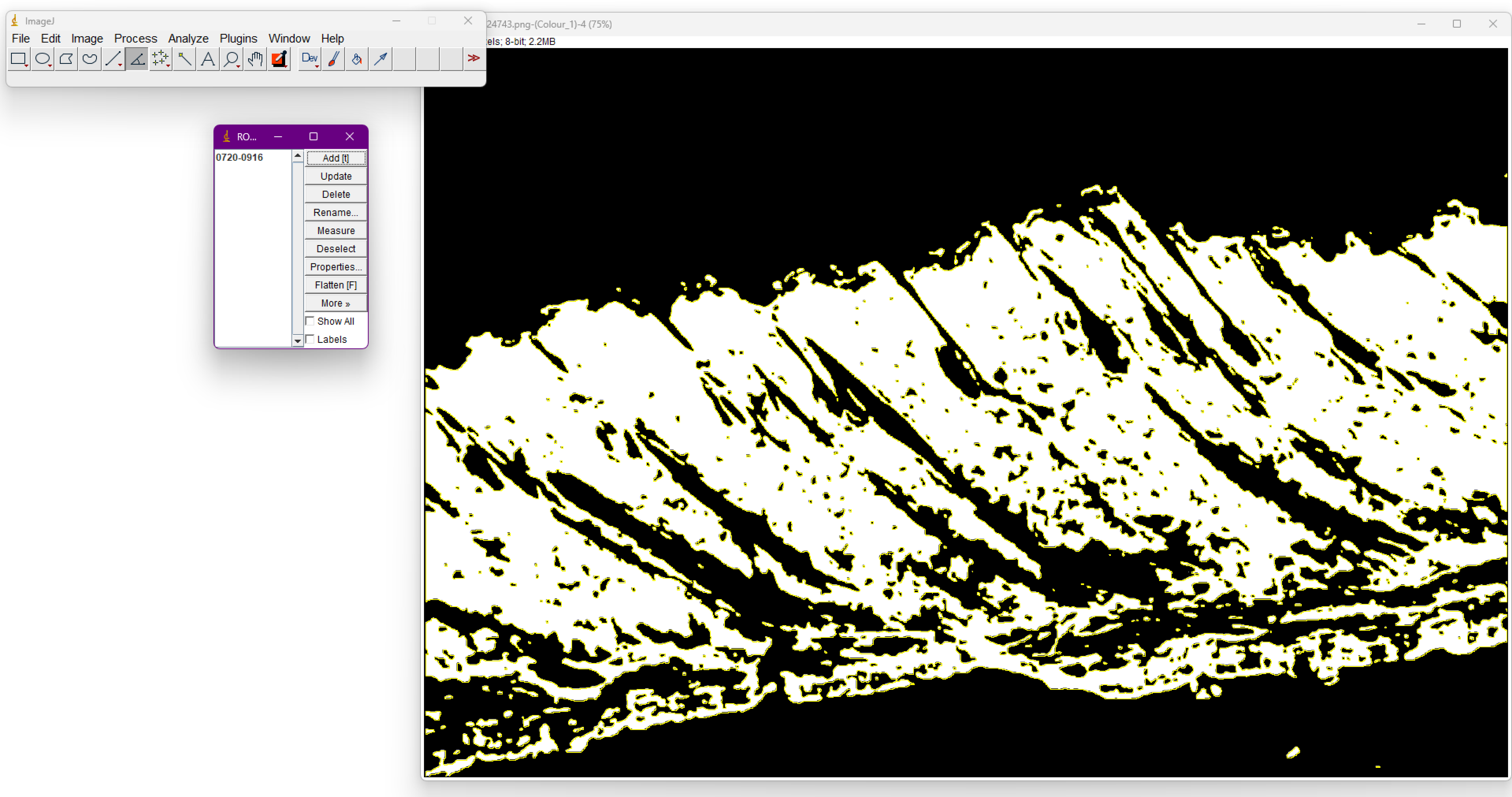
ImageJ
File Edit Image Process Analyze Plugins Window Help
Dev
24743.png-(Colour_1)-4 (75%)
els; 8-bit; 2.2MB
RO...
0720-0916
Add [t]
Update
Delete
Rename...
Measure
Deselect
Properties...
Flatten [F]
More »
Show All
Labels

Step 4: Quantitative Measurement

This is the final stage where quantitative data is extracted from the processed images.

1. **Set Measurement Parameters:**
   - Navigate to the menu
     Analyze > Set Measurements....

   - Select the parameters you wish to measure. Based on Table 2 in the article, the relevant parameters are:
     - **Area,**
     - **Mean gray value,**
     - **Min & max gray value,**
     - **Median,**
     - **Skewness,** and **Kurtosis**.

2. **Measure Collagen Area (Blue-Green):**
   - Select the window of the blue-green "AND" operation result.
   - Navigate to the menu **Analyze > Measure** (or press Ctrl+M).
   - A "Results" window will appear containing the quantitative data for the blue-green stained area. (As seen in *Screenshot Page 21*, first row).

3. **Measure Cytoplasm Area (Red-Pink):**
   - Select the window of the red-pink "AND" operation result.
   - Press Ctrl+M again.
   - The data for the red-pink area will be added as a new row in the "Results" window. (As seen in
     Screenshot Page 21, second row).

4. **Export Data:**
   - The data from the "Results" window can be saved as a .csv file for further statistical analysis in software such as SPSS, R, or Microsoft Excel.

Visualization of Step 4: The "AND" result image is measured to generate quantitative data in the "Results" window.

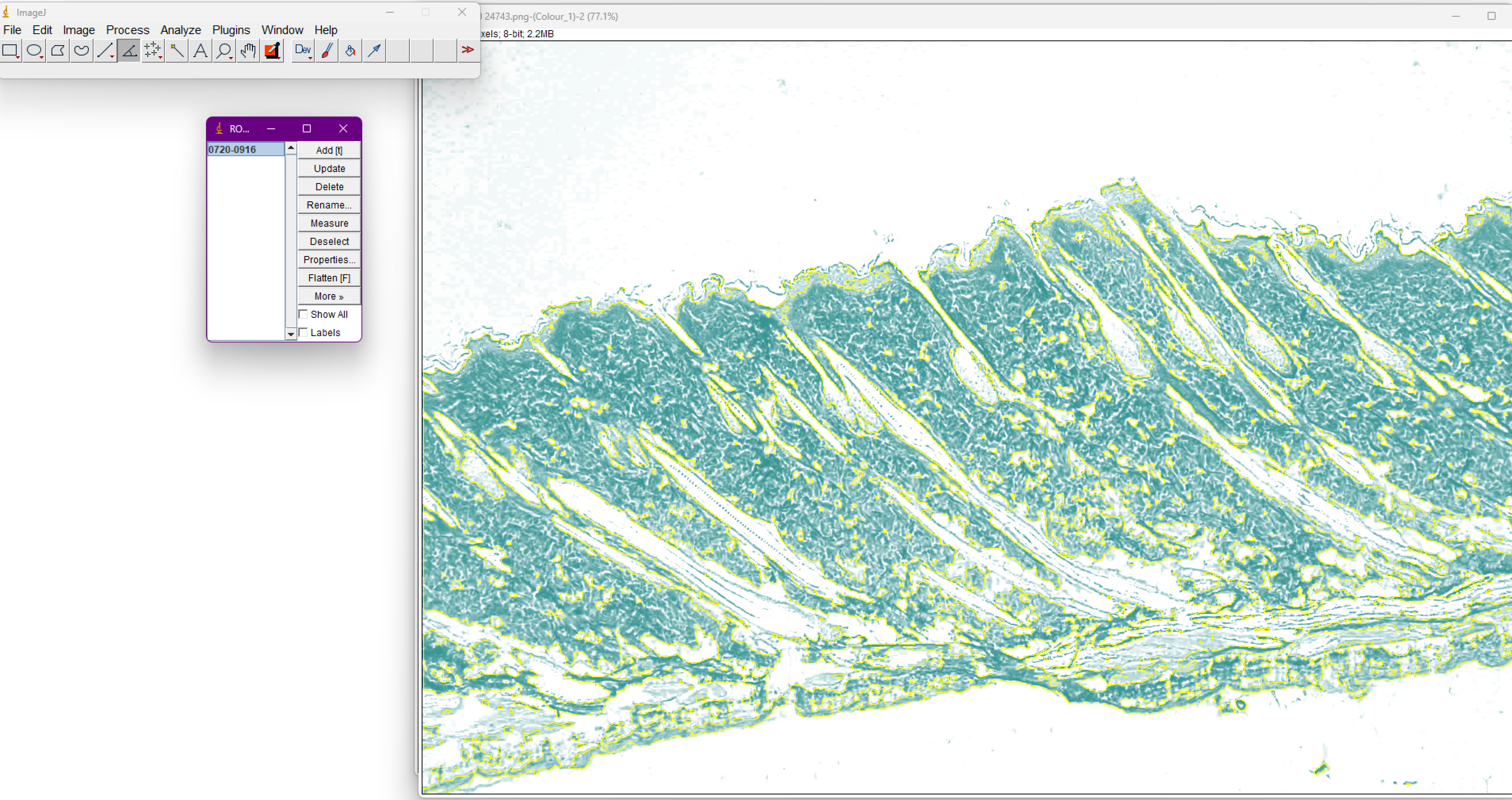
ImageJ
File Edit Image Process Analyze Plugins Window Help
Dev
24743.png-(Colour_1)-2 (77.1%)
xels; 8-bit; 2.2MB
RO...
0720-0916
Add [t]
Update
Delete
Rename...
Measure
Deselect
Properties...
Flatten [F]
More »
Show All
Labels

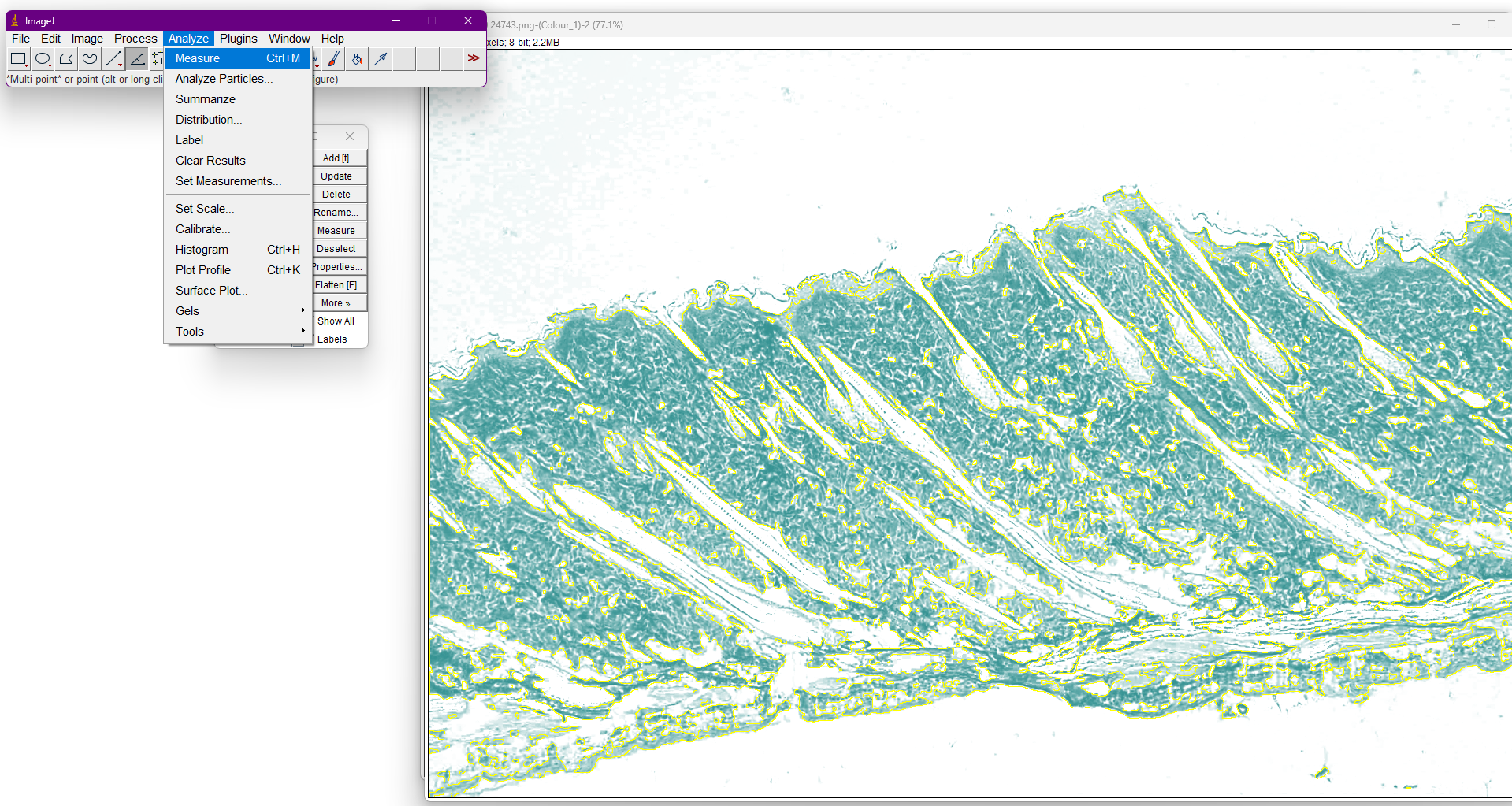
ImageJ
File
Edit
Image
Process
Analyze
Plugins
Window
Help
Multi-point or point (alt or long cli
igure)
Measure Ctrl+M
Analyze Particles...
Summarize
Distribution...
Label
Clear Results
Set Measurements...
Set Scale...
Calibrate...
Histogram Ctrl+H
Plot Profile Ctrl+K
Surface Plot...
Gels
Tools
Add [t]
Update
Delete
Rename...
Measure
Deselect
Properties...
Flatten [F]
More »
Show All
Labels
24743.png-(Colour_1)-2 (77.1%)
xels; 8-bit; 2.2MB

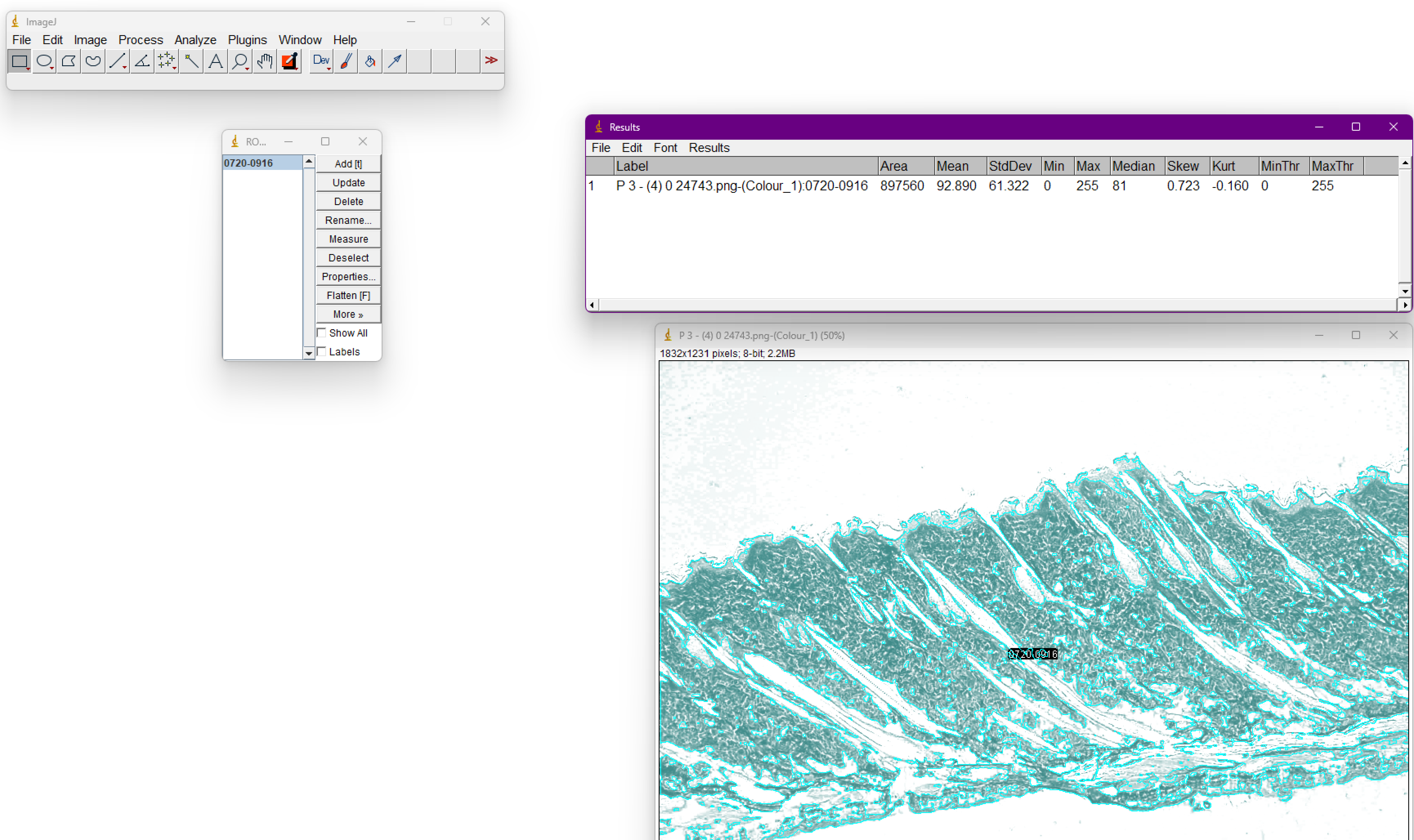
ImageJ
File Edit Image Process Analyze Plugins Window Help
Dev
RO...
0720-0916
Add [t]
Update
Delete
Rename...
Measure
Deselect
Properties...
Flatten [F]
More »
Show All
Labels
Results
File Edit Font Results
Label Area Mean StdDev Min Max Median Skew Kurt MinThr MaxThr
1 P 3 - (4) 0 24743.png-(Colour_1):0720-0916 897560 92.890 61.322 0 255 81 0.723 -0.160 0 255
P 3 - (4) 0 24743.png-(Colour_1) (50%)
1832x1231 pixels; 8-bit; 2.2MB
0720-0916

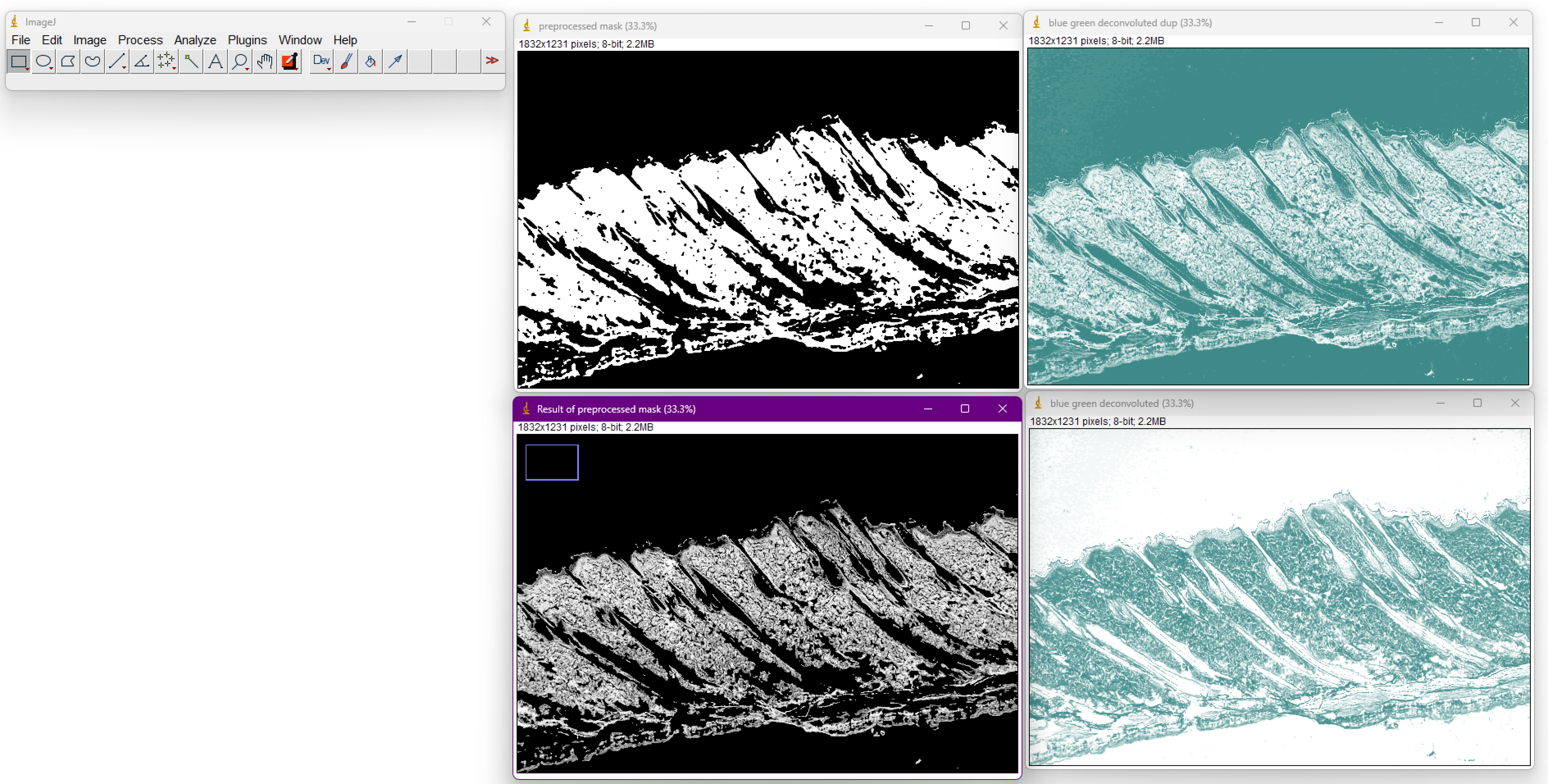
ImageJ
File Edit Image Process Analyze Plugins Window Help
Dev
preprocessed mask (33.3%)
1832x1231 pixels; 8-bit; 2.2MB
blue green deconvoluted dup (33.3%)
1832x1231 pixels; 8-bit; 2.2MB
Result of preprocessed mask (33.3%)
1832x1231 pixels; 8-bit; 2.2MB
blue green deconvoluted (33.3%)
1832x1231 pixels; 8-bit; 2.2MB

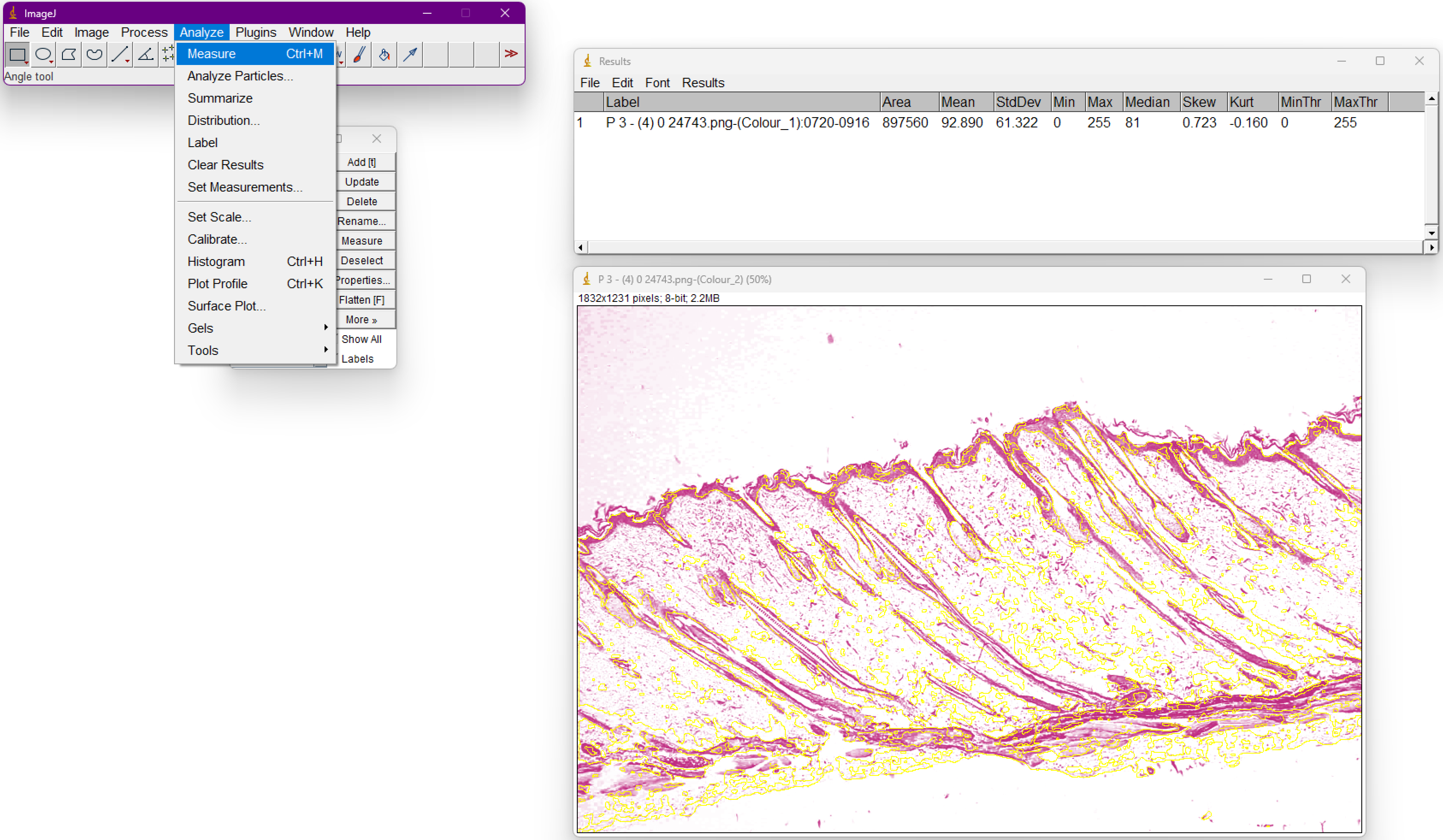
ImageJ
File Edit Image Process Analyze Plugins Window Help
Angle tool
Measure Ctrl+M
Analyze Particles...
Summarize
Distribution...
Label
Clear Results
Set Measurements...
Set Scale...
Calibrate...
Histogram Ctrl+H
Plot Profile Ctrl+K
Surface Plot...
Gels
Tools
Add [t]
Update
Delete
Rename...
Measure
Deselect
Properties...
Flatten [F]
More »
Show All
Labels
Results
File Edit Font Results
Label Area Mean StdDev Min Max Median Skew Kurt MinThr MaxThr
1 P 3 - (4) 0 24743.png-(Colour_1):0720-0916 897560 92.890 61.322 0 255 81 0.723 -0.160 0 255
P 3 - (4) 0 24743.png-(Colour_2) (50%)
1832x1231 pixels; 8-bit; 2.2MB

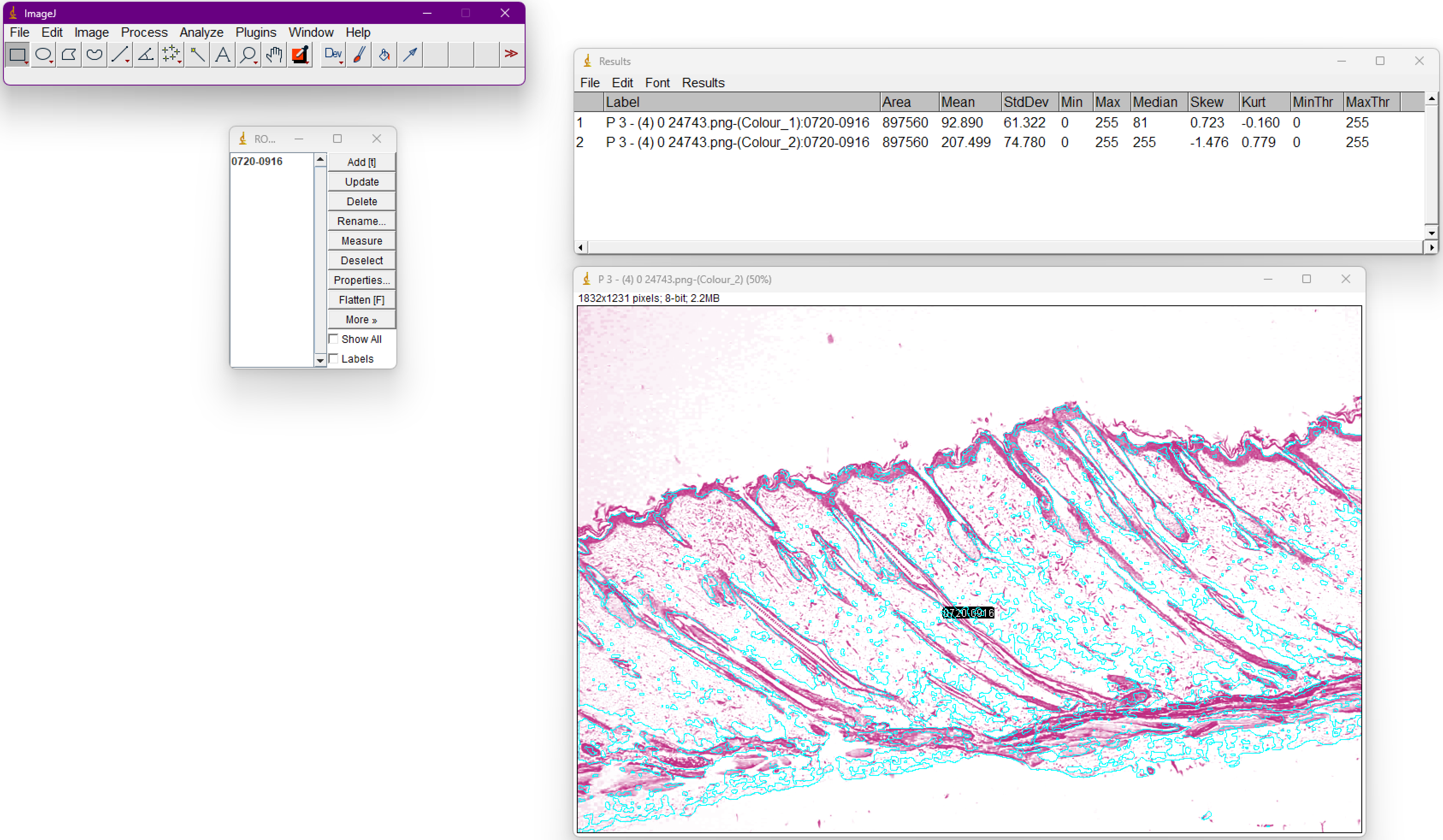
ImageJ
File Edit Image Process Analyze Plugins Window Help
Dev
RO...
0720-0916
Add [t]
Update
Delete
Rename...
Measure
Deselect
Properties...
Flatten [F]
More »
Show All
Labels
Results
File Edit Font Results
Label Area Mean StdDev Min Max Median Skew Kurt MinThr MaxThr
1 P 3 - (4) 0 24743.png-(Colour_1):0720-0916 897560 92.890 61.322 0 255 81 0.723 -0.160 0 255
2 P 3 - (4) 0 24743.png-(Colour_2):0720-0916 897560 207.499 74.780 0 255 255 -1.476 0.779 0 255
P 3 - (4) 0 24743.png-(Colour_2) (50%)
1832x1231 pixels; 8-bit; 2.2MB
0720-0916

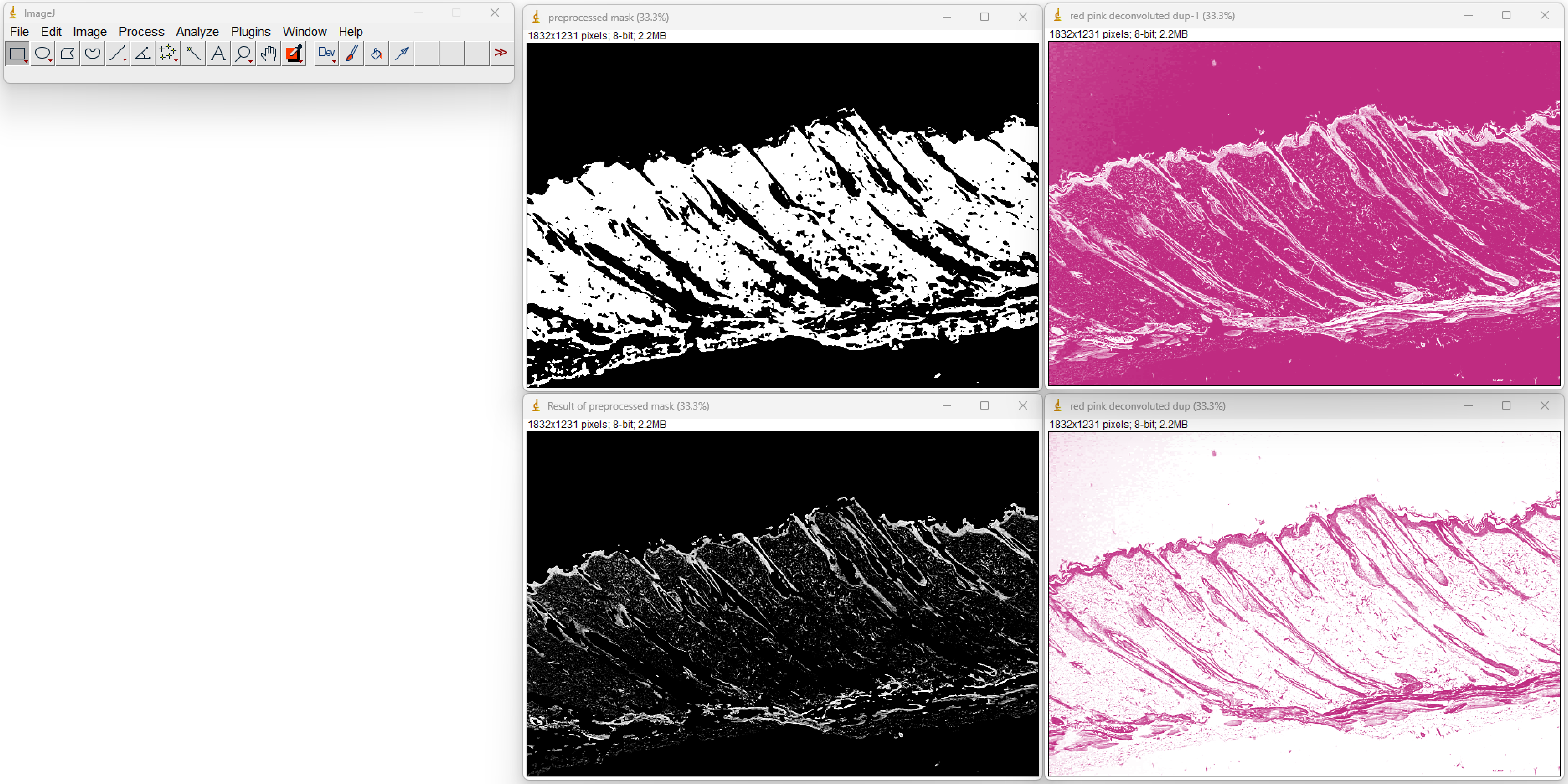
ImageJ
File Edit Image Process Analyze Plugins Window Help
Dev
preprocessed mask (33.3%)
1832x1231 pixels; 8-bit; 2.2MB
red pink deconvoluted dup-1 (33.3%)
1832x1231 pixels; 8-bit; 2.2MB
Result of preprocessed mask (33.3%)
1832x1231 pixels; 8-bit; 2.2MB
red pink deconvoluted dup (33.3%)
1832x1231 pixels; 8-bit; 2.2MB
