# Supplementary material for: Quantitative Histomorphometric Analysis of Collagen Bundles in Masson's Trichrome Stained Rat (Rattus norvegicus) Skin: A Methodological Study
Source: Health Sci Rep. 2026 Mar 8;9(3):e71998. doi: 10.1002/hsr2.71998 (PMC12967519; doi:10.1002/hsr2.71998)
Supplement: Supplementary file 3 — S2 individual histogram of stain features and normality test. [file HSR2-9-e71998-s001.pdf]

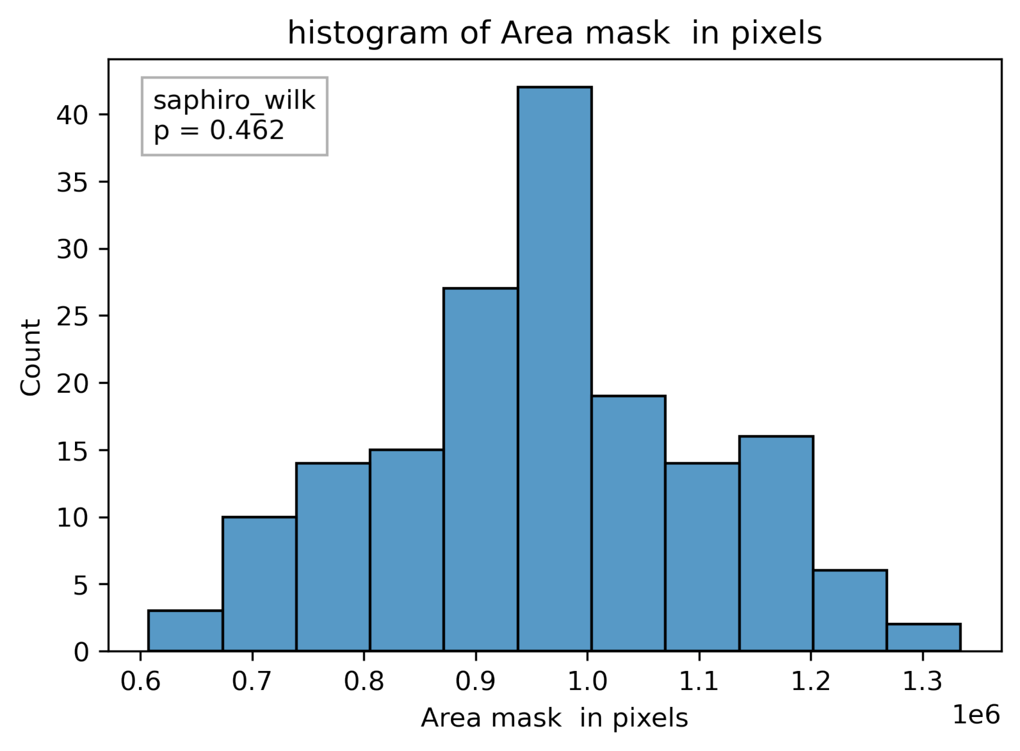
histogram of Area mask in pixels
saphiro_wilk
p = 0.462
Count
0
5
10
15
20
25
30
35
40
0.6
0.7
0.8
0.9
1.0
1.1
1.2
1.3
1e6
Area mask in pixels

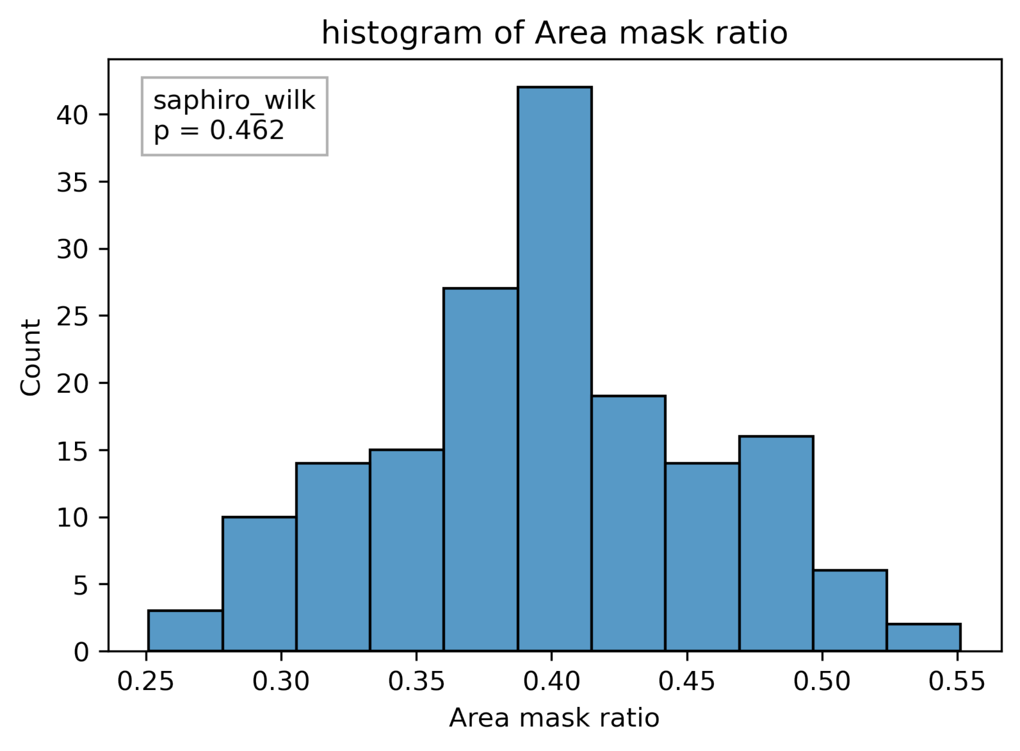

histogram of Area mask ratio
saphiro_wilk
p = 0.462
Count
Area mask ratio
0
5
10
15
20
25
30
35
40
0.25
0.30
0.35
0.40
0.45
0.50
0.55

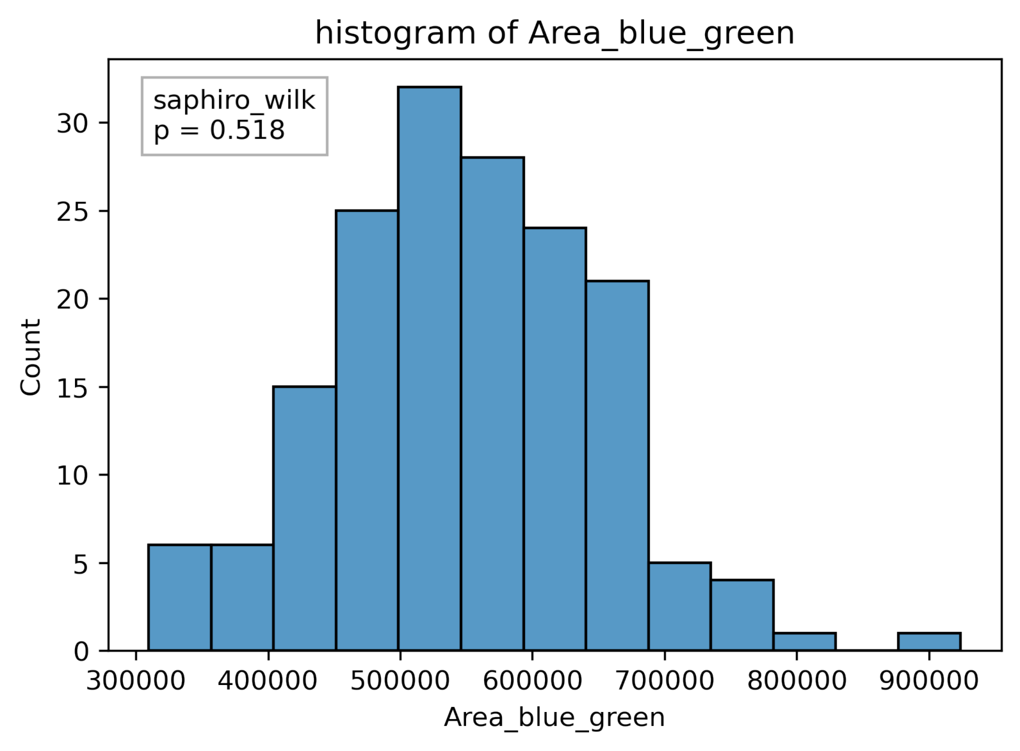
histogram of Area_blue_green
saphiro_wilk
p = 0.518
Count
0
5
10
15
20
25
30
300000
400000
500000
600000
700000
800000
900000
Area_blue_green

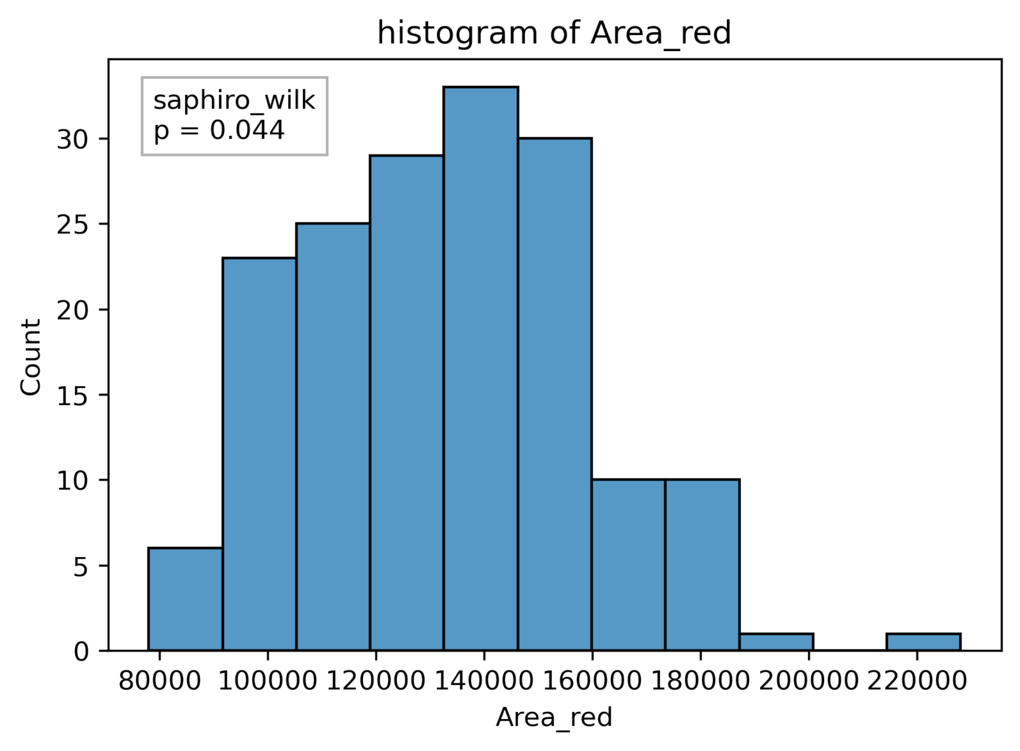

histogram of Area_red
saphiro_wilk
p = 0.044
Count
0
5
10
15
20
25
30
80000
100000
120000
140000
160000
180000
200000
220000
Area_red

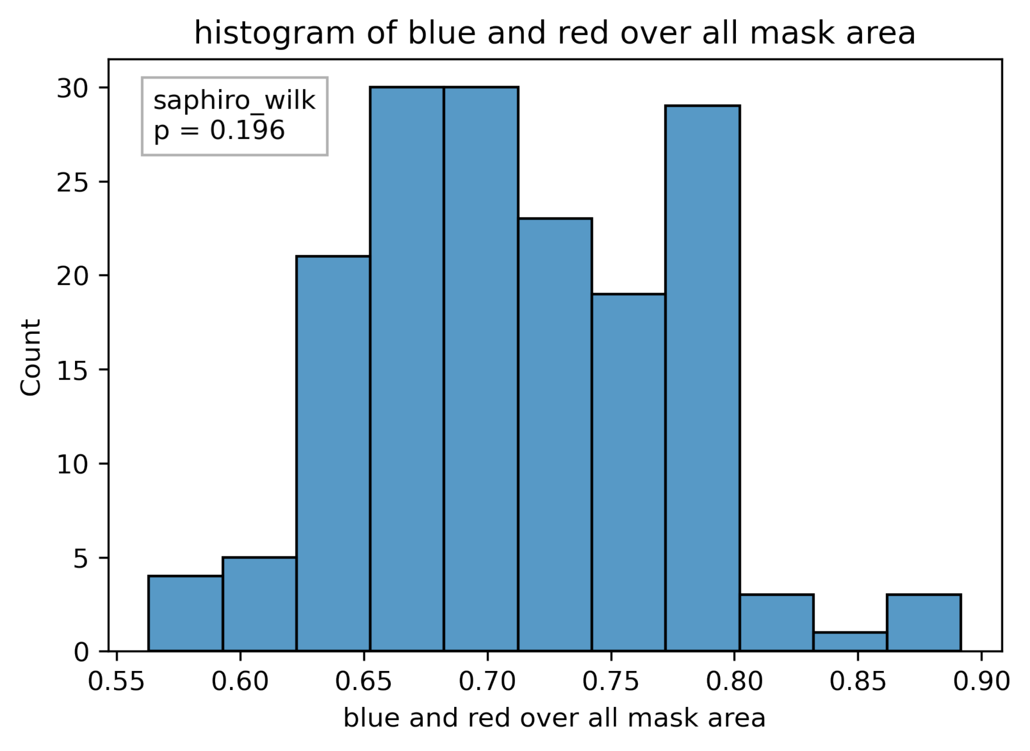

histogram of blue and red over all mask area
saphiro_wilk
p = 0.196
Count
blue and red over all mask area
0
5
10
15
20
25
30
0.55
0.60
0.65
0.70
0.75
0.80
0.85
0.90

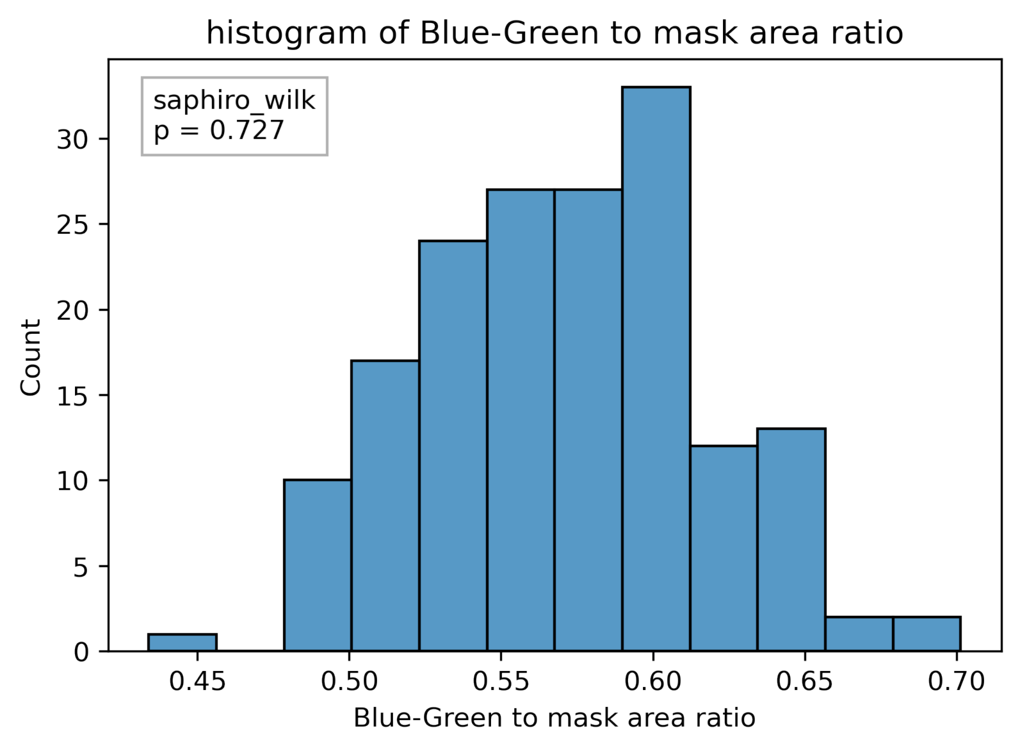
histogram of Blue-Green to mask area ratio
saphiro_wilk
p = 0.727
Count
Blue-Green to mask area ratio
0
5
10
15
20
25
30
0.45
0.50
0.55
0.60
0.65
0.70

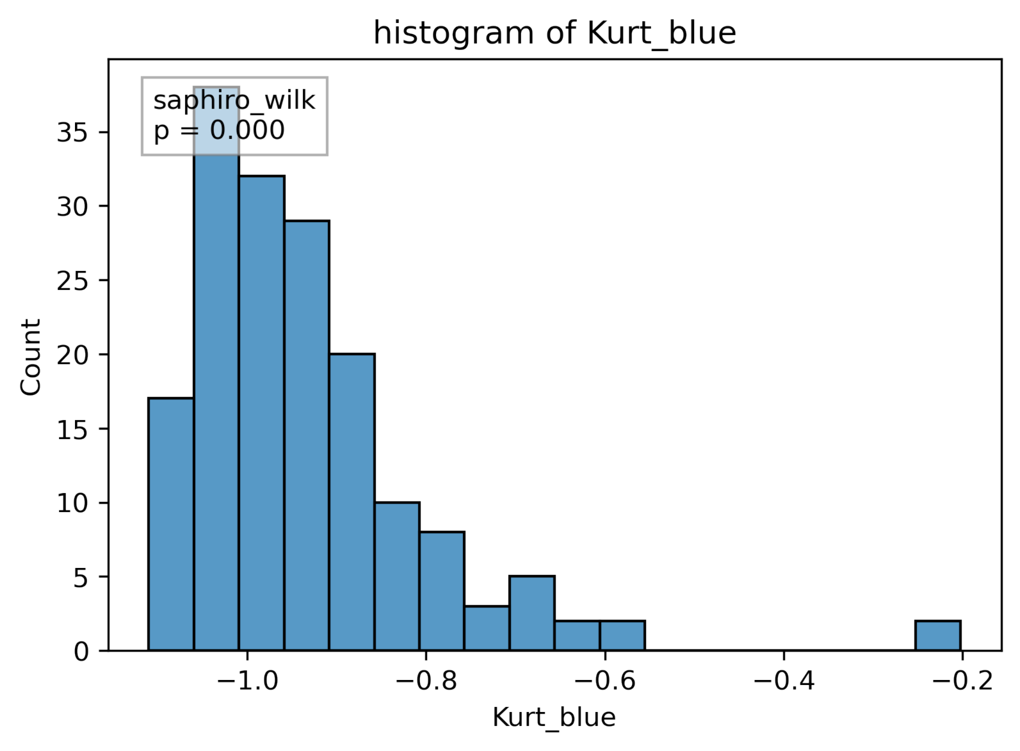
histogram of Kurt_blue
saphiro_wilk
p = 0.000
Count
35
30
25
20
15
10
5
0
−1.0
−0.8
−0.6
−0.4
−0.2
Kurt_blue

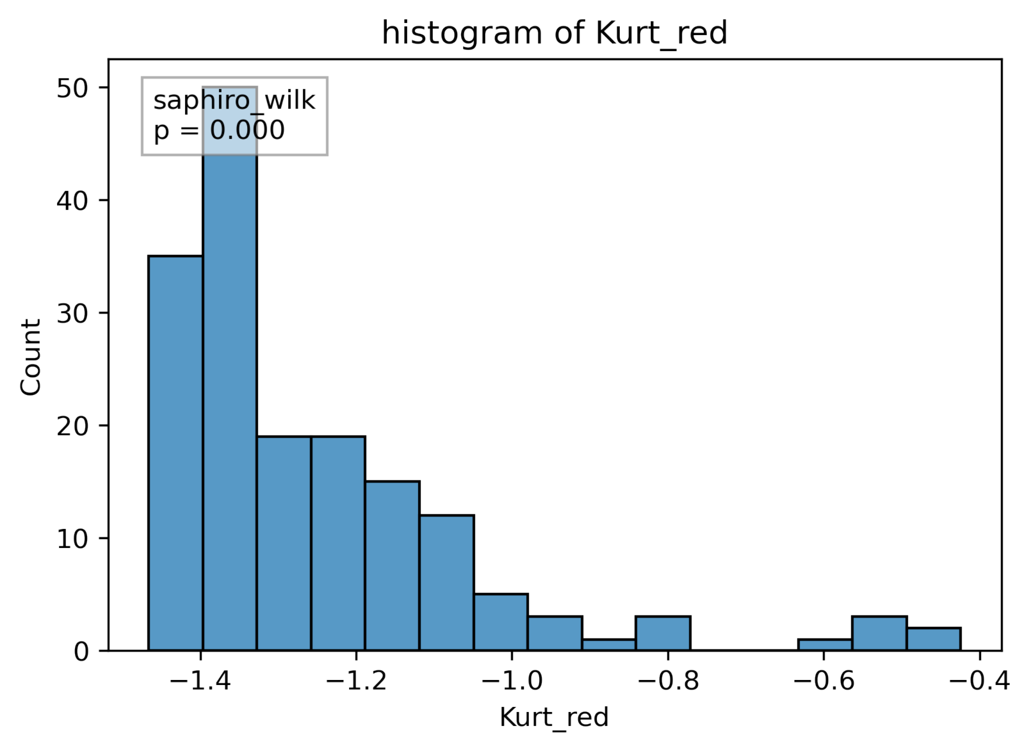
histogram of Kurt_red
saphiro_wilk
p = 0.000
Count
50
40
30
20
10
0
−1.4
−1.2
−1.0
−0.8
−0.6
−0.4
Kurt_red

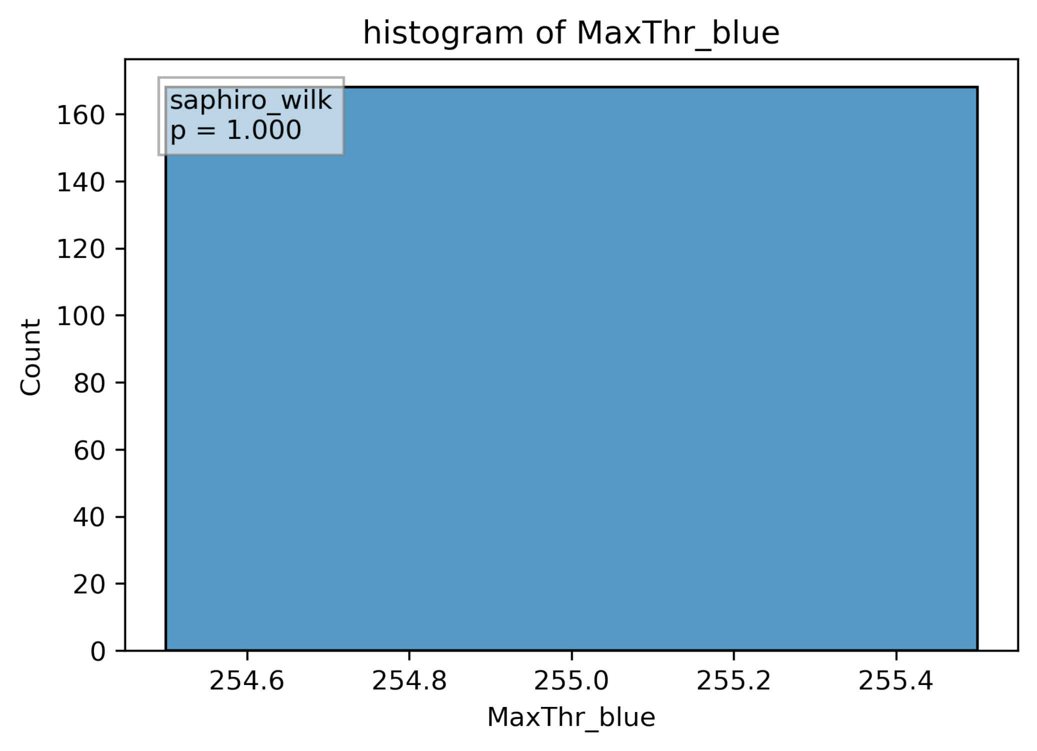
histogram of MaxThr_blue
saphiro_wilk
p = 1.000
Count
MaxThr_blue
0
20
40
60
80
100
120
140
160
254.6
254.8
255.0
255.2
255.4

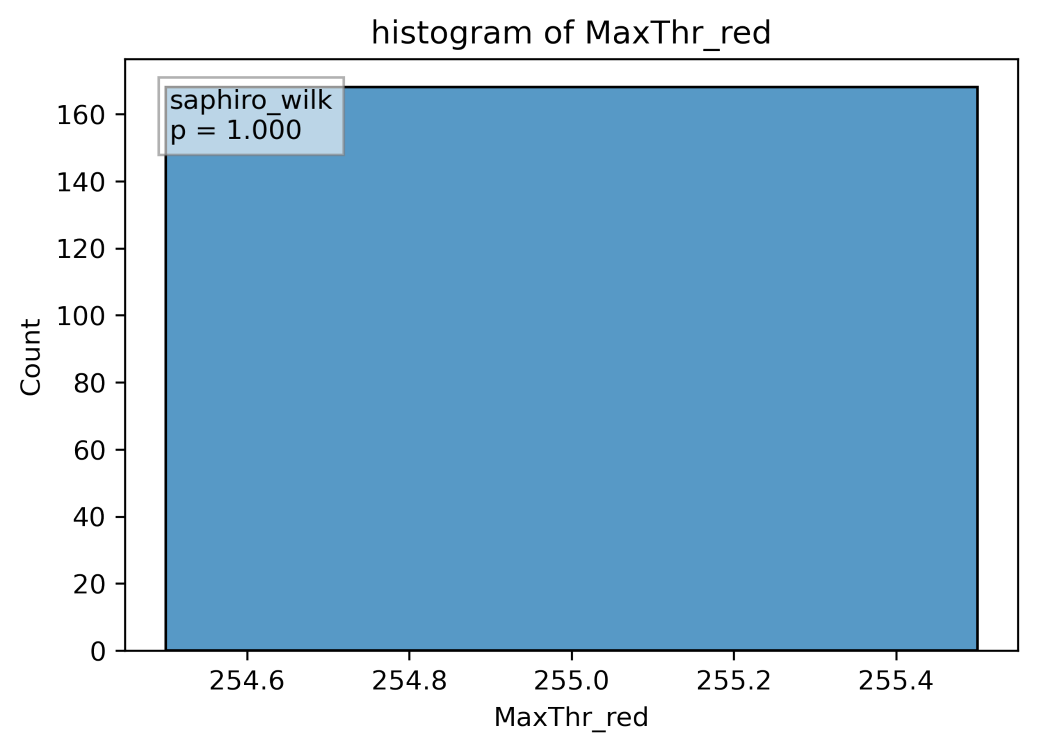
histogram of MaxThr_red
saphiro_wilk
p = 1.000
Count
0
20
40
60
80
100
120
140
160
254.6
254.8
255.0
255.2
255.4
MaxThr_red

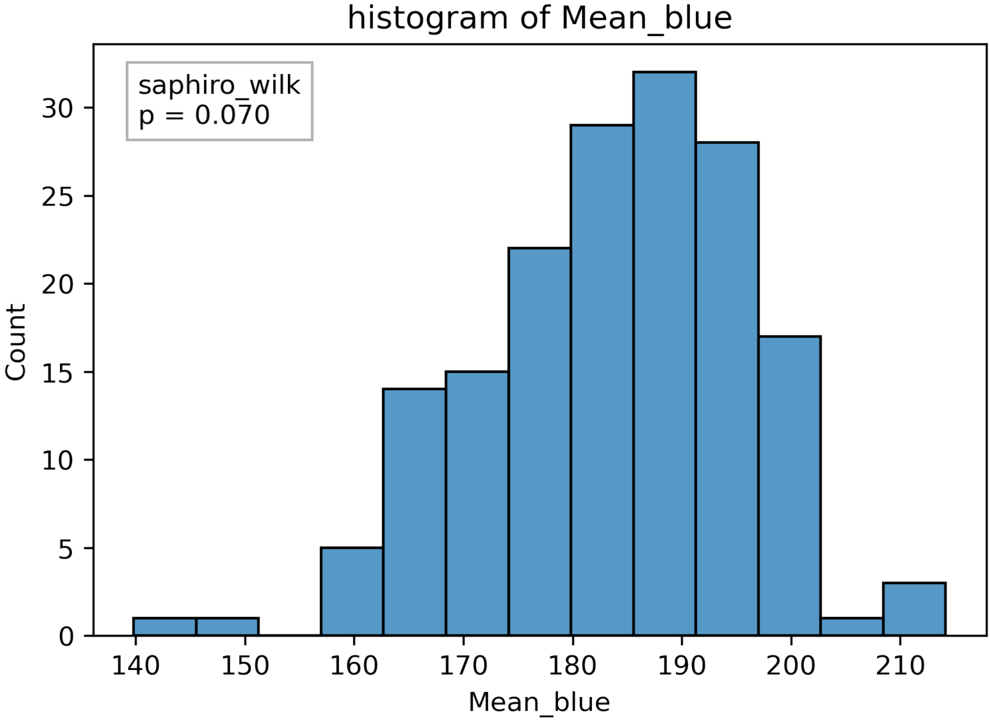
histogram of Mean_blue
saphiro_wilk
p = 0.070
Count
Mean_blue
0
5
10
15
20
25
30
140
150
160
170
180
190
200
210

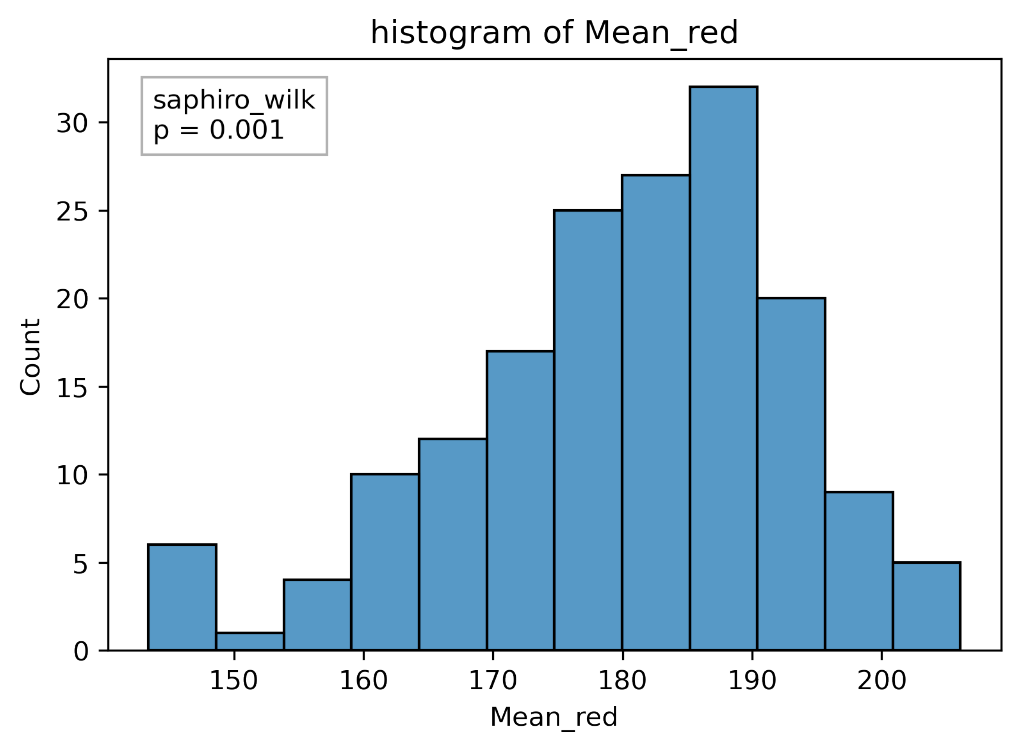
histogram of Mean_red
saphiro_wilk
p = 0.001
Count
Mean_red
0
5
10
15
20
25
30
150
160
170
180
190
200

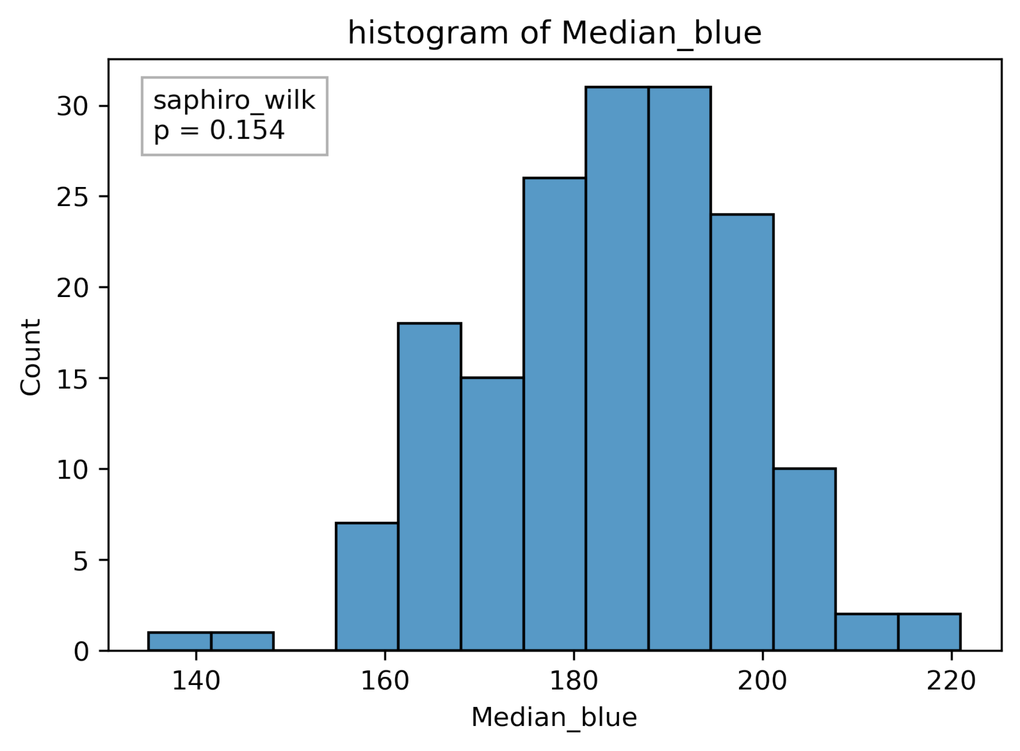
histogram of Median_blue
saphiro_wilk
p = 0.154
Count
Median_blue
0
5
10
15
20
25
30
140
160
180
200
220

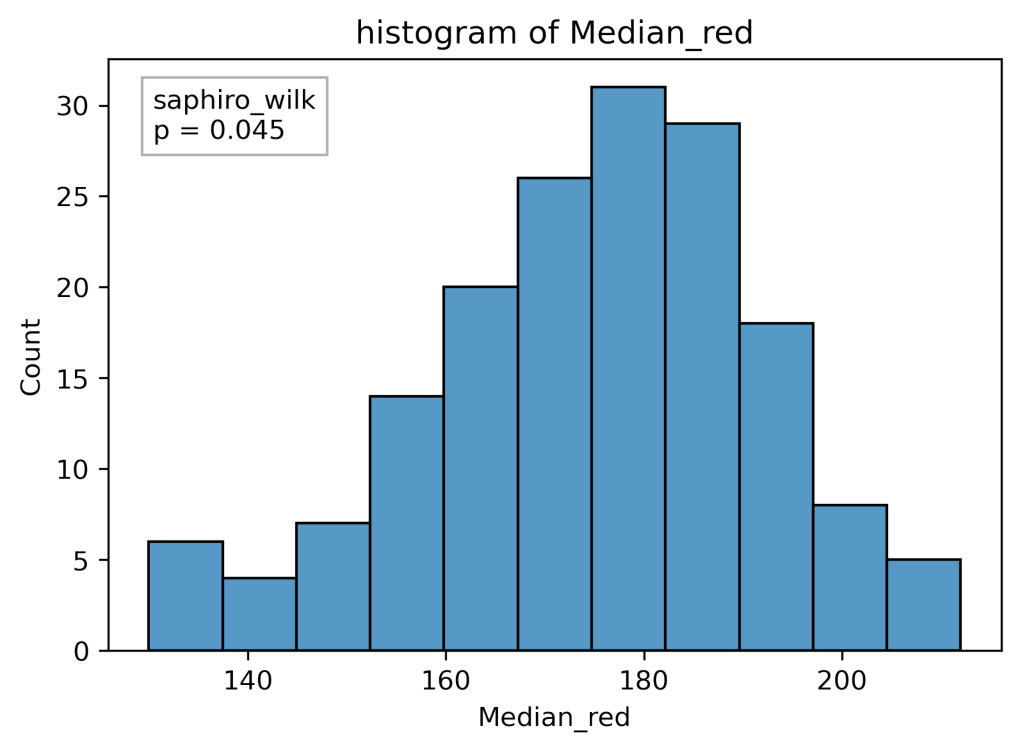
histogram of Median_red
saphiro_wilk
p = 0.045
Count
Median_red
0
5
10
15
20
25
30
140
160
180
200

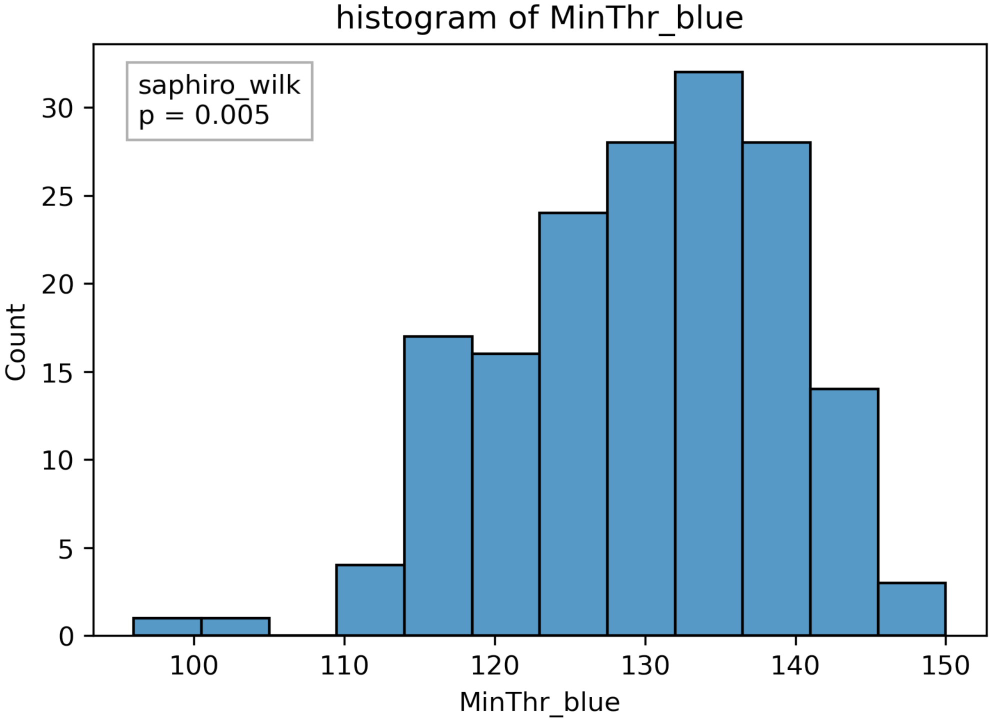
histogram of MinThr_blue
saphiro_wilk
p = 0.005
Count
MinThr_blue
0
5
10
15
20
25
30
100
110
120
130
140
150

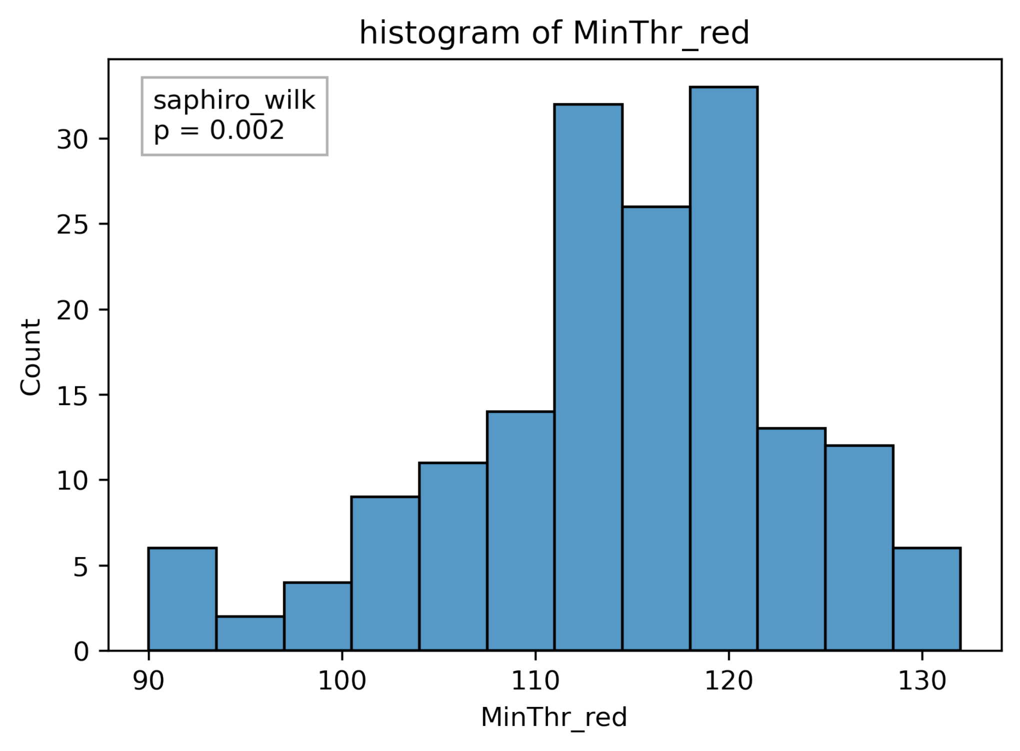
histogram of MinThr_red
saphiro_wilk
p = 0.002
Count
0
5
10
15
20
25
30
90
100
110
120
130
MinThr_red

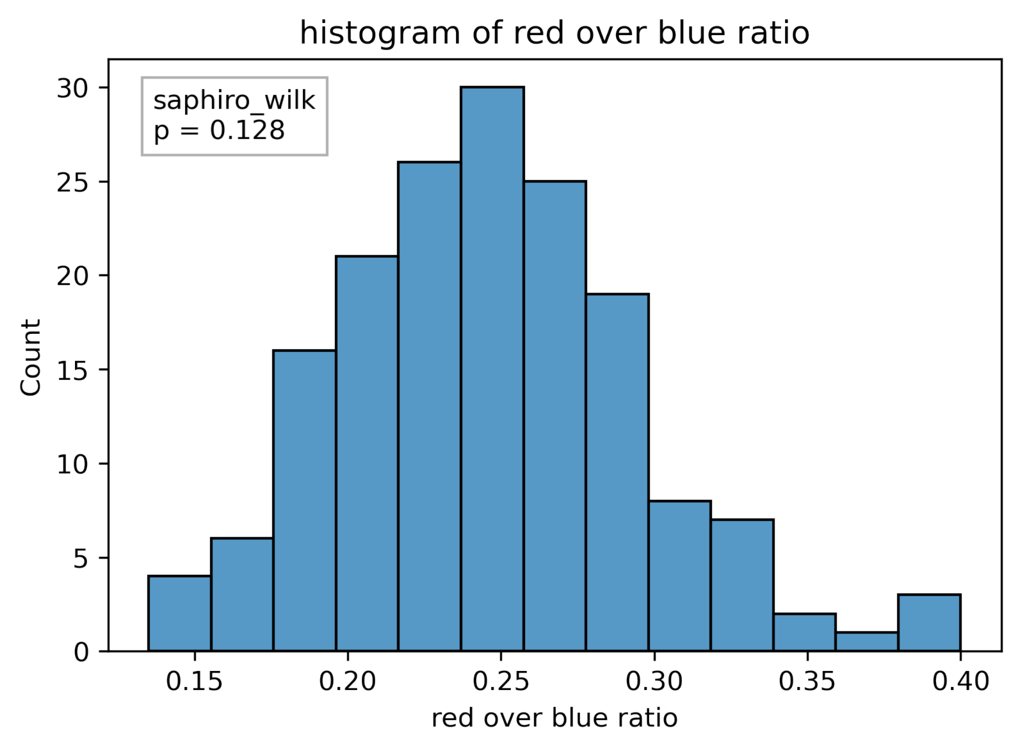
histogram of red over blue ratio
saphiro_wilk
p = 0.128
Count
30
25
20
15
10
5
0
0.15
0.20
0.25
0.30
0.35
0.40
red over blue ratio

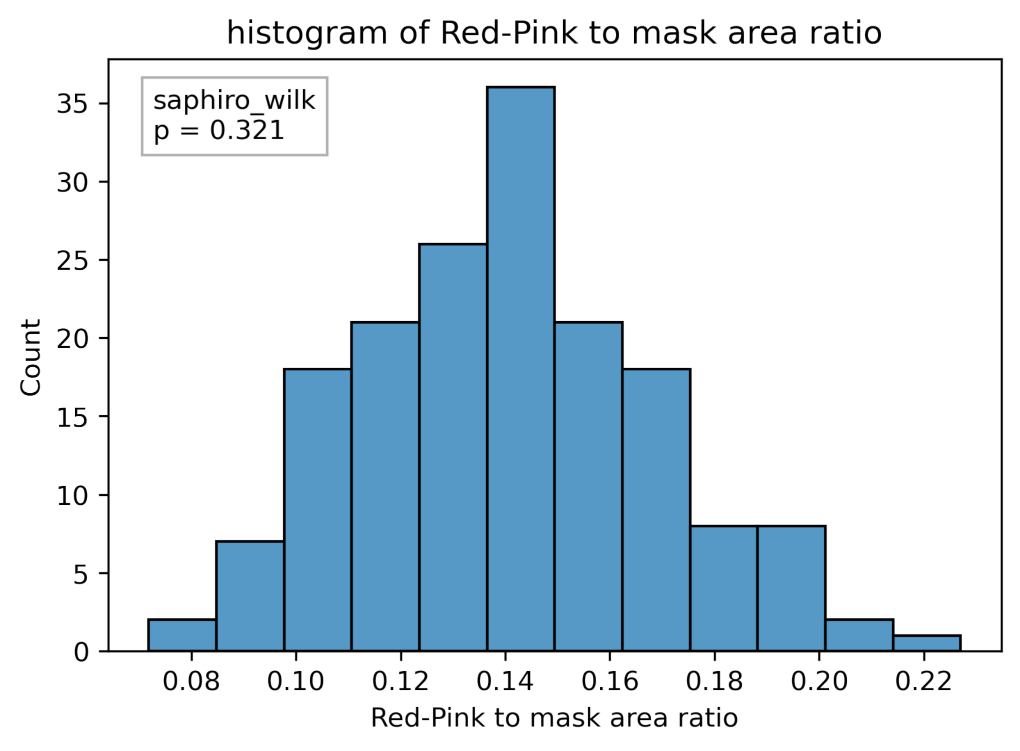
histogram of Red-Pink to mask area ratio
saphiro_wilk
p = 0.321
Count
0
5
10
15
20
25
30
35
0.08
0.10
0.12
0.14
0.16
0.18
0.20
0.22
Red-Pink to mask area ratio

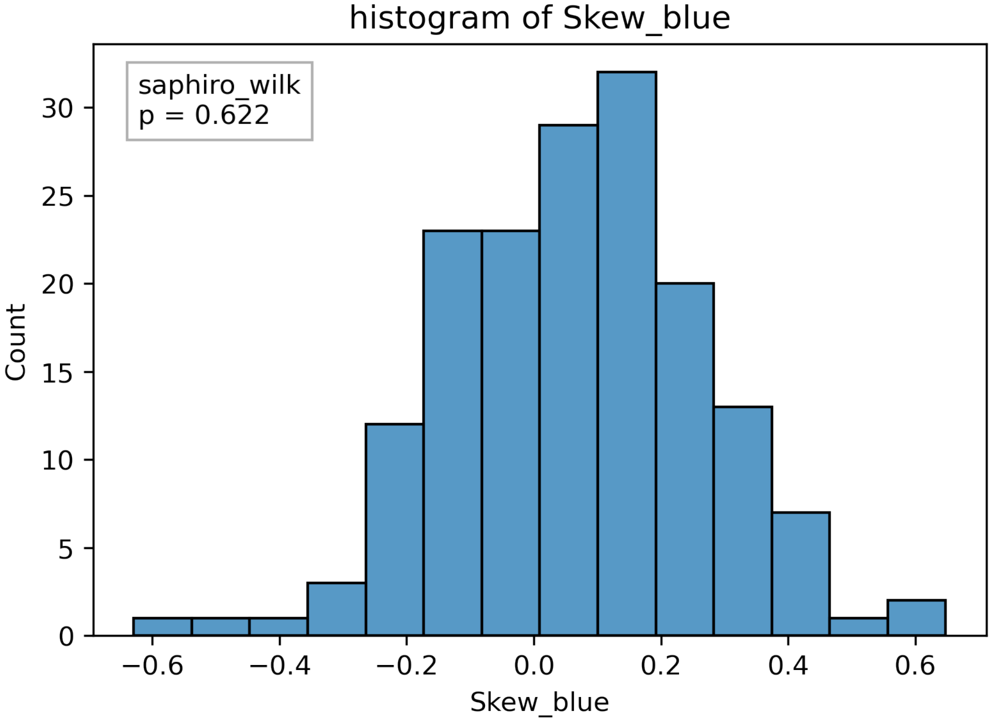
histogram of Skew_blue
saphiro_wilk
p = 0.622
Count
0
5
10
15
20
25
30
−0.6
−0.4
−0.2
0.0
0.2
0.4
0.6
Skew_blue

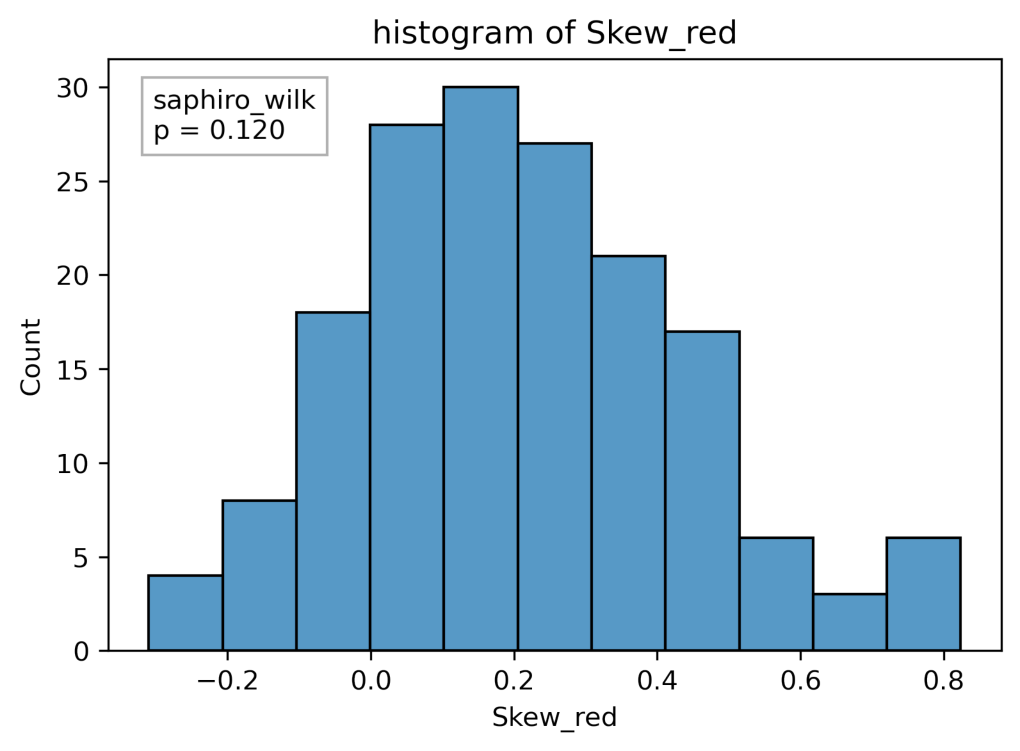

histogram of Skew_red
saphiro_wilk
p = 0.120
Count
Skew_red
30
25
20
15
10
5
0
−0.2
0.0
0.2
0.4
0.6
0.8

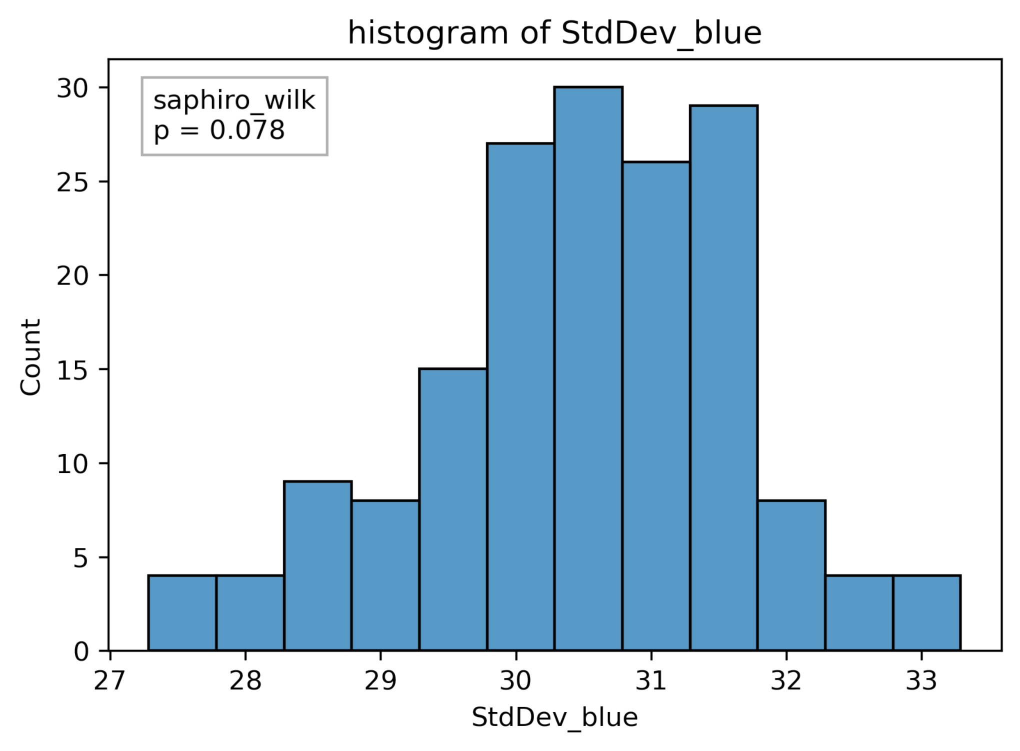
histogram of StdDev_blue
saphiro_wilk
p = 0.078
Count
StdDev_blue
0
5
10
15
20
25
30
27
28
29
30
31
32
33
