# Supplementary material for: Quantitative Histomorphometric Analysis of Collagen Bundles in Masson's Trichrome Stained Rat (Rattus norvegicus) Skin: A Methodological Study
Source: Health Sci Rep. 2026 Mar 8;9(3):e71998. doi: 10.1002/hsr2.71998 (PMC12967519; doi:10.1002/hsr2.71998)
Supplement: Supplementary file 4 — S3 comparative histogram of features between blue green and red pink stain. [file HSR2-9-e71998-s004.pdf]

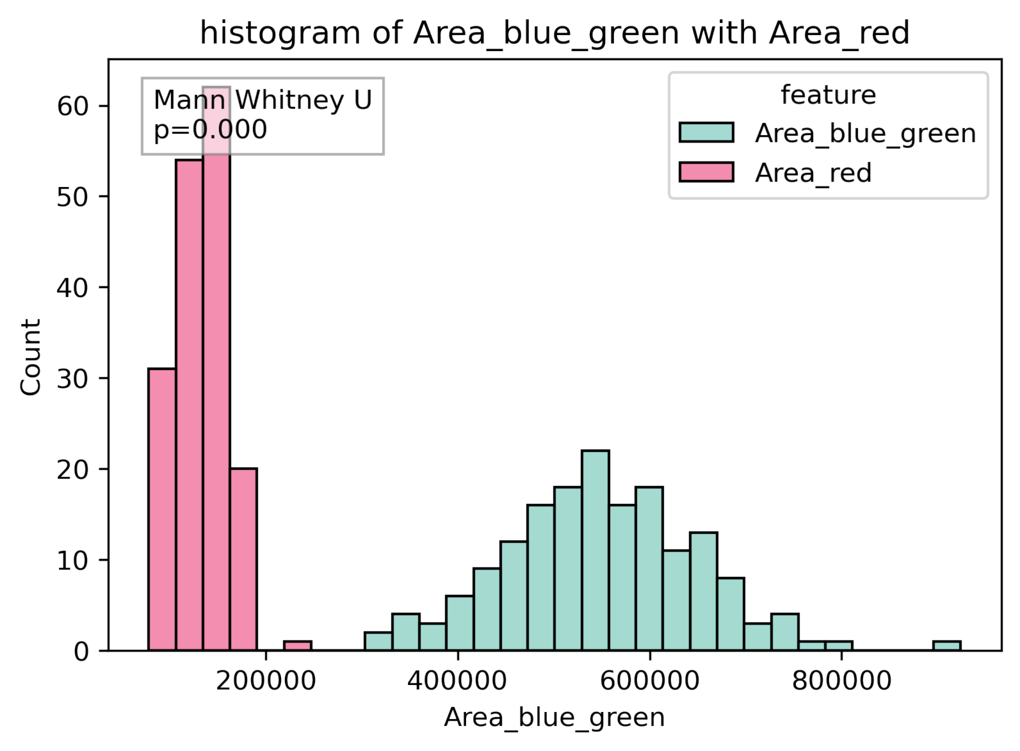
histogram of Area_blue_green with Area_red
Mann Whitney U
p=0.000
feature
Area_blue_green
Area_red
Count
0
10
20
30
40
50
60
200000
400000
600000
800000
Area_blue_green

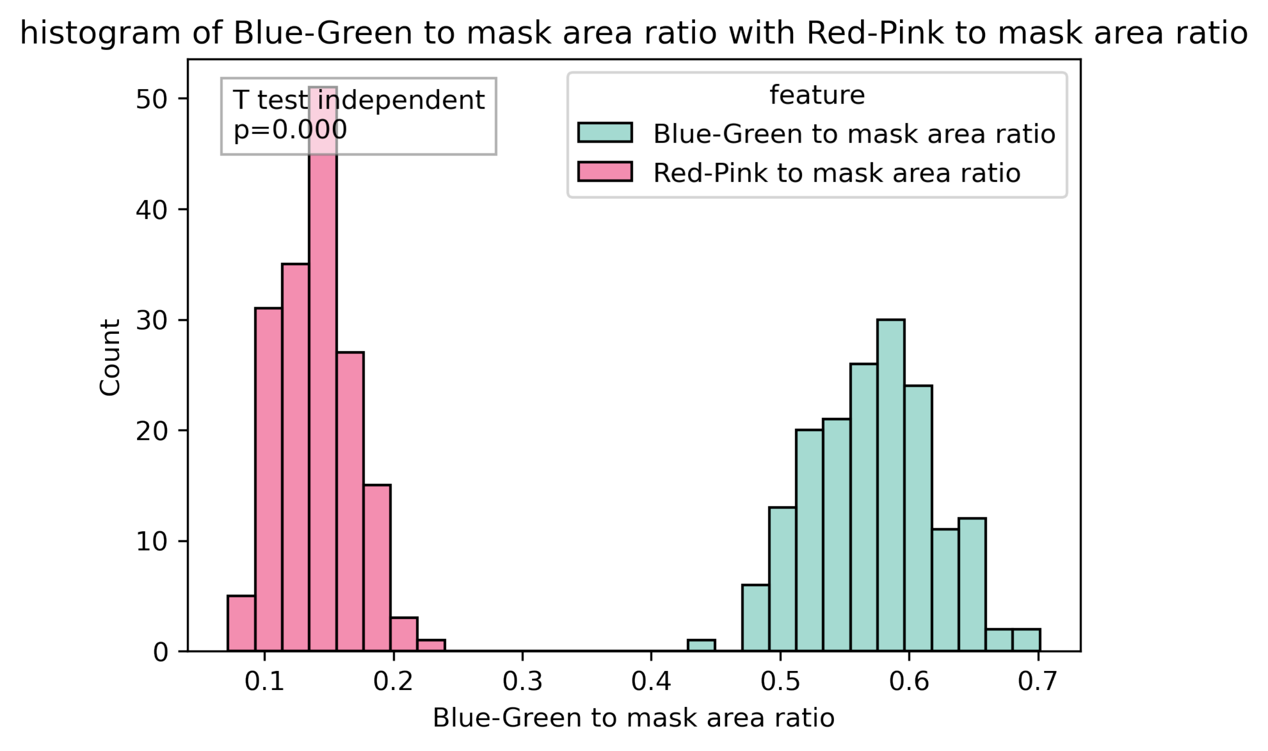
histogram of Blue-Green to mask area ratio with Red-Pink to mask area ratio
T test independent
p=0.000
feature
Blue-Green to mask area ratio
Red-Pink to mask area ratio
Count
0
10
20
30
40
50
0.1
0.2
0.3
0.4
0.5
0.6
0.7
Blue-Green to mask area ratio

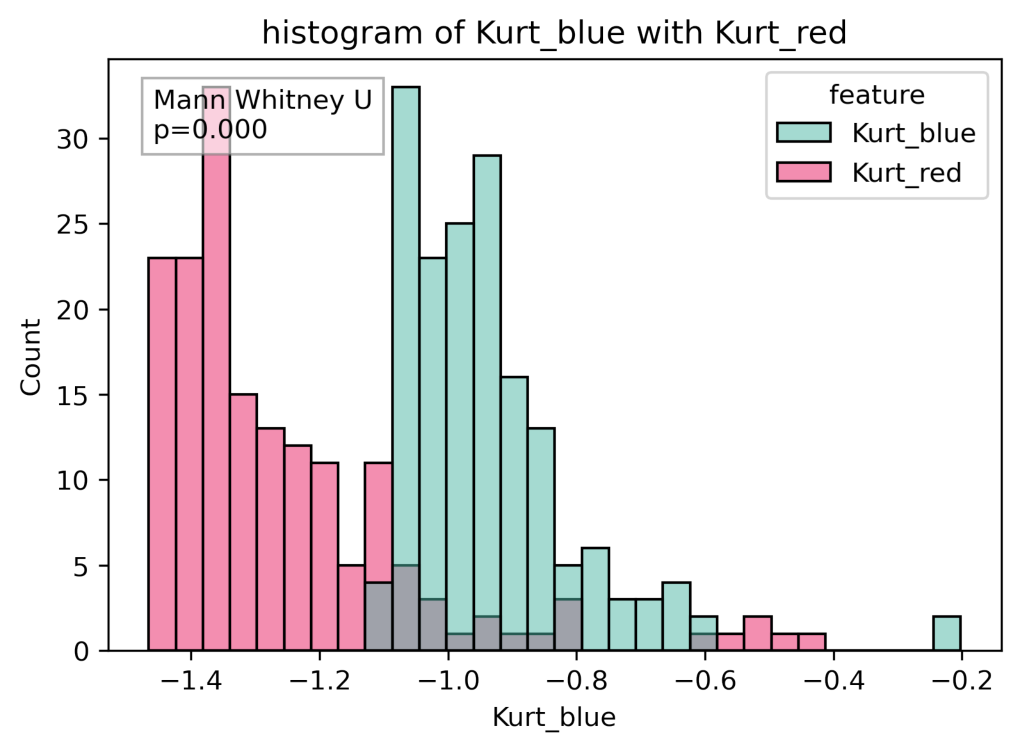

histogram of Kurt_blue with Kurt_red
Mann Whitney U
p=0.000
feature
Kurt_blue
Kurt_red
Count
0
5
10
15
20
25
30
−1.4
−1.2
−1.0
−0.8
−0.6
−0.4
−0.2
Kurt_blue

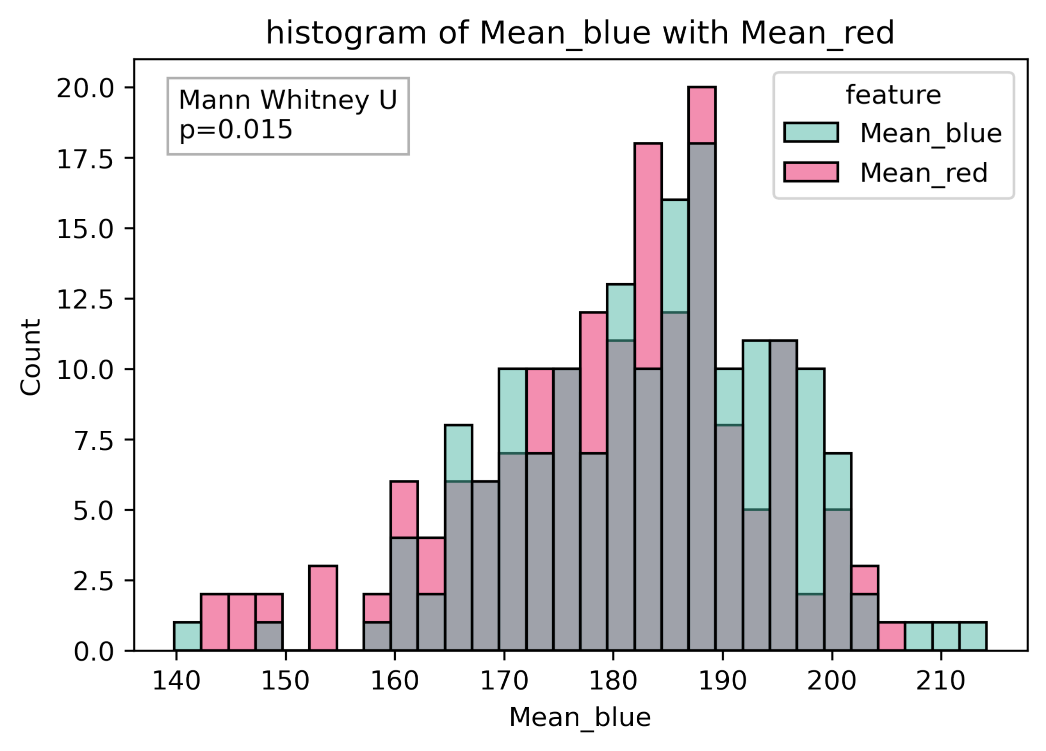
histogram of Mean_blue with Mean_red
Mann Whitney U
p=0.015
feature
Mean_blue
Mean_red
Count
Mean_blue
0.0
2.5
5.0
7.5
10.0
12.5
15.0
17.5
20.0
140
150
160
170
180
190
200
210

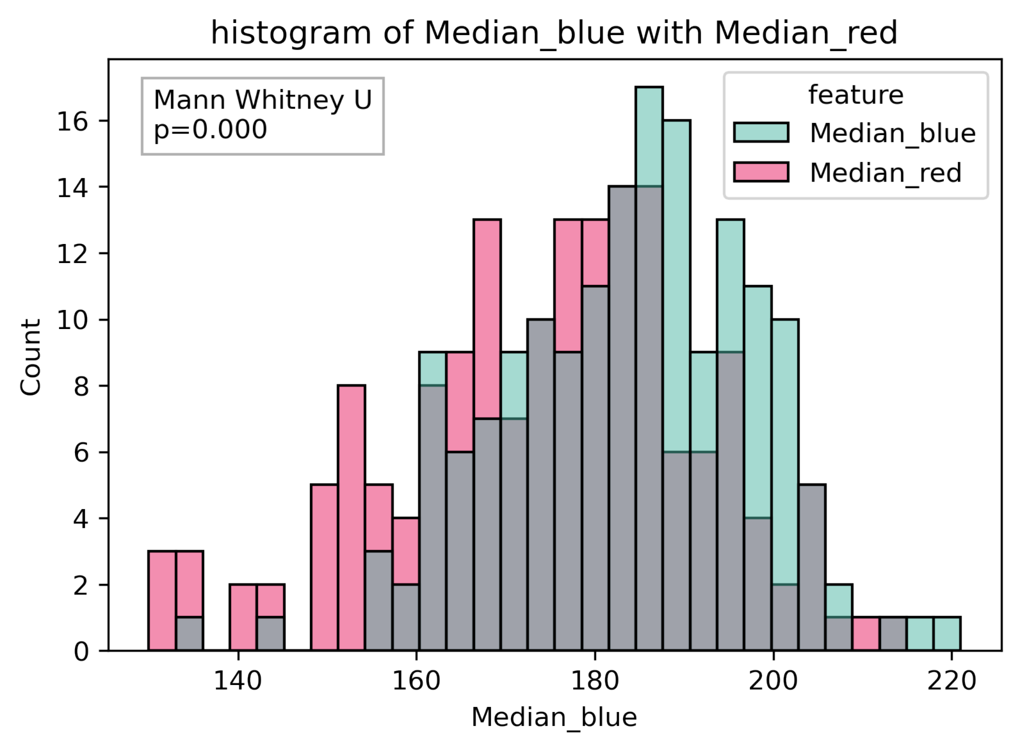
histogram of Median_blue with Median_red
Mann Whitney U
p=0.000
feature
Median_blue
Median_red
Count
Median_blue
0
2
4
6
8
10
12
14
16
140
160
180
200
220

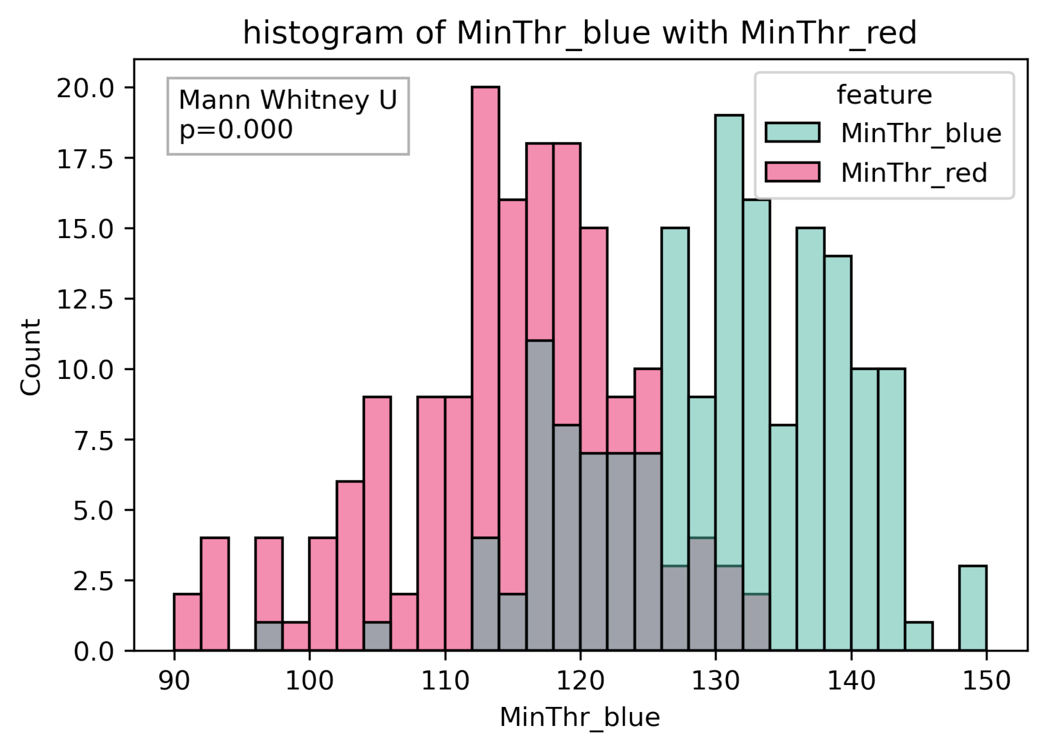
histogram of MinThr_blue with MinThr_red
Mann Whitney U
p=0.000
feature
MinThr_blue
MinThr_red
Count
MinThr_blue
0.0
2.5
5.0
7.5
10.0
12.5
15.0
17.5
20.0
90
100
110
120
130
140
150

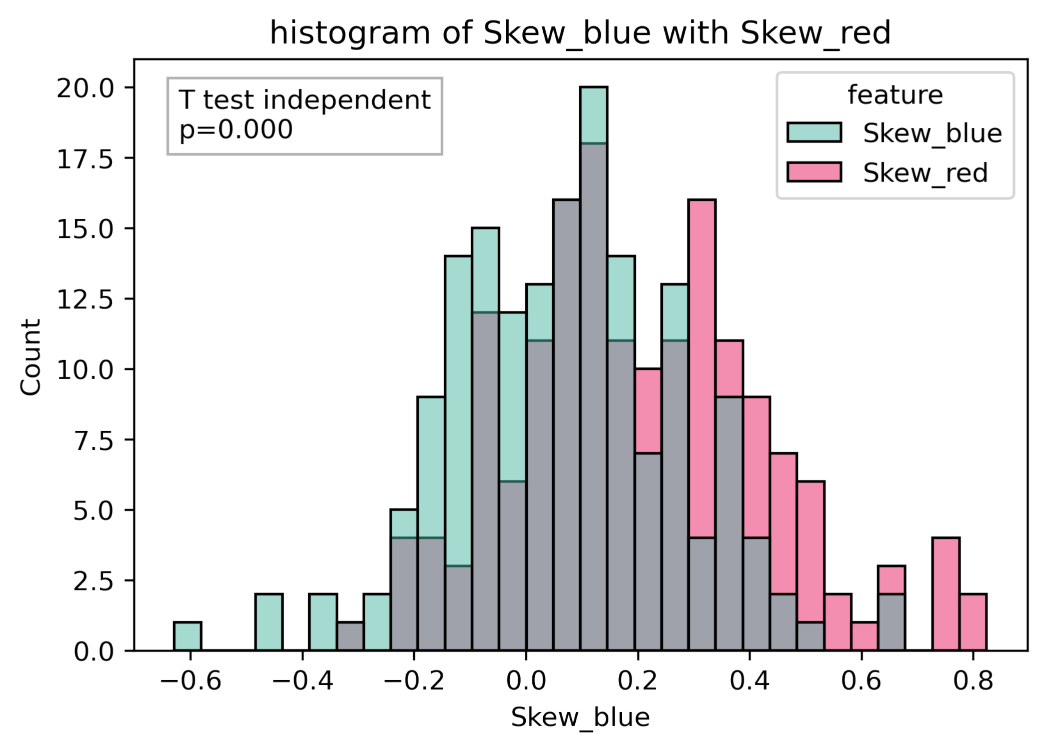
histogram of Skew_blue with Skew_red
T test independent
p=0.000
feature
Skew_blue
Skew_red
Count
Skew_blue
20.0
17.5
15.0
12.5
10.0
7.5
5.0
2.5
0.0
−0.6
−0.4
−0.2
0.0
0.2
0.4
0.6
0.8

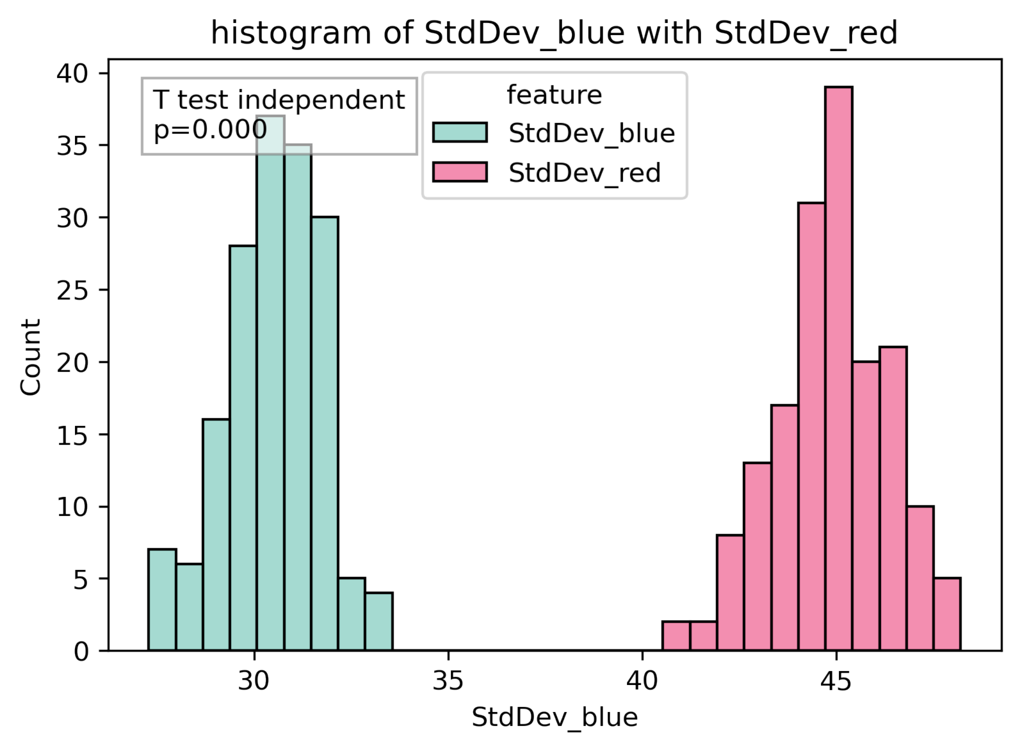

histogram of StdDev_blue with StdDev_red
T test independent
p=0.000
feature
StdDev_blue
StdDev_red
Count
StdDev_blue
0
5
10
15
20
25
30
35
40
30
35
40
45
