# Supplementary material for: Quantitative Histomorphometric Analysis of Collagen Bundles in Masson's Trichrome Stained Rat (Rattus norvegicus) Skin: A Methodological Study
Source: Health Sci Rep. 2026 Mar 8;9(3):e71998. doi: 10.1002/hsr2.71998 (PMC12967519; doi:10.1002/hsr2.71998)
Supplement: Supplementary file 5 — S4 linear regression of features between blue green and red pink. [file HSR2-9-e71998-s003.pdf]

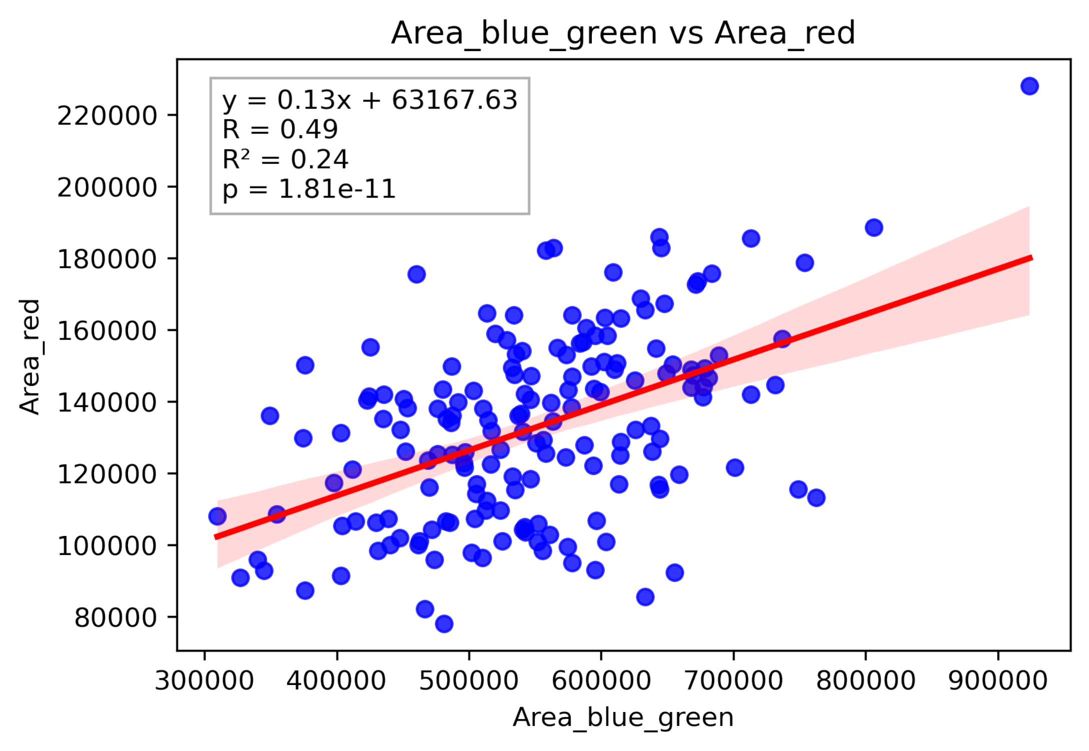

Area_blue_green vs Area_red
y = 0.13x + 63167.63
R = 0.49
R² = 0.24
p = 1.81e-11
220000
200000
180000
160000
140000
120000
100000
80000
Area_red
300000
400000
500000
600000
700000
800000
900000
Area_blue_green

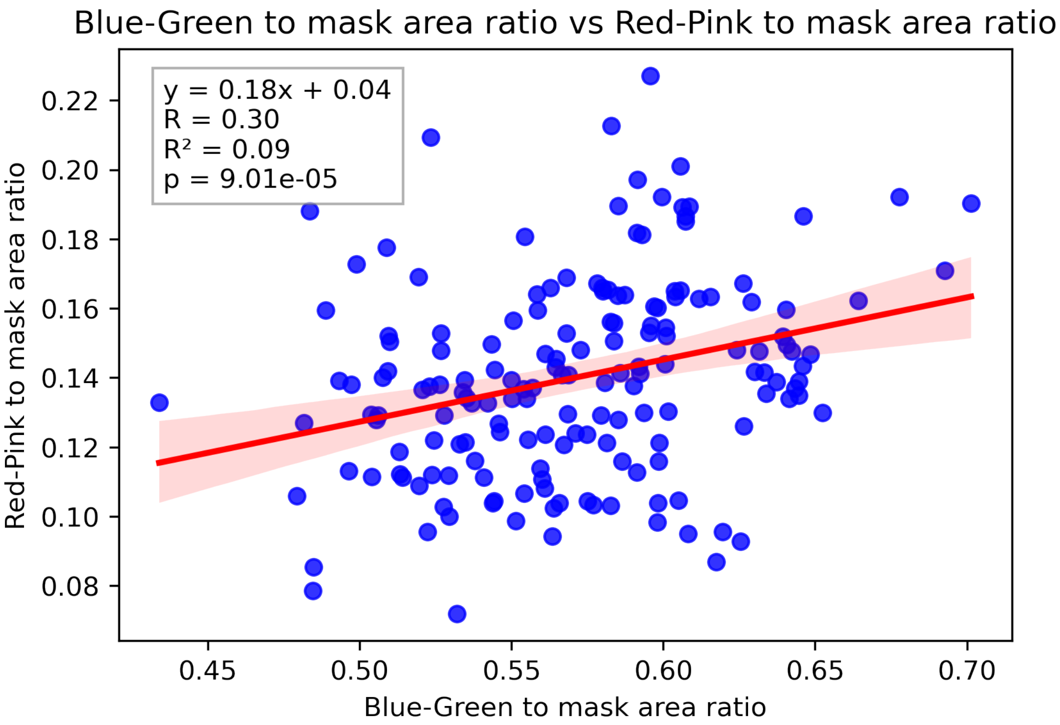

Blue-Green to mask area ratio vs Red-Pink to mask area ratio
y = 0.18x + 0.04
R = 0.30
R^2 = 0.09
p = 9.01e-05
Red-Pink to mask area ratio
0.22
0.20
0.18
0.16
0.14
0.12
0.10
0.08
0.45
0.50
0.55
0.60
0.65
0.70
Blue-Green to mask area ratio

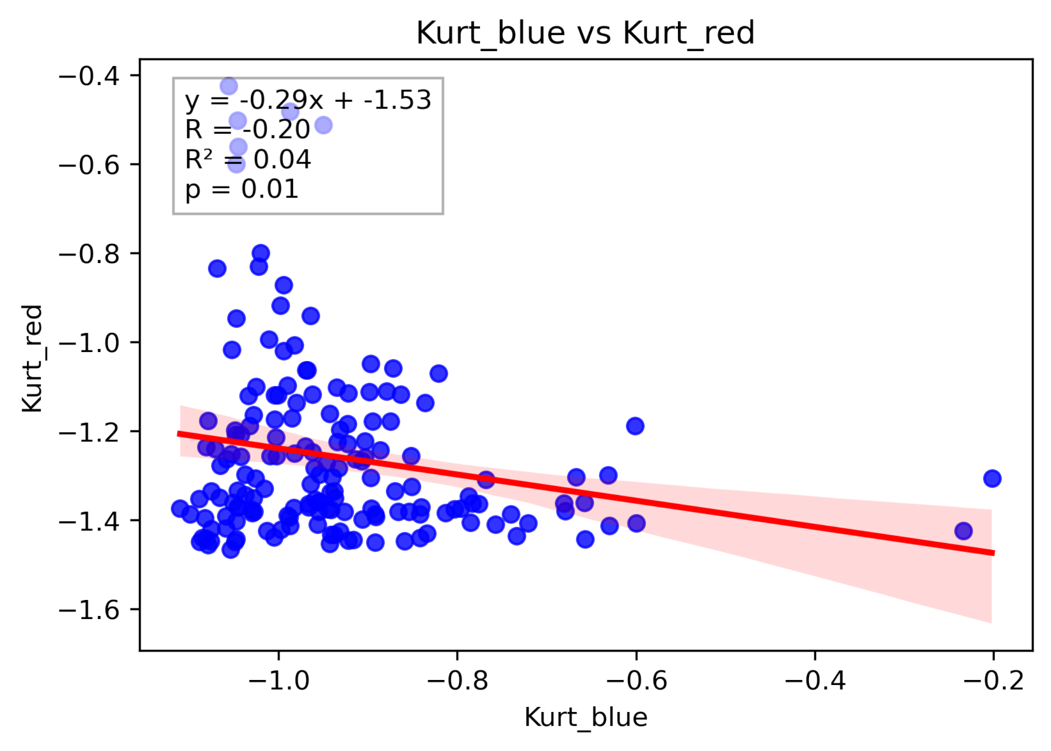
Kurt_blue vs Kurt_red
y = -0.29x + -1.53
R = -0.20
R² = 0.04
p = 0.01
Kurt_red
Kurt_blue
−0.4
−0.6
−0.8
−1.0
−1.2
−1.4
−1.6
−1.0
−0.8
−0.6
−0.4
−0.2

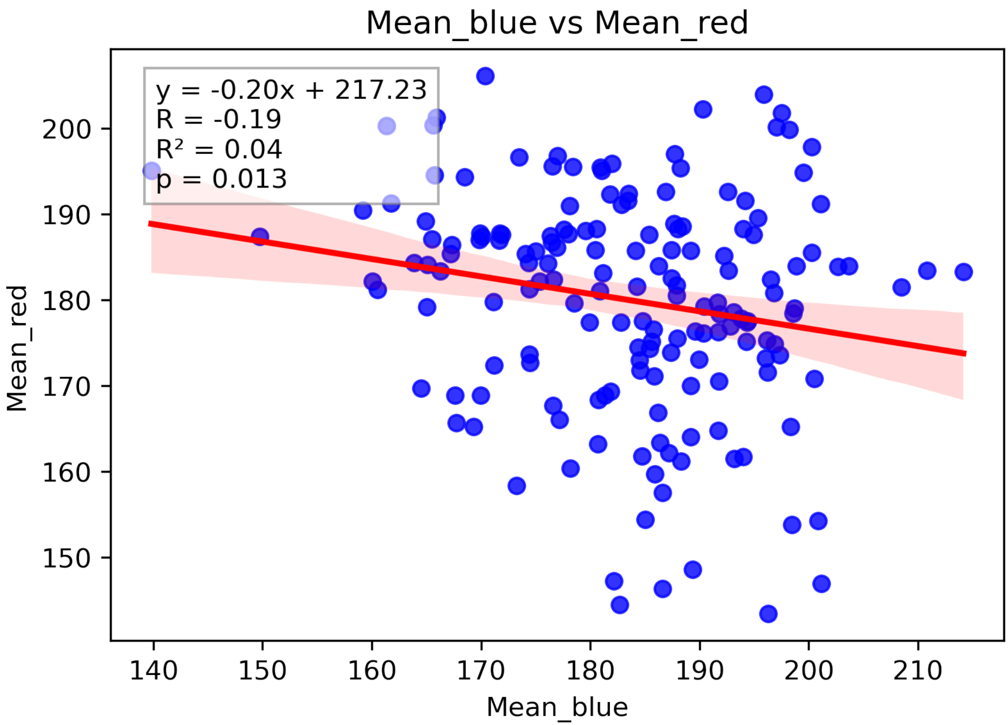

Mean_blue vs Mean_red
y = -0.20x + 217.23
R = -0.19
R² = 0.04
p = 0.013
Mean_red
Mean_blue
200
190
180
170
160
150
140
150
160
170
180
190
200
210

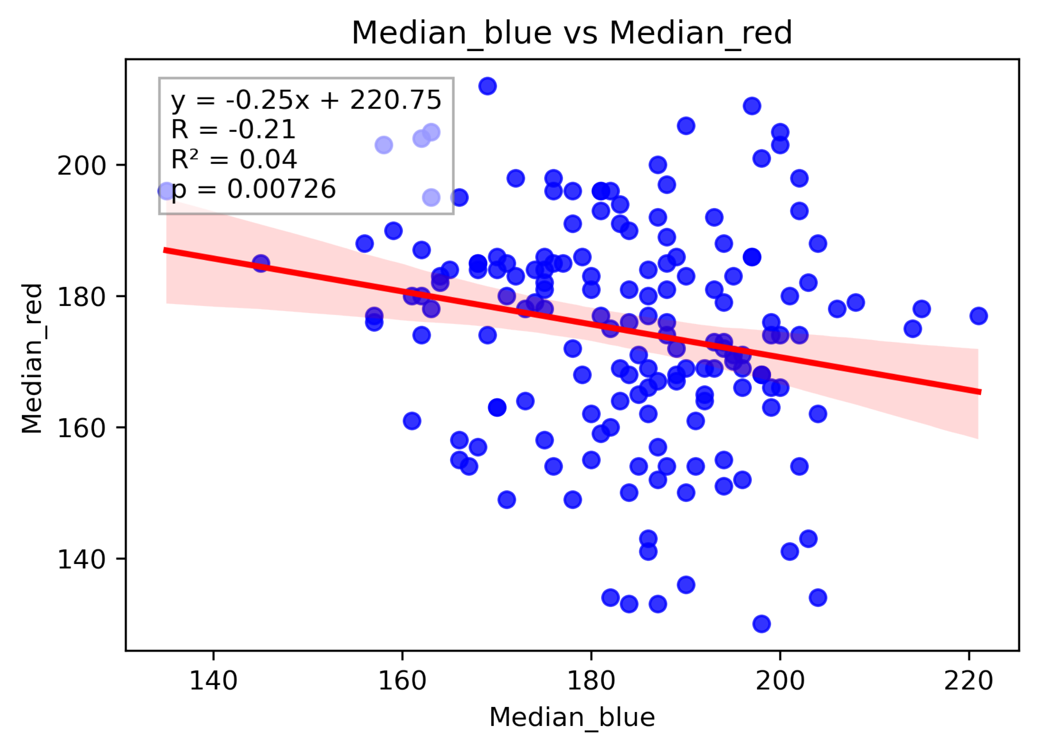
Median_blue vs Median_red
y = -0.25x + 220.75
R = -0.21
R² = 0.04
p = 0.00726
Median_red
Median_blue
140
160
180
200
220

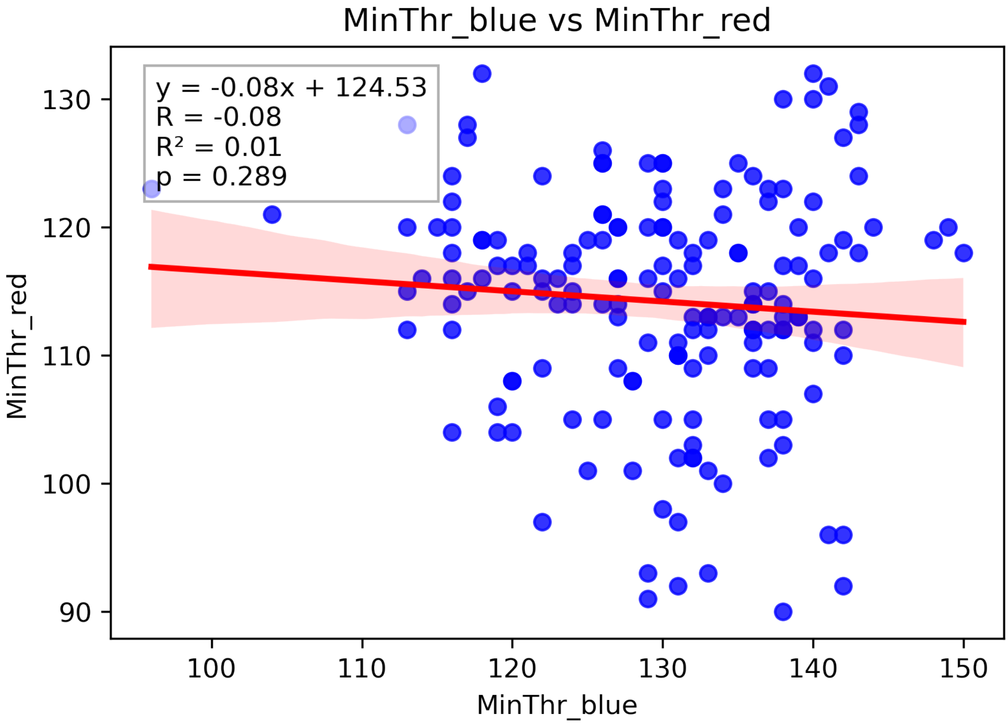
MinThr_blue vs MinThr_red
y = -0.08x + 124.53
R = -0.08
R² = 0.01
p = 0.289
MinThr_red
MinThr_blue
90
100
110
120
130
140
150

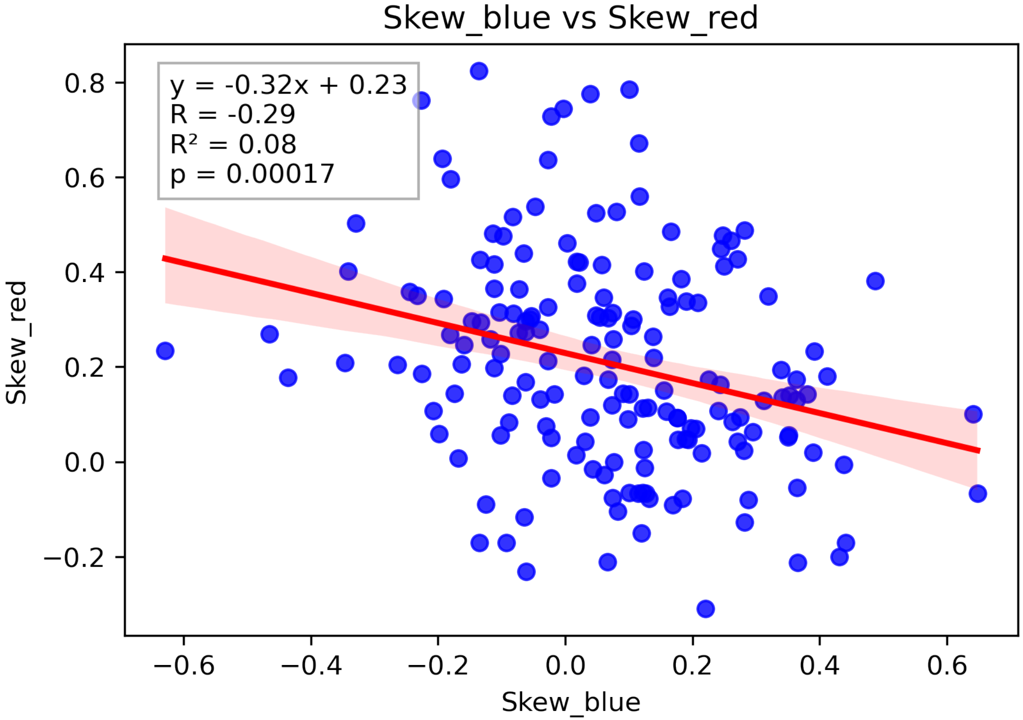
Skew_blue vs Skew_red
y = -0.32x + 0.23
R = -0.29
R² = 0.08
p = 0.00017
0.8
0.6
0.4
0.2
0.0
−0.2
Skew_red
−0.6
−0.4
−0.2
0.0
0.2
0.4
0.6
Skew_blue

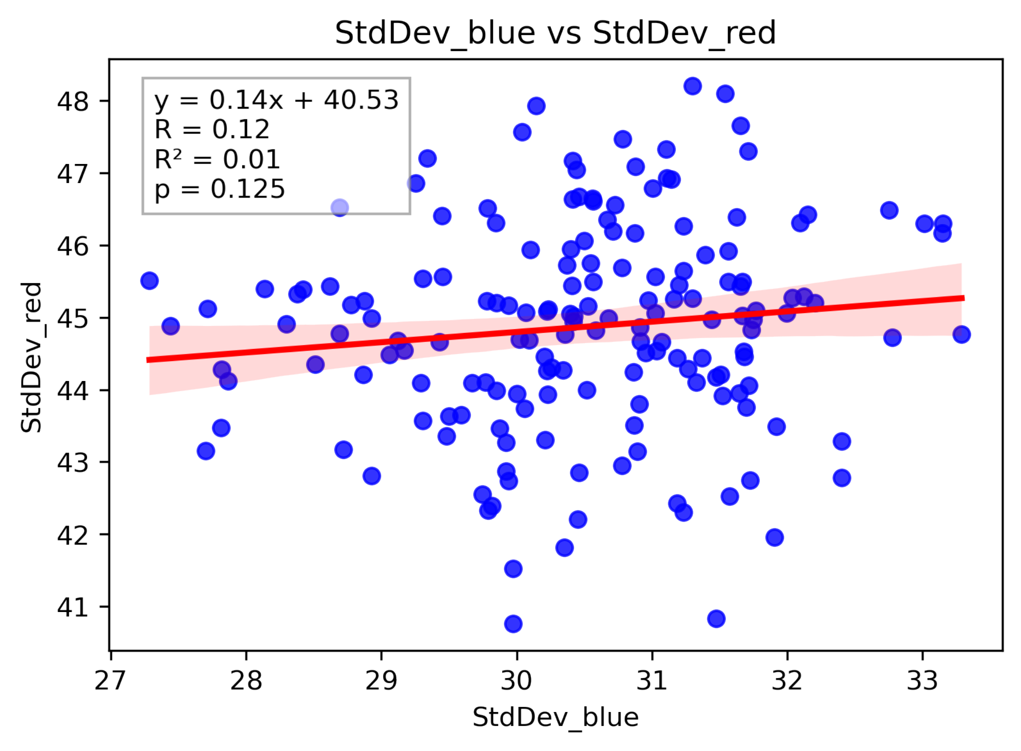
StdDev_blue vs StdDev_red
y = 0.14x + 40.53
R = 0.12
R² = 0.01
p = 0.125
StdDev_red
StdDev_blue
48
47
46
45
44
43
42
41
27
28
29
30
31
32
33
